# The mesoeconomics of abortion: A scoping review and analysis of the economic effects of abortion on health systems

**DOI:** 10.1371/journal.pone.0237227

**Published:** 2020-11-04

**Authors:** Samantha R. Lattof, Ernestina Coast, Yana van der Meulen Rodgers, Brittany Moore, Cheri Poss

**Affiliations:** 1 Department of International Development, London School of Economics and Political Science, London, United Kingdom; 2 Department of Labor Studies and Employment Relations, Rutgers University, Piscataway, New Jersey, United States of America; 3 Department of Women’s and Gender Studies, Rutgers University, Piscataway, New Jersey, United States of America; 4 Ipas, Chapel Hill, North Carolina, United States of America; Queensland University of Technology, AUSTRALIA

## Abstract

**Background:**

Despite the high incidence of abortion around the globe, we lack synthesis of the known economic consequences of abortion care and abortion policies at the mesoeconomic level (i.e. health systems and communities). This scoping review examines the mesoeconomic costs, benefits, impacts, and values of abortion care and policies.

**Methods and findings:**

Searches were conducted in eight electronic databases. We conducted the searches and application of inclusion/exclusion criteria using the PRISMA extension for Scoping Reviews. For inclusion, studies must have examined at least one of the following outcomes: costs, benefits, impacts, and value of abortion care or abortion policies. Quantitative and qualitative data were extracted for descriptive statistics and thematic analysis. Of the 150 included mesoeconomic studies, costs to health systems are the most frequently reported mesoeconomic outcome (n = 116), followed by impacts (n = 40), benefits (n = 17), and values (n = 11). Within health facilities and health systems, the costs of providing abortion services vary greatly, particularly given the range with which researchers identify and cost services. Financial savings can be realized while maintaining or even improving quality of abortion services. Adapting to changing laws and policies is costly for health facilities. American policies on abortion economically impact health systems and facilities both domestically and abroad. Providing post-abortion care requires a disproportionate amount of health facility resources.

**Conclusions:**

The evidence base has consolidated around abortion costs to health systems and health facilities in high-income countries more than in low- or middle-income countries. Little is known about the economic impacts of abortion on communities or the mesoeconomics of abortion in the Middle East and North Africa. Methodologically, review papers are the most frequent study type, indicating that researchers rely on evidence from a core set of costing papers. Studies generating new primary data on mesoeconomic outcomes are needed to strengthen the evidence base.

## Introduction

Despite the high incidence of abortion around the globe, we lack synthesis of the known economic consequences of abortion care and abortion policies. Hence, the economic consequences of abortion and policies affecting abortion provision are poorly understood. In many countries, a shortage of doctors in the public sector, the inability of abortion care-seekers to afford abortions in the private sector, and the aversion by health care workers in the public sector toward abortion have all contributed to the stigmatization of abortion and the lack of access to safe abortion services [1].

In settings where abortion is highly stigmatized, unsafe, and/or illegal, health systems require a significant proportion of resources to treat complications resulting from unsafe abortions [2, 3]. Treatment of complications is often delivered at public tertiary hospitals, where costs are greatest [4, 5]. The widespread dissemination of information through the internet has helped to destigmatize both abortion and contraception, and it has provided health care practitioners and abortion care-seekers with clinical information about fertility control and safe abortion procedures, including medical abortion or ‘abortion pills’ [6].

Whilst the medical abortion combination of mifepristone and misoprostol appear on the World Health Organization’s list of essential medicines, the use of this combination of drugs to perform medical abortions is still limited in low- and middle-income countries [7]. High prices and restrictive regulations, especially in the case of mifepristone, have limited the widespread use of medical abortions. Abortion care-seekers may obtain misoprostol from doctors, pharmacies, and the black market. However, when used by itself, misoprostol may cause more side effects and incomplete abortions compared to when it is combined with mifepristone [8]. In cases where abortion care-seekers take misoprostol alone, the World Health Organization recommends following up with a health care provider to verify the medical abortion was successful [9].

Post-abortion care services, particularly treatment for complications from unsafe abortions, are costly for health systems [10, 11]. These costs tend to be higher to health systems operating in settings where abortion is heavily restricted than settings where elective abortion is liberal and legal [11]. Thus, changes in abortion access and practice have contributed to increasing abortion rates and costs to health systems in the face of highly restrictive national legislation.

This scoping review examines social science literature for studies that have investigated the impact of abortion care (i.e. un/safe abortion, post-abortion care) and abortion policies on economic outcomes at the health system and community levels. After outlining the methods used to conduct the scoping review, we present the mesoeconomic findings. Descriptive statistics characterize the entire inventory of mesoeconomic studies. Economic findings are then presented thematically by costs, impacts, benefits, and value. Finally, we discuss the findings and offer recommendations for future research. Results from complementary microeconomic and macroeconomic analyses as well as a discussion of the role of stigma are presented in separate companion articles [12–14]. Our aim in this article is to address the multiple channels through which abortion can entail economic costs, impacts, values, and benefits to health systems and communities.

## Materials and methods

We took a systematic approach to finding evidence on the economics of abortion by conducting a manageable, transparent, and reproducible scoping review informed by the Preferred Reporting Items for Systematic reviews and Meta-Analyses (PRISMA) extension for Scoping Reviews (PRISMA-ScR) tool and reporting guidelines [15, 16]. The PRISMA-ScR tool is the most up-to-date guidance on conducting scoping reviews and uses a systematic approach to searching, screening, and reporting [16]. A scoping review was preferable to a systematic review, since we sought to examine what is known about the mesoeconomic consequences of abortion care and abortion policies, and we expected to uncover varied evidence on this topic.

As noted in the PICOTS (populations, interventions, control, outcomes, timeframe, setting) in Table 1, studies reporting on qualitative and/or quantitative mesoeconomic data from any world region were considered. For inclusion, studies must have examined at least one of the following mesoeconomic outcomes: costs, benefits, impacts, and/or value of abortion care or abortion policies. Studies must have been published in English, French, Spanish, Dutch, or German between 1 September 1994 and 15 January 2019. In order to maximize heterogeneity in the results, we searched journals from interdisciplinary fields in eight electronic databases (Cumulative Index to Nursing and Allied Health, EconLit, Excerpta Medica Database, International Bibliography of the Social Sciences, JSTOR, PubMed, ScienceDirect, and Web of Science) and supplemented these searches with expert-recommended articles. The searches, application of inclusion/exclusion criteria, screening, and data extraction were conducted using a rigorous protocol and data extraction tools [17]. Search terms appear in Table 2, and the full electronic search strategy for each database appears in the protocol [17]. Searches were completed on 27 January 2019.

**Table 1:** PICOTS criteria used in the scoping review.

PICOTS	
Populations	Communities and health systems in which individuals obtain abortions or post-abortion care
Interventions	Induced abortion (safe/unsafe), post-abortion care, and/or abortion policies
Control	None
Outcomes	Quantitative or qualitative data on: • economic costs of abortion care or abortion policies • economic impacts of abortion care or abortion policies • economic benefits of abortion care or abortion policies • economic value of abortion care or abortion policies
Timeframe	1 September 1994 to 15 January 2019
Setting	Any

**Table 2:** Search terms and their combinations.

1. Abortion terms	2. Economic terms	3. Impact terms
abort*	cost*	cost*
termination of pregnancy	econom*	benefit*
terminate pregnancy	price*	value*
pregnancy termination	financ*	impact*
pregnancy terminations	resource*	
Postabortion	fee*	
post-abortion	tax*	
	expenditure*	
	GDP	
	gross domestic product	
	pay*	
	expens*	

In this review, mesoeconomic outcomes include studies reporting data on partial or total costs at the facility or sub-national level, such as the costs for supplies and for different types of abortion. Partial costs at the national level are also considered within the purview of the mesoeconomic level. For studies reporting on the total savings to health systems, context matters. If estimates were produced using facility and sub-national level data, these estimates are included in our mesoeconomic analyses; when these data are scaled up to present a national cost to the government health system, the data are included in our companion article on the macroeconomics of abortion [14]. Similarly, studies reporting total health system costs to governments are included in our companion article on the macroeconomics of abortion.

Quantitative and qualitative data were extracted on the following categories:

- Background information
- Population
- Details of relevant mesoeconomic outcomes
  ○ Financial cost (the amount paid to obtain or deliver abortion care) or adverse financial outcomes from abortion policies
  ○ Impact (the effect or influence of abortion care or abortion policies)
  ○ Benefit (advantages or profits gained from providing abortion care or implementing abortion policies)
  ○ Value (the importance, worth, welfare gains, or utility of providing abortion care or implementing abortion policies)
- Secondary outcome data on abortion-related stigma, discrimination, and exclusion
- Context in which the study was conducted

These data were used for descriptive statistics and thematic analysis using an inductive approach. Our analysis synthesizes the evidence base and identifies evidence gaps on the economic costs and impacts of abortion care to communities and health systems. Data are reported using a systematic narrative synthesis in which the results are presented narratively and organized thematically, supplemented with tables of descriptive statistics on included studies and their outcomes.

## Findings

### Descriptive statistics

**Fig 1:**
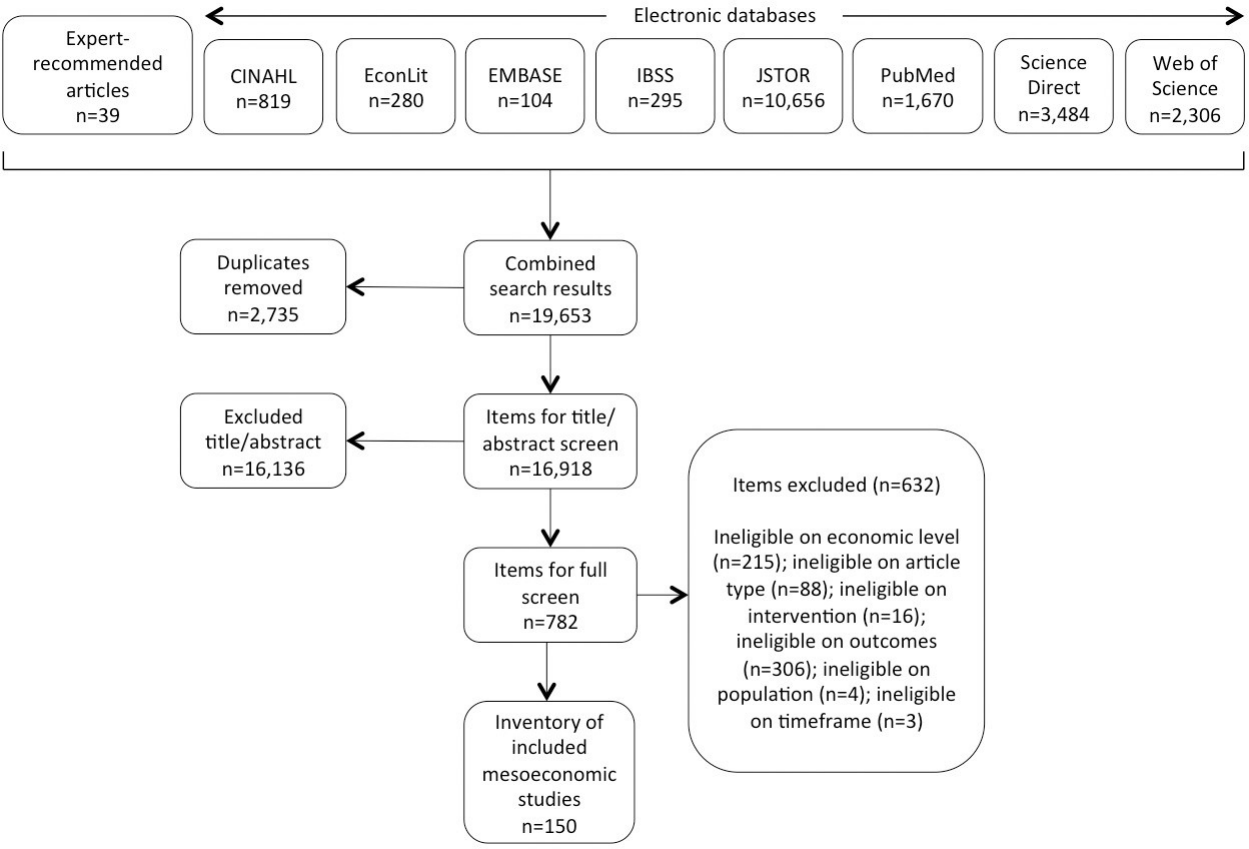
Screening results. PRISMA-ScR flow diagram illustrating the selection of sources of evidence included in the review.

The search generated 19,653 items for screening (Fig 1). After duplicate removal, the 16,918 remaining items were screened for inclusion on the basis of title and abstract. Where exclusion could not be determined on the basis of title and abstract, the authors screened the full text. Decisions were made in favor of an inclusive approach if questions remained. In total, 150 studies met all the inclusion criteria and were included in the scoping review.

Studies came primarily from Africa (n = 31) and Northern America (n = 35) (Table 3). Interestingly, fewer studies focused on countries in Asia (n = 19) even though a large share of the world’s population resides there. Noticeably absent are studies from the Middle East and North Africa. After the United States (n = 32), the United Kingdom (n = 15) and Mexico (n = 7) were most represented in the final inventory.

**Table 3:** Included studies by region and country.

*Region/country*	*# of studies*	*Region/country*	*# of studies*
**Africa**	**31**	**Europe**	**25**
Burkina Faso	1	France	3
Ghana	5	Germany	1
Kenya	1	Ireland	1
Malawi	2	Moldova	1
Nigeria	4	Poland	1
Rwanda	1	Spain	1
South Africa	5	Sweden	1
Tanzania	1	United Kingdom	15
Uganda	2	Multiple countries	1
Zambia	3		
Multiple countries	6	**Latin America & Caribbean**	**19**
		Brazil	1
**Asia**	**19**	Colombia	4
Bangladesh	3	El Salvador	1
China	4	Guadeloupe	1
India	5	Mexico	7
Myanmar	1	Peru	1
Nepal	3	Multiple countries	4
Thailand	2		
Vietnam	1	**Northern America**	**35**
		Canada	3
**Cross-Regional Studies**	**18**	United States (US)	32
Global	9		
Selected countries (including the US)	5	**Oceana**	**3**
Selected countries (excluding the US)	4	Australia	3
		**Total**	**150**

Note: Each data point represents the number of included studies covering the specified country.

The majority of studies were quantitative in nature, with 75 studies relying exclusively on quantitative methods and another 39 studies including both quantitative and qualitative methods (Table 4). Studies most often occurred in countries classified as high-income (44.0%), followed by lower-middle-income countries (17.3%) and upper-middle-income countries (16.0%). The level of geographic coverage varied with nearly one-third of studies examining abortion outcomes within health facilities and the remainder being conducted at the national (n = 31), sub-national (n = 42), local (n = 1), and other (n = 28) levels.

**Table 4:** Characteristics of included studies (n = 150).

	*No*. *Studies*	*Percent*
**Type of Data**		
Quantitative	75	50.0
Qualitative	36	24.0
Both	39	26.0
**Methodology**		
Randomized controlled trial	4	2.7
Controlled clinical trial	2	1.3
Cohort analytic	1	0.7
Cohort (before & after)	2	1.3
Qualitative	28	18.7
Mixed methods	17	11.3
Regression	7	4.7
Review paper	21	14.0
Other	68	45.3
**Country Income Group**		
Low	11	7.3
Lower-middle	26	17.3
Upper-middle	24	16.0
High	66	44.0
Multiple	23	15.3
**Geographical Level**		
National	31	20.7
Sub-national (e.g. state, city)	42	28.0
Local (e.g. village)	1	0.7
Health facility	48	32.0
Other	28	18.7
**Study Population**		
National	16	10.7
Geographical location (e.g. urban/rural, region, facility)	23	15.3
Age (e.g. adolescents)	1	0.7
Status as abortion seeker	33	22.0
Multiple answers from list	31	20.7
Other, specify	21	14.0
Abortion provider	24	16.0
Unclear/unspecified	1	0.7

Studies most frequently reported the costs of abortion provision and policies within health systems and communities (n = 116), followed by mesoeconomic impacts (n = 40) and mesoeconomic benefits and value (n = 27). To facilitate the analysis, studies on mesoeconomic benefits and values are tabulated jointly. The total number of data points (i.e. reported mesoeconomic outcomes) (n = 184) exceeds the number of studies in the final mesoeconomic inventory (n = 150), since some studies presented multiple mesoeconomic outcomes. Each of the economic themes that emerged from the synthesis of data is discussed in further detail below.

### Costs of abortion provision and policies

Approximately three-quarters of studies present data on mesoeconomic costs to health systems (S1 Table). Across these studies, four themes emerged: (1) within health facilities and health systems, the costs of providing abortion services vary greatly, particularly given the range with which services are identified and costed; (2) financial savings can be realized while maintaining or even improving quality of abortion care services; (3) insurance coverage of abortion services is far from universal; and (4) adapting to changes in laws and policies, particularly targeted regulation of abortion provider laws, is costly for health facilities.

*(1) Within health facilities and health systems*, *costs of providing abortion services vary greatly*, *particularly given the range with which services are identified and costed*. Numerous factors influence the costs of providing abortion services in health facilities: the gestational age at which the abortion occurs, the method of abortion (e.g. whether or not anesthesia is required, whether the patient or hospital staff administered medical abortion), the supplies and equipment used, whether contraception is provided, diagnostic tests, management of potential complications, the number of routine follow-up visits required, the number of missed appointments (“no shows”), caseload, frequency of procedures, overhead costs of a hospital stay, the legal environment, regulatory fees, facility type, and facility location [2, 18–28].

The cost incurred by each of these components may vary greatly even within the same health system. In Mexico, the average cost of treating severe abortion complications in public hospitals ranged from US$ 601 to beyond US$ 2,100 [29]. Costs to health facilities are higher in locations with patchwork abortion laws. For example, abortion reforms in Mexico’s Federal District led women from other states to come seek legal abortions there; as a result, facilities providing abortion services in the Federal District experienced increases in workloads and costs [26].

While patients can influence the cost to health facilities of providing abortions through factors such as increases in workloads and missed appointments, the health facility costs of providing abortion services also influence patients. The increased costs of providing a second-trimester abortion compared to a first-trimester abortion require clinics to charge higher fees for the procedure, thus potentially delaying or restricting women’s access to abortion services [30–32]. It is noteworthy that in many areas in the United States, median charges for abortion remained relatively constant over time; this finding may represent providers’ commitments to maintain abortion access and keep services affordable [24].

Among studies that costed or reported costs of abortion services, significant variation exists in how studies report the costs of abortion to health facilities (Table 5) and health systems (Table 6). Studies report on the costs of different methods of abortion (e.g. manual vacuum aspiration, dilation and curettage, medical abortion, surgical abortion), the costs of abortion in specific locations (e.g. in a nurse-led clinic, in a tertiary hospital), and the type of abortion service (e.g. abortion at 20-weeks gestation, post-abortion care, treatment for septic abortion, treatment of incomplete abortion). Some researchers specify all of the direct and indirect components that are involved in the delivery of abortion services and cost each of these components, making it easy to understand how the total cost was derived [33]. Other researchers do not. Some researchers report the costs for an abortion without specifying the type of abortion (e.g. medical, surgical, early/late gestational age). Other researchers are quite specific about the type of service being costed (e.g. manual vacuum aspiration for treating incomplete abortion patients), especially researchers who reported costs for different pilot programs in the United Kingdom [34–36].

**Table 5:** Costs of abortion services in health facilities.

World Region	Country	Abortion Service	Health facility costs
Africa		Post-abortion care (PAC)	$392 per case (in 2007 international dollars) [37]
			Average cost per patient (in 2006 international dollars) was $294.35 in Africa and $315.16 in sub-Saharan Africa [38]
	Burkina Faso	Treatment of abortion complications	Mean cost per patient was US$ 45.86 (ranging from US$ 51.09 in the tertiary teaching hospital to US$ 36.50 in the secondary level hospital); incomplete abortion (US$ 23.71) and hemorrhage (US$ 26.30) were the least expensive services per case; uterus perforation (US$ 73.76) and infection/sepsis (US$ 94.39) were the most expensive services at the tertiary level teaching hospital [5]
	Ghana	Manual vacuum aspiration (MVA)	US$ 11–33 in public regional/district level facilities, US$ 54–163 in private hospitals and clinics, and US$ 3–27 in private maternity homes [39]
	Kenya	Sharp curettage for treating incomplete abortion patients	Average cost of US$ 15.25 per patient in one district hospital [40]
		MVA for treating incomplete abortion patients	Average cost of US$ 5.19 per patient in one district hospital [40]
	Nigeria	Treatment for septic abortion	Average cost of US$ 223.11 per patient [40]
		PAC	Average cost of treatment for a simple case was US$ 70, a case with moderate complications was US$ 112, a case with severe complications was US$ 258; estimated per-case costs of each procedure type varied widely across facilities with MVA/ electric vacuum aspiration (EVA) ranging from US$ 43 to US$ 141, dilation and curettage (D&C)/ dilation and evacuation (D&E) ranging from US$ 44 to US$ 114, misoprostol alone ranging from US$ 48 to US$ 129, and expectant management ranging from US$ 32 to US$ 104 [41]
		Treatment of abortion complications	Average of US$ 7.50 per patient [40]
		MVA vs. D&C	MVA to perform an abortion cost 3,446 naira [2]
			D&C to perform an abortion cost 3,090 naira [2]
	Rwanda	PAC	Per-case treatment cost $239 on average at referral hospitals, $93 at district hospitals, and $72 at health centers [42]
	South Africa	D&E	Least costly (US$ 88.89 per woman seen) and most cost-effective at US$ 91.17 per complete abortion [43]
		Second-trimester induction with misoprostol	Combined regimen cost $298.03 and misoprostol alone cost $364.08 [43]
		Medication abortion	Average cost per medication abortion was $63.91 (52.32–75.51) [44]
	Uganda	Induced abortion	
		Treatment of abortion complications (in hospital)	Average of US$ 7.50 per patient [40]
	Zambia	Medical abortion	US$ 33 [3]
		Treating incomplete abortion	US$ 33 [3]
		MVA	US$ 39 [3]
		PAC for unsafe abortion (at university teaching hospital)	US$ 109,811 per year (13 times greater than the cost of safe abortion) [3]
Asia	China	Ultra-early medical abortion (hospital-administration group)	Mean cost expenditure per participant was US$ 40.12 [18]
		Ultra-early medical abortion (self-administration group)	Mean cost expenditure per participant was US$ 1.96 [18]
		Surgical abortion	Accounting only for initial costs, surgical abortion cost CNY 367.56 ± 21.31; when the subsequent costs of examination and treatment due to incomplete abortion and bleeding within the period of 2-week follow-up were added, it cost CNY 375.16 ± 12.81 [33]
		Medical abortion	Accounting only for initial costs, medical abortion cost CNY 279.25 ± 9.48; when the subsequent costs of examination and treatment due to incomplete abortion and bleeding within the period of 2-week follow-up were added, on average it cost CNY 379.03 ± 27.75 [33]
	India	Abortion	Charges ranged from Rs. 135–534 (average Rs. 370) for public providers and Rs. 394–649 (average Rs. 497) for private providers [19]
	Thailand	MVA and sharp curettage (in hospital)	Total cost for MVA was US$ 54.67 per procedure [45]
			Total cost for sharp curettage was US$ 153.97 per procedure [45]
Europe	France	Surgical abortion with anesthesia (at a regional maternity hospital)	€ 562 per abortion, includes direct and indirect costs [46]
	Sweden	First visit to clinic for of early medical abortion	Direct costs were € 58.3 per procedure; the intervention treatment (delivered by nurse-midwives) cost € 45 [47]
	United Kingdom	Medical abortion	Cost £ 343 and used 8% less of National Health System resources than vacuum aspiration (£ 374) [48]
			Full cost of an outpatient medical termination of pregnancy procedure (including all Trust overheads and laboratory tests/scans) was £ 462 per patient [35]
		Nurse-led clinic	Calculated annual cost of the medically led clinic was £ 37,495; if run in its present form by a G-grade nurse, the annual cost would be £ 25,943 [49]
		Surgical abortion (in primary health care trust)	Cost of a day case ranged from £ 462 to £ 578 per patient; cost of a local anaesthetic outpatient surgical abortion service could be reduced to £ 366 per patient; if this involved outpatient consultation the cost was £ 217 per patient that could be further reduced to £ 177 if the nurse telephone clinic was used [50]
			Full cost of a surgical termination of pregnancy (day case) (including all Trust overheads and laboratory tests/scans) was £ 482 or £ 578 per patient [35]
		Local anaesthetic outpatient surgical termination of pregnancy clinic	Full cost (including all Trust overheads and laboratory tests/scans) was £ 366 per patient [35]
		Outpatient consultation on termination of pregnancy	£ 217 per outpatient consultation (including all Trust overheads and laboratory tests/scans) [35]
		Legal, medical abortion in clinic with complications	Mean cost was $165 [51]
		Legal, medical abortion in clinic without complications	Mean cost was $132 [51]
		Legal MVA in a clinic with complications	Mean cost was $223 [51]
		Legal MVA in a clinic without complications	Mean cost was $197 [51]
		Legal MVA in hospitals with complications	Mean cost was $467 [51]
		Legal MVA in hospitals without complications	Mean cost was $374 [51]
		MVA PAC in hospitals with complications	Mean cost was $563 [51]
		MVA PAC in hospitals without complications	Mean cost was $231 [51]
		D&C in hospitals with complications	Mean cost was $821 [51]
		D&C in hospitals without complications	Mean cost was $657 [51]
		D&C PAC in hospitals with complications	Mean cost was $2,301 [51]
		D&C PAC in hospitals without complications	Mean cost was $458 [51]
		Two regimes of mid-trimester medical termination of pregnancy	Median cost was £ 420 in the mifepristone group vs. £ 885 in the Prostaglandin E₂ group [52]
		New outpatient service for early medical termination under 7 weeks’ gestation	Estimated cost per case in the first year was £ 156 which represented a considerable cost saving compared to £ 498 for surgical termination, £ 423 for inpatient medical termination [36]
Latin America		Treatment of incomplete abortion	Can absorb more than 50% of facilities’ obstetric and gynaecologic budgets [53]
		PAC	Cost (in 2007 international dollars) was $430 per case [37]
			Average cost per patient (in 2006 international dollars), including overhead and capital costs, was $223.25 [38]
		Treatment of PAC	Per-patient cost was US$ 94 [54]
	Colombia	PAC (complicated)	
		PAC (uncomplicated) with MVA	Average cost was $231 [55]
		PAC (uncomplicated) with D&C	Average cost was $458 [55]
		Legal, medical abortion in clinic with complications	Cost $165 [55]
		Legal, medical abortion in clinic without complications	Cost $132 [55]
		Legal, MVA in clinic with complications	Cost $223 [55]
		Legal, MVA in clinic without complications	Cost $197 [55]
		Legal MVA in hospitals with complications	Cost $467 [55]
		Legal MVA in hospitals without complications	Cost $374 [55]
		MVA post-abortion care in hospitals with complications	Cost $563 [55]
		MVA PAC in hospitals without complications	Cost $231 [55]
		D&C in hospitals with complications	Cost $821 [55]
		D&C in hospitals without complications	Cost $657 [55]
		D&C PAC in hospitals with complications	Cost $2,301 [55]
		D&C PAC in hospitals without complications	Cost $458 [55]
	El Salvador	MVA procedure and hospital stay	Total cost was US$ 54 [25]
		Sharp curettage procedure	Total cost was US$ 62 [25]
North America	Canada	Abortion by vacuum aspiration (in hospital)	Average case cost Can$ 419.00 [34]
		Abortion by vacuum aspiration (in clinic)	Average case cost Can$ 233.83 [34]
		Treatment of bleeding after abortion	Average hospital case cost Can$ 646.68 [34]
		Treatment of infection after abortion	Average hospital case cost Can$ 1,021.00 [34]
	Mexico	Abortion with dilatation and curettage	Average cost per abortion was US$ 143 [29]
		Abortion with MVA	Average cost per abortion was US$ 111 in three public hospitals and US$ 53 at a private clinic [29]
		Medical abortion with misoprostol alone	Average cost was US$ 79 [29]
		Treatment of severe abortion complications (at public hospitals)	Average cost ranged from US$ 601 to over US$ 2,100 [29]
	United States	Medical abortion	Mean total episode cost (including direct, indirect, and space costs) was $346 (range $252- $460) [56]
			In 2001–2002, charges for a mifepristone abortion and for a methotrexate abortion were $490 and $438, respectively. Providers who used 600 mg of mifepristone charged $74 more, on average, than providers who used 200 mg. More than two in five providers (43%) charged between $400 and $499 for mifepristone, and 38% charged $500 or more [57]
			In 2011, the median charge was similar to surgical abortions at 10 weeks’ gestation at $500; the median charge for an abortion at 20 weeks’ gestation in 2011 and 2012 was $1,350 [24]
			Median cost was $450 [58]
		Abortion from non-hospital providers	In 1993, on average, non-hospital providers charged US$ 604 for an abortion at 16 weeks and US$ 1,067 at 20 weeks [23]
		Surgical abortion	In 2011 and 2012, the median charge for a surgical abortion at 10 weeks gestation was US$ 495; clinics charged the least for a surgical abortion at 10 weeks’ gestation (US$ 450) and physicians’ offices charged the most (US$ 550) [24]
			Median costs were US$ 425 for a first trimester surgical abortion and US$ 900 for a second-trimester abortion [58]
		Abortion at 20-weeks gestation	Median charge was US$ 1,195 (in 2014) [59]
		Abortion-related emergency department visits	Average costs were US$ 4,719 [60]

Note: Many studies do not report whether the cost presented refers to the time of publication or the time the study was conducted. Other studies do not specify the currency used. Without these details, we cannot accurately convert the currencies into a standard currency (e.g. US$) to allow for direct comparisons across studies.

**Table 6:** Costs of abortion services in health systems.

World Region	Country	Abortion Service	Health system costs
Africa	Nigeria	PAC	Total annual cost for the 17 facilities is US$ 274,015; all 79 PAC-providing public hospitals in the 3 included Nigeria states currently spend an estimated US$ 807,442 annually [41]
	Uganda	Induced abortion	Costs $23.6 million in direct medical costs annually [61]
Latin America	Colombia	PAC (complicated)	For every 1,000 women receiving PAC instead of legal abortion, 16 women had unnecessary complications costing US$ 48,000. If the remainder of PAC cases currently observed were replaced with legal abortion (medical or MVA), the health system would save an additional $163,000 dollars and prevent 16 complications per 1,000 abortions [55].
		PAC (uncomplicated) with D&C	Could save $177,000 (per 1,000 women) from baseline by replacing D&C with MVA [55]
North America	Canada	Abortion by vacuum aspiration (in hospital)	Direct cost was Can$ 842.63 [34]
		Abortion by vacuum aspiration (in clinic)	Direct cost was Can$ 518.77 [34]
		Procedure with mifepristone-misoprostol	Direct cost was Can$ 361.93 [34]
		Procedure with methotrexate-misoprostol	Direct cost was Can$ 385.77 [34]

In settings where individuals seek unsafe abortions, immense health system resources are required to treat complications resulting from unsafe abortions [2, 3]. Public health services often pay for treatment of complications at tertiary hospitals, where costs are greatest [4, 5]. For example, in Ouagadougou, Burkina Faso, the average cost of treating abortion complications in a tertiary hospital (US$ 51.09) was 40% higher than the cost in a secondary-level hospital (US$ 36.50) [5]. In Nigeria, while clinic-based manual vacuum aspiration and hospital-based manual vacuum aspiration are equally effective, hospital-based manual vacuum aspiration is the more costly option [62]. When possible, shifting to the provision of simpler procedures to lower-level hospitals or primary clinics would thus minimize costs and lead to financial savings among health systems.

*(2) Financial savings can be realized while maintaining or even improving quality of abortion care services*. Reducing costs to health facilities and health systems does not mean reducing the quality of care. For example, without sacrificing quality, it is possible to reduce costs by choosing a local generic abortifacient over an imported one [63]. Health systems can also reduce costs by shifting the location in which abortion care services are provided. In Mexico, shifting from hospital-based manual vacuum aspiration to clinic-based manual vacuum aspiration would save the health system nearly US$ 100,000 per 1,000 women [64]. In the United Kingdom, shifting outpatient consultations to a nurse telephone clinic service could reduce costs per patient by 18% while simultaneously providing patients with greater convenience [35]. Using a local anesthetic outpatient clinic reduced use of the operating room to one streamlined abortion patient list per week, thus generating approximately GB£ 60,000 in annual savings [65]. Health facilities can even improve service delivery whilst reducing their costs of service delivery. In two Latin American study sites, reorganizing services to an outpatient basis resulted in reductions in the average length of patient stay and significant financial savings that facility administrators were then able to pass on to patients via fee reductions [53].

With treatment of post-abortion complications consuming a significant proportion of health system resources in many settings [66], decentralizing services and legalizing abortion can produce substantial financial savings. Financial models from Uganda show that moving from a conventional, legally restrictive setting to a planned, decentralized, legally permissive setting decreased the mean cost per unsafe abortion complication case by 86% from US$ 45 to US$ 6 [11]. Offering menstrual regulation, a method of fertility control, care can also reduce the cost of treatment for severe abortion complications, since menstrual regulation care costs 8–13% of the cost of treating severe abortion complications [67]. When feasible and appropriate, service delivery for post-abortion care can be moved from an inpatient basis to an outpatient basis. Outpatient manual vacuum aspiration for post-abortion care is less expensive than inpatient dilation and curettage, according to findings from multiple studies in Africa and Latin America [41, 68].

Given that clinical staff costs tend to be highest for physicians, facilities in which abortion services are provided by a range of providers (e.g. nurse practitioners, physicians’ assistants) were able to keep staff costs low without sacrificing time with patients [56]. In the United Kingdom, a nurse-led service for termination of pregnancy could maintain the clinic while saving nearly 40% in costs annually compared to a medically-led clinic with a staff-grade doctor [49]. However, task shifting has logistical and financial costs that can raise health faculties’ expenses in the short-term [69]. Removing administrative barriers that limit trained midwives’ ability to provide routine post-abortion care services and implementing a policy on task shifting could free up physicians to focus on more complicated cases [41]. Yet, the setting (e.g. rural facility) and context (e.g. supply chain, sustainability) must be considered when assessing the feasibility of task shifting. For example, low-volume, low-income nurse midwives in Ghana reported less sustainable access to manual vacuum aspiration than physicians [39].

Costs of providing medical abortion and surgical abortion can be quite similar in some settings, like China [70]. As a result, abortion providers do not need to factor in remuneration when recommending a method to patients. In other settings, however, the costs of providing medical and surgical abortion differ. Surgical abortion in the British National Health Service cost more than medical abortion due to inpatient standard costs and used 8% more resources than medical abortion [51, 71]. Costs and savings from surgical abortion techniques also vary by setting. Numerous hospitals in Asia and Latin America found manual vacuum aspiration to produce lower costs to health facilities than sharp curettage [25, 45]. In El Salvador, a manual vacuum aspiration procedure and hospital stay (US$ 54) cost 13% less than sharp curettage and reduced patients’ time in hospital by 28% [25]. In some African countries, the reverse was found: dilation and evacuation was less expensive than medical abortion in South Africa [72].

(3) *Insurance coverage of abortion services is far from universal*. Health insurance and public health systems effectively disincentivize abortion outright or disincentivize certain methods of abortion, particularly medical abortion. Because Australian insurance companies that provide medical indemnity initially assessed the risks of medical and surgical abortions to be the same, physicians considering incorporating medical abortion into their practices experienced a prohibitive increase in their medical indemnity insurance [73]. Once this issue was resolved in 2014, medical abortion access in rural areas improved. In some countries, including Germany and the United Kingdom, the public health systems have not covered the costs to physicians and health facilities of providing medical abortion using Mifegyne (RU 486) [74]. When combined with a requirement for special licenses to offer medical abortion services and the high costs of purchasing Mifegyne, the economic disincentives resulted in surgical abortion becoming the more economic method [74]. These decisions limit patients’ access to the full range of abortion care services.

In the United States, private insurance coverage of abortion is restricted in some states, and not all clinics accept third-party payers [75]. When subsidized or public insurance programs like Medicaid exist, staff members at abortion facilities may play an important yet underutilized role in helping patients enroll [76]. Even when women can obtain Medicaid coverage of abortion, providers are not guaranteed reimbursement for qualifying abortions. In six states where Medicaid coverage of abortion is limited to cases of rape, incest and life endangerment, providers were reimbursed for only 41.6% of abortions that should have qualified for Medicaid reimbursement [58].

Policies to implement safe abortion services in the public sector can backfire if provider reimbursement is insufficient. Following passage of the Safe Abortion Service Guidelines of 2016 in Nepal, provider reimbursement for free abortion services under the national government scheme was less profitable than abortion services in which clients paid out of pocket [77]. Financial incentives could motivate providers to perform abortions in the private sector rather than the public sector, thus reducing access to abortions in public facilities.

*(4) Adapting to changes in laws and policies*, *particularly targeted regulation of abortion provider laws*, *is costly for health facilities*. Following implementation of a new targeted regulation of abortion provider law in Texas, the cost of providing an abortion increased: from 2001 to 2006, the cost of providing an abortion at 20 gestational weeks increased by 37% (US$ 454) compared to other states [78]. Laws can also target the cost of providing a particular type of abortion. In Texas, House Bill 2 increased the cost of medical abortion in most facilities by requiring providers to choose between a drug regimen that was considerably more expensive than the evidence-based regimen or a drug regimen supported by limited evidence [79].

### Impacts of abortion provision and policies

Among the 40 studies that reported economic impacts of abortion at the mesoeconomic level (S2 Table), five themes emerged: (1) limited resources negatively affect health facilities’ ability to meet client demand and to provide quality abortion care services; (2) American policies on abortion economically impact health systems and facilities both domestically and abroad; (3) a disproportionate amount of health facility resources are required to provide post-abortion care; (4) switching abortion techniques in health facilities can have positive or negative economic impacts, depending on the reason behind the change; and (5) complex health systems impact the abortion-related trajectories of pregnant people.

*(1) Limited resources negatively affect health facilities’ ability to meet client demand and to provide quality abortion care services*. Studies examining the economic impact of limited resources for abortion focused on the impact of staff shortages, insufficient physical resources, low Medicaid reimbursement rates, and costly regulations. Shortages of trained staff affected patient care in countries of all income levels. In France, a hospital lacked trained staff to meet patients’ needs for psychological help [80]. In Kenya, gaps in capacity resulted in longer hospital stays and increased costs of care to the health system [81]. In Malawi, staff shortages resulted in one or two nurses covering up to 70 patients, the discontinuation of manual vacuum aspiration, and limited on-the-job training [82]. A large and increasing proportion of unsafe abortion admissions can stretch human resources even further, leading health care staff to likely prioritize the easier option over the patient’s best interests [82].

Shortages of physical resources, such as manual vacuum aspiration instruments, led health care providers in Malawi to automatically resort to dilation and curettage even though dilation and curettage is more expensive, is slower and more difficult to perform, requires more staff, and results in more in-patients [82]. Medication stock-outs due to poor supply chain management resulted in women in Nepal being denied legal abortions [77].

Financially, rates of reimbursement vary. However, some providers in the United States experienced Medicaid reimbursement rates so low that the rates would put providers out of business: one provider was reimbursed only US$ 212 for an abortion that cost US$ 420 [58]. To minimize the financial impact of Medicaid’s insufficient reimbursement rates, providers depended on abortion funds, minimized their work with Medicaid, or avoided Medicaid outright by absorbing up to US$ 60,000 annually in free or reduced-cost services [58].

Due to costly regulations, existing health facility resources may be stretched to the point where facilities must accommodate a smaller clientele or close their doors. In the United States, requiring abortions to be performed in ambulatory surgical centers would cost a facility over US$ 750,000 to comply with the regulations’ physical renovations; as such, these costly requirements may result in providers stopping abortions or significantly raising the prices so that quality abortion care is beyond some women’s reach [75].

*(2) American policies on abortion economically impact health systems and facilities both in the United States and abroad*. Domestically, abortion legislation hinders or prevents health providers from performing abortions by influencing the demand of abortion care and increasing the costs of care. The implementation of House Bill 2 in Texas led clinics to close and increased the average clinic service population from 150,000 clients to 290,000 clients [83]. This increase in service population was felt especially by urban clinics. In Dallas-Fort Worth, the average service population increased by 250,000 clients [83]. As a result of clinic closures, the remaining clinics were unable to meet demand for abortion services and deliver the same quality of care as before the law [84]. In North Carolina, abortion providers attempted to minimize the burden of new regulations on patients by implementing telephone counseling in place of two in-person clinic visits [85]. Telephone counseling required significant adaptations, including hiring additional nurses, developing call-center infrastructure, and changing scheduling and work tasks. By explicitly deciding to absorb the financial burden of these changes rather than pass them on to patients, providers worked more hours uncompensated or fundraised to support the increased costs [85]. Clinics are occasionally unable to absorb the financial burden of abortion regulations, particularly when regulations require costly physical renovations. In these cases, providers may stop performing abortion or may increase their prices beyond some clients’ reach [75].

Abroad, the economic impact of American abortion policies extends beyond abortion care. Funding cuts due to financial restrictions commonly referred to as the Global Gag Rule resulted in disruptions to the provision of sexual and reproductive health services in numerous countries, and clinic closures affected hundreds of thousands of underserved clients whose needs were not met by public health services [86, 87]. In addition to staff terminations and clinic closures that may particularly affect rural clinics, the Global Gag Rule resulted in the termination of contraceptive supply shipments in 29 countries and programmatic cuts (e.g. to maternal health and well baby care, youth outreach, HIV/AIDS prevention) [77, 86–88].

*(3) A disproportionate amount of health facility resources are required to provide post-abortion care*. Studies agreed that the costs of post-abortion care were a burden to health systems, requiring a disproportionate amount of health facility resources and stretching overstretched health system resources [4, 38, 53, 89]. Some studies reported the use of physical resources for post-abortion care, such as up to 50% of hospital gynecological beds in Bangladesh or 25% of the main hospital’s blood supply before Guyana liberalized its abortion law [4]. Other studies reported financial costs of post-abortion care: more than seven times the Tanzanian Ministry of Health’s annual per capita budget [4] or more than half of facilities’ budgets for obstetrics and gynecology [53]. Estimates of the annual health system costs of post-abortion care in Africa and Latin America are considerable, ranging from US$ 159 million to US$ 476 million [38].

To reduce the number of patients seeking post-abortion care, it is cost effective to expand contraceptive services and supplies and to provide menstrual regulation and related care. Data from Nigeria show that if health systems provided contraceptive services and supplies to prevent unintended pregnancies that result in unsafe abortions, the cost-benefit ratio would be US$ 4 to US$ 1 when compared with the cost of post-abortion care [68]. In Bangladesh, menstrual regulation prevents unnecessary and expensive complications from unsafe abortions, with each case costing the health system a fraction (8–13%) of the cost of treatment for severe abortion complications [67].

*(4) Switching abortion techniques can have positive or negative economic impacts on health facilities*, *depending on the reason behind the changes*. Switching abortion techniques (e.g. performing abortion in ambulatory surgery centers, mandating multiple clinic visits) may result in negative economic impacts on health facilities when the changes result from laws designed to make abortion more expensive and difficult to provide [75, 85]. Other times, directives aimed at improving one aspect of abortion services, such as minimizing complications, may negatively impact a different aspect of abortion services. For example, by requiring abortion in Moldova to be performed by obstetrician-gynecologists in hospitals, hospitals had to centralize their services that resulted in higher costs of care and decreased client access to abortion [90].

Changes in abortion techniques can also facilitate financial savings and the expansion of abortion services. In Nepal, medical abortion has expanded access to first-trimester abortion in rural areas, since it does not require facilities to have capacity for surgical abortion [77]. In Sweden, a randomized-controlled equivalence trial found provision of medical abortion by nurse-midwives superior to provision by physicians: provision by nurse-midwives was more efficacious and less expensive [47]. In Malawi, manual vacuum aspiration delivered cheaper (i.e. financial and human resources), easier, and faster abortions (i.e. time to perform the abortion and time to discharge) than dilation and curettage [82]. In Latin America and the Caribbean, however, dilation and curettage produced similar outcomes to manual vacuum aspiration; a reduction in opportunity costs and an increase in service efficiency happened only after the reorganization of services [53].

*(5) Complex health systems impact the abortion-related trajectories of pregnant people*. Health system requirements and responses to new abortion methods impact the pathways of pregnant people. Where misoprostol is sold by prescription only in Latin America, pregnant people seeking the medication must pay for prescriptions, lie to providers about intended use, ask a third-party to obtain a prescription, or seek medication through unregulated channels [91]. In Brazil, inflated commercial values of misoprostol and price fluctuations dependent on facility type encourage non-facility-based procurement with reported prices as high as US$ 144 despite the mean cost of misoprostol being US$ 6 [92]. A study of Caribbean states found that hospital-based gynecologists sought to control the provision of Cytotec (misoprostol), whilst abortion providers admitted that the cost of abortions led them to recommend pregnant people buy medications from pharmacies and return to them in case of complications [93]. Permitting conscientious objection in Mexico has led to suggestions that provider objections were based on financial incentives, with providers objecting in public facilities but providing abortion care in their private facilities [94]. Where health systems regulations interfere with access to abortion, pregnant people face more complex pathways to obtaining care, including pathways that might increase potential associated risks like seeking medical abortion from unlicensed providers.

In reviewing data on studies that reported economic impacts of abortion at the mesoeconomic level, economic impacts of abortion on communities are rarely studied. The paucity of evidence on this topic does not align with the frequency that abortions occur in communities or the potential economic impact of unsafe abortions on communities. Two studies (2/150) reported the economic impact of abortion on communities, broadly speaking. In New Brunswick, Canada, a regulation from 2015 insufficiently mitigated challenges resulting from the province’s refusal to fund abortion care in health clinics [95]. Researchers found that even if the province completely eliminated the two-physician requirement, the resulting impact on access to affordable, timely abortion care would be marginal [95]. In India, evidence from a study on prenatal sex selection found that regions with an increasing male-female sex ratio experienced an increase in families’ parental education, an increase in maternal age at first birth, and a decrease in the likelihood of rural residence [96].

### Benefits and value of abortion provision and policies

Twenty-seven studies reported economic benefits (n = 17) or value (n = 11) of abortion at the mesoeconomic level (S3 Table); one study reported both benefits and value, so the number of data points exceeds the number of studies. Across the studies, two themes emerged: (1) the benefits of abortion differ by method; and (2) health systems often, but not always, respond to women’s needs around abortion.

*(1) The benefits of abortion differ by method*. Multiple studies compare the benefits of surgical abortion methods to medical abortion methods. The health system benefits, often reported in terms of cost and efficacy, vary depending on where the study occurred [45]. In Nigeria, manual vacuum aspiration in clinics was the least expensive and most effective option relative to unsafe abortion, whereas in Ghana, medical abortion was the most cost-effective option relative to unsafe abortion [62]. In the United Kingdom, replacing surgical abortion procedures in the operating room with manual vacuum aspiration resulted in an annual savings of GB£ 60,000 [65]. In China, 33.1% of rural abortion providers and 38.1% of urban abortion providers reported that an advantage of medical abortion is that it is less expensive than surgical abortion, and medical abortion is also a low-risk alternative without surgery and anesthesia [97].

Within-method benefits can be further enhanced by task shifting, service reorganization, and streamlining. In Sweden, task shifting of medical abortion from physicians to nurse-midwives produced direct economic benefits: shorter time providing care, lower salaries among nurse-midwives than physicians, shorter waiting times among patients, and a potentially lower cost to treat complications [47]. Reorganizing services by treating clinically eligible patients in the manual vacuum aspiration unit rather than the main operating room could free up needed hospital beds by shortening patients’ stays and freeing up physicians and anesthesiologists for other cases [98]. Streamlining the protocol for medical abortion to two or three clinic visits rather than four visits, as required in Mexico City in 2005, would reduce the costs of medical abortion without affecting patient safety [64].

Of course, providing safe abortion instead of unsafe abortion is the most important factor to improve health outcomes and economic outcomes [29, 62]. Reducing unsafe abortion by increasing access to safe abortion can reduce the costs of care and benefit health systems. In two public referral hospitals in Ouagadougou, Burkina Faso, the hospitals would have saved over US$ 19,778 in 2010, had safe abortion care services been available [5]. In Mexico City in 2005, a modest increase in access to manual vacuum aspiration would improve access to safe abortion and result in savings of over US$ 50,000 among the three public hospitals and one private clinic being studied [29].

*(2) Health systems often*, *but not always*, *respond to women’s needs around abortion*. In settings where policies and laws do not restrict discussions about abortion to clients and providers in facilities providing abortion, a range of health providers can respond to women’s needs for pregnancy options counseling and referrals to abortion clinics [76]. By offering medical abortion patients alternative forms of follow-up care (e.g. phone calls, internet communication), health systems may support patients’ preferences and needs while simultaneously experiencing potential cost-savings resulting from patients being less likely to seek follow up care from emergency departments [99]. In Mexico, liberalizing abortion laws to permit abortion due to rape resulted in a statistically significant increase in women accessing induced abortion in the public sector [100]. Responding to women’s needs around abortion includes expanding access to health insurance that includes abortion coverage. In the United States, state Medicaid coverage of medically necessary abortion reduced the risk of severe maternal morbidity by an average of 16% [101].

At times, the health system fails to meet women’s needs around abortion. Access barriers may prevent women from obtaining legal abortions [102]. Existing abortion services may be inappropriate or even harmful, lack appropriate referrals, and deliver limited knowledge on contraceptive methods to post-abortion patients [103]. Providers’ values may result in a double discourse around abortion. Providers who consciously object to providing abortion in the public sector may willingly overlook these public values to perform abortions in the more profitable private sector [26].

## Discussion

By systematically scoping the global evidence for the first time across the mesoeconomic domain, this article establishes the substantive understandings and methodological approaches that have been used to understand the economics of abortion-related care and policies within health systems and communities. While this scoping review provides a rich source of evidence on mesoeconomic outcomes globally, the variation with which researchers identify the components of abortion services and cost abortion services makes it challenging to provide conclusive figures on the average costs of abortion services across settings. Economic impacts of abortion at the mesoeconomic level are less frequently studied, especially the impact on communities. The paucity of evidence on this topic does not align with the frequency that abortions occur in communities. Although the mesoeconomic costs of post-abortion care and treating complications from unsafe abortion are somewhat evidenced in the formal health sector, we know little about the economic factors influencing the delivery of unsafe abortion services by the informal health sector.

This scoping review was conducted as part of a larger study on the economic costs of abortion. By design, we excluded grey literature (such as in-service reports and book chapters) published outside of journals and literature published in languages other than Dutch, English, French, German, and Spanish. The searches and screening were conducted without regard to economic level (microeconomic, mesoeconomic, and macroeconomic); economic level was determined during data extraction. Due to the large number of studies generated by the searches, eight researchers (SRL, EC, YR, BM, CP, EZ, JS, and LG) were involved in the screening and data extraction stages. While definitions for our economic outcomes (i.e. costs, impacts, benefits, and value) and quality processes were established in advance to ensure consistency in screening, application of the inclusion/exclusion criteria, and data extraction [17], identifying relevant items depended on each researcher’s ability to overcome remaining challenges in identifying studies for inclusion based on the available text. The research team followed a standardized protocol and continuously attempted to address these challenges throughout the process, but any shortcomings remain a limitation of this scoping review. To help minimize these limitations, JS conducted robustness checks and SL reviewed all extracted data for quality assurance.

## Conclusions

Much remains to be learned, yet key themes in abortion care services and abortion policies are remarkably consistent across geographies. A disproportionate amount of health facility resources are required to provide post-abortion care. Economic benefits differ by abortion method/technique. Adapting to changes in laws and policies is costly for health facilities. Limited resources negatively affect health facilities’ ability to meet client demand and to provide quality abortion care services. Financial savings can be realized while maintaining or even improving quality of abortion care services.

Since this scoping review is focused on the economics of abortion, we have limited our recommendations to those relating to the economics of abortion. Whilst topics like training, methods, barriers to safe provision, and the number of abortions performed are important, they are beyond the scope of this manuscript. In order to better address the economic costs and impacts of abortions on health systems, health facilities, and communities, the following recommendations are made for future research and reviews:

- Methodologically, review papers are the most frequent type of study at the mesoeconomic level, suggesting that researchers rely on the same body of evidence from a core set of costing papers. This finding raises questions about the timeliness and updating of these costing papers. Studies generating new primary data on mesoeconomic outcomes are needed to strengthen the evidence base.
- We uncovered no evidence on the mesoeconomic costs, impacts, benefits, or value of abortion in the Middle East and North Africa. Greater research is warranted in these regions.
- The paucity of evidence on the economics of abortion within communities does not align with the frequency that abortions occur in communities or the potential economic impact of unsafe abortions on communities.
- The reporting of costs should always include the year and specific currency used (e.g. ‘dollar’ could refer to international dollar, Australian dollar, Canadian dollar, or American dollar). Authors should clarify whether the value presented refers to the time of publication or the time the research was conducted. This level of specificity is required and needs to be reported consistently in order to prevent inaccurate assumptions and permit researchers to accurately compare data across studies.
- Whilst the mesoeconomic costs of post-abortion care and treating complications from unsafe abortion are somewhat evidenced in the formal health sector, it would be helpful to know more about how the economic factors influencing the delivery of unsafe abortion services by the informal health sector, including how providers of unsafe abortion establish their pricing systems.

## Supporting information

S1 Table Summary table of included studies reporting mesoeconomic costs. This table summarizes all studies reporting mesoeconomic costs (n = 116).
(PDF)

S2 Table Summary table of included studies reporting mesoeconomic impacts. This table summarizes all studies reporting mesoeconomic impacts (n = 40).
(PDF)

S3 Table Summary table of included studies reporting mesoeconomic benefits and/or value. This table summarizes all studies reporting mesoeconomic benefits and/or value (n = 27).
(PDF)

S1 File Preferred Reporting Items for Systematic reviews and Meta-Analyses extension for Scoping Reviews (PRISMA-ScR) checklist. (PDF)
