# Supplementary material for: The mesoeconomics of abortion: A scoping review and analysis of the economic effects of abortion on health systems
Source: PLoS One. 2020 Nov 4;15(11):e0237227. doi: 10.1371/journal.pone.0237227 (PMC7641432; doi:10.1371/journal.pone.0237227)
Supplement: S1 Table — This table summarizes all studies reporting mesoeconomic costs (n = 116). (PDF) [file pone.0237227.s001.pdf]

S1 Table. Summary table of included studies reporting mesoeconomic costs (n=116)

Author, year [country]	Aim/objective(s)	Population	Study type	Summary of main findings
(Aantjes, Gilmoor et al. 2018) [eastern and southern Africa]	To identify and synthesize the literature on postabortion care (PAC) in eastern and southern Africa with the aim of reporting on the reach, quality, and costs of these services	Varied by studies on PAC, health workers, and abortion-related service reviews	Systematic review	Medical abortion (MA) is the cheapest method followed by manual vacuum aspiration (MVA) and then dilatation and curettage (D&C).<br><br>In Malawi in the absence of complications, researchers estimated MA to cost U$ 12, MVA to cost U$ 19, and D&C to cost U$ 19. In the presence of complications, PAC costs increased to U$ 63 for severe non-surgical complications and U$ 128 for severe surgical complications.<br><br>In South Africa, researchers estimated MA to cost U$ 61 and MVA to cost U$ 69. In Swaziland, MA cost U$ 75 and D&C cost U$ 115. In Rwanda, PAC costs were higher if treated in regional health facilities (U$ 239) than in district hospitals (U$ 93) or health centres (U$ 72).<br><br>Treatment costs for one instance of post-abortion morbidity represented more than three times the annual per capita health expenditure in Uganda and more than five times that in Ethiopia. Law reform and provision of safe abortion could cut this expenditure by 2-2.5 times.
(Acosta de Hart, Umaña et al. 2002) [Colombia]	To descriptively review Orientame's service over 25 years	People living in Bogota that are seeking care	Narrative description (historical)	Clinics offer a sliding scale of fees for services, so that the 40% of patients with higher incomes subsidize the 60% with lower incomes. This practice allows the clinics to be self-supporting. When there has been more income than costs, the money has been invested in community-based programs in the slum areas of Bogota.
(Afable-Munsuz, Gould et al. 2007) [United States]	To: 1) describe the range of practice models, in terms of staff mix and number of visits, used to provide medication abortion in 11 abortion care settings across the United States; 2) document financial costs, including staff salary costs, to providers of medication abortion in the 11 abortion care settings; and 3) examine whether variation in practice models is associated with	Abortion-seeking patients, facility staff and managers, administrative facility documents, and medical chart at 11 sites across the United States	Mixed methods	Total cost per episode includes direct staff costs (clinical and nonclinical costs), space costs, and indirect costs. The mean total episode cost across sites was $346 (range $252-460). Costs for medication ranged from $80 to $102. Clinical staff costs were highest for medical doctors (mean of $38). The mean cost for nurse practitioners/physician's assistants/sonographers ranged from $12-52. The lowest mean cost ($19) was for medical assistants/social workers/registered nurses. Total staff time with each patient ranged from 37-103 min. Total staff costs ranged from $27-122. Sites with a mix of nurse practitioners and medical assistants were able to provide more staff time with the patient while maintaining moderately low clinical staff costs.

Author, year [country]	Aim/objective(s)	Population	Study type	Summary of main findings
	variation in cost and/or patient satisfaction			
(Anjum 2000) [United Kingdom]	To assess the efficacy, practicality, and cost-effectiveness in clinical practice	All women presenting for medical termination of pregnancy before 63 days of gestation at the Northern General Hospital in Sheffield (n=369 terminations)	Clinical audit	Reducing the dose of mifepristone from 600mg (costing £41.83) to 200mg could save £28.60. Using misoprostol as part of the treatment regimen cost £48 and was significantly cheaper than gemeprost.
(Ayanore, Pavlova et al. 2017) [Ghana]	To explore what direction women perceive gender roles to influence their reproductive choices and care	Women with records of recent birth (≤ two years prior to study) (n=90), health staff (n=16), and policymakers (n=6)	Qualitative ethnographic study using focus groups and in-depth interviews	At the community, facility, and district facility levels, PAC services are neither provided nor covered under the antenatal/postnatal fee exemption policy of the national health insurance scheme. To account for these charges, staff members asked women to pay (US$ 25) for care.
(Babigumira, Stergachis et al. 2011) [Uganda]	To perform a comprehensive assessment of the economic burden of induced abortion in Uganda in terms of its costs	Databases on abortion incidence and cost throughout Uganda	Cost analysis	The national annual expenditure on induced abortion is projected to be $23.6 million in direct medical costs and $7.0 million in direct non-medical costs. Most healthcare costs can be attributed to the treatment of the complications of unsafe abortions. Per case, the average direct medical cost was $65 (range $49–$86), and the average direct non-medical cost was $19 (range $16–$23).
(Baird 2015) [Australia]	To consider the context through which MA has become available in Australia since 2013	Narrative review of the introductive of MA in Australia	Mixed methods: Historical analysis complemented by expert interviews	In addition to the cost of the MA drugs, there are medical practitioner consultation fees, ultrasound costs, and blood tests or other tests. Insurance companies that provide medical indemnity initially considered the risks of providing MA to be the same as providing surgical abortions. Doctors who may have considered incorporating MA into their practice were thus faced with a prohibitive annual increase in their medical indemnity insurance. After this disparity was remedied at the end of 2014, one doctor hope that the extensions of the authorised use of mifepristone would ease the time pressure and enable more general practitioners to incorporate MA into their practice. In Victoria, the uptake of MA in rural settings was supported by offering information and training sessions; as a result, two additional general practices provide MA and only charge the patient the cost of the medication. An Australia-wide telephone consultation home medical termination of pregnancy service has been developed to reach women in rural areas with limited access to abortion services.
(Baird 2017) [Australia]	To argue that while decriminalization is consistent with feminist goals and human	Documentary evidence in four Australian jurisdictions and interviews with experts	Literature review and expert interviews	In the early 2000s, the clinic had about ten lawsuits against it. Plaintiffs alleged various forms of poor practice, most in relation to abortion. Most women were supported by Catholic anti-choice agencies. Then

Author, year [country]	Aim/objective(s)	Population	Study type	Summary of main findings
	rights principles, unless there have been specific legal restrictions on abortion provision beyond defining the legality of doctors' authority to decide, it will make little or no challenge to the sources of inadequate access to abortion in Australia			the HIH Insurance company went into provisional liquidation. The abortion clinic and its doctors were forced into more expensive insurance arrangements. Whilst the clinic was committed to means-tested fees and "payment plans," the opportunity for women to pay for their abortion over time became financially unviable.
(Battistelli, Magnusson et al. 2018) [United States]	To improve understanding of the organizational facilitators of and barriers to the provision of abortion care by nurse practitioners, certified nurse midwives and physicians' assistants. Specifically, our qualitative analyses examined how organizations participating in Health Workforce Pilot Projects implemented changes to their provision of abortion care following the law's implementation in California	20 administrators from five organizations based in metropolitan areas that operate abortion clinics	Qualitative: interviews	Shifting services among provider groups had logistical and financial costs. Participating organizations were tasked with moving nurse practitioner, certified nurse midwife and physicians' assistant abortion provision from the research environment to every-day clinical practice. Interviewees described interrelated disincentives to instituting these changes that could be broadly categorized as organizational reliance on physicians, reduced demand for abortion services and state Medicaid reimbursement rates. The shifting of clinician roles can require changes in clinical operations and staffing that sometimes cost an organization more, at least in the short term. Several respondents cited the state's low Medicaid reimbursement rate for abortion services as a key driver of the maldistribution of care. Some organizations consolidated abortion services into a few high-volume clinics to be economically sustainable.
(Baxerres, Boko et al. 2018) [Benin and Burkina Faso]	To document the means women use to obtain abortions in the capital cities of Benin and Burkina Faso<br><br>To learn whether or not use of misoprostol has become an alternative to other methods of abortion and the implications for future practice	Women in Cotonou (n=21) and in Ouagadougou (n=13), including 5 secondary school students	Qualitative in-depth interviews	In Burkina Faso, health centres used curettage, MVA, and injections to induce abortions. Fees were recorded as 30,000 CFA (US$ 48.50) in public health centres and 60,000 CFA (US$ 97) in private clinics. In Benin, the general consultation fee at of the few private clinics in Cotonou was expensive (upwards of 10,000 CFA or US$ 16.18). Small private health centres, found in the southern part of the country, charge comparable prices (between 15,000-55,000 CFA or US$ 24.50-89) to public health clinics.
(Belton and Whittaker 2007) [Thailand]	To examine the need for provision of reproductive services to displaced populations, specifically Burmese migrant workers along the border with Thailand	Burmese women with postabortion complications (n=43), lay midwives (n=15), health workers (n=20), male partners of women with postabortion complications	Mixed-methods ethnographic study involving a retrospective review of medical records, directed group	The cost of 116 days of PAC in the hospital for 31 women was 71,432 Baht (US$ 1,748). Mae Tao clinic paid the costs of cases they referred to the Thai hospital; self-referred undocumented migrant workers paid for their own care. Misoprostol, reportedly used in the past, no longer appeared to be available in Mae Sot. Induced abortions conducted by trained medical staff can be obtained at private clinics in regional

Author, year [country]	Aim/objective(s)	Population	Study type	Summary of main findings
		(n=10), and community members	discussions, semi-structured interviews, and informal conversations	towns. Fees ranged between 3,000 and 7,500 Baht (US$ 70-174) depending on the service and gestation of the pregnancy. Their illegality forces the prices up, and little follow-up care exists.
(Benson, Nicholson et al. 1996) [Sub-Saharan Africa]	To document the magnitude of abortion complications in Commonwealth member countries and document the research gaps	Literature on women seeking unsafe abortion services in sub-Saharan Africa	Literature review	The study in Kenya found that the average length of stay was shorter for patients treated with MVA versus sharp curettage (SC); the reduced patient stay resulted in lower hospital costs. The cost of treating incomplete abortion patients with MVA was much less than that associated with SC. The average cost per SC patient in one district hospital was US$ 15.25; MVA decreased the cost by 66%. These reductions reflected decreases in the amount of resources used (e.g. staff time, bed space, pain medication). A study in Nigeria found that average treatment costs for a septic abortion patient were US$ 223.11. It cost the hospital an average of US$ 7.50 per patient to treat abortion complications compared to an annual Ministry of Health per capita budget of US$ 1.00.
(Benson, Okoh et al. 2012) [Nigeria]	To: (1) describe current PAC caseloads and treatment regimens in selected study public hospitals; (2) calculate estimates of the per-case costs of treatment of abortion complications; and (3) calculate estimates of annual costs of treatment of abortion complications in all public hospitals in the three study states	PAC-providing public hospitals in Ogun, Lagos, and Abuja (n=17)	Survey/data collection tool	PAC treatment with moderate complications cost an average of US$ 112 per case, 60% more than a simple PAC case (US$ 70). Treating a PAC case with severe complications (US$ 258) was >3.5 times the cost of treating a simple PAC case. Estimated per-case costs of each procedure varied widely across facilities: MVA/electric vacuum aspiration (EVA) (US$ 43 to US$ 141); D&C/dilation and evacuation (D&E) (US$ 44 to US$ 114); misoprostol alone (US$ 48 to US$ 129); and expectant management (US$ 32 to US$ 104). Overall average per-case cost of inpatient PAC treatment (US$ 95) was 38% more expensive than outpatient treatment (US$ 69). Reliance on physician providers contributed to overall higher costs.<br><br>For the 17 study facilities, the total annual cost under current treatment conditions is US$ 274,015. All 79 PAC-providing public hospitals in the 3 included Nigeria states are estimated to spend US$ 807,442 on PAC provision annually.
(Benson, Gebreselassie et al. 2015) [Malawi]	To estimate current health system costs of treating unsafe abortion complications and compare these findings with newly-projected costs for providing safe abortion in Malawi	Malawi's health system	Estimation study based on survey and costing data	The median per-case costs for labour and supply inputs for simple uterine evacuation (UE) were approximately $7 each. Within the study facilities, the estimated median per-case cost of treatment for all PAC cases was $40. The median cost per D&C case ($63) was 29% higher than an MVA case ($49). The median cost of care for a simple PAC case with D&C ($19) was 46% higher than with MVA ($13) and 58% higher

Author, year [country]	Aim/objective(s)	Population	Study type	Summary of main findings
				than misoprostol ($12). The estimated median per-case cost also varied by treatment category. Treatment of a case with severe non-surgical complications ($63) was almost five times higher than that of a simple PAC case ($13). Although few in number, cases with severe surgical complications were especially costly to treat ($128), almost 10 times higher than a simple case. Even within the same UE procedure type, the more severe the complications, the higher the estimated per-case cost. For women treated with MVA, the cost of a severe non-surgical case was almost five times higher than that of a simple case.
(Berer 2000) [global]	To examine the changes in policy and health service provision required to make abortions safe	Wide-ranging review of published and unpublished sources	Literature review	Where abortion is clandestine and unsafe, public health services often pay for the treatment of complications in tertiary level hospitals, where costs are highest. Covering the cost of safe abortions in public health services shifts expenditure away from complicated cases in tertiary level hospitals to safe, simple procedures that can be provided in primary clinics. A Tanzanian study estimated that the cost per day of treating abortion complications, including the costs of drugs, meals, staying costs, and surgical procedures, was more than seven times the Ministry of Health's annual per capita budget.
(Berer 2005) [global]	To discuss choice and acceptability of MA from the perspective of both women and abortion providers and argues that choice of method is important for both	Review of recent papers and information presented at a conference on MA	Review	A study on the cost of MA in the public health system in South Africa found that one of the main cost drivers is the dosage of mifepristone (200 vs. 600mg). While 200mg is just as effective, many countries approved mifepristone with a regimen of 600mg before this information was available. The change to 200mg has been slow due to the high price of mifepristone, type of provider involved, whether the clinic was in a primary or secondary level facility, and the currency exchange rate affecting imported drugs. Identifying the main cost drivers shows how costs might be reduced without reducing quality of care (e.g. locally produced, generic mifepristone and misoprostol, use of a 200mg dose of mifepristone, provision in primary care clinics with mid-level providers up to nine weeks and mid-level providers in secondary level facilities after nine weeks, with back-up from obstetrician-gynaecologists). A study in India showed that neither hospital beds, operating theatre or ultrasound were required for MA, which is important for cost reduction. Unless the costs of surgical and MA are broadly similar, many women, health providers, and health insurance companies will feel they need to choose the cheaper option that limits choice.
(Betala Belinga,	To measure the actual cost of	Abortion procedures that	Evaluation of	Based on the cost evaluation of 528 surgical abortions with

Author, year [country]	Aim/objective(s)	Population	Study type	Summary of main findings
Valence et al. 2010) [France]	abortion within a public health facility by comparing it to the cost of similar care: an ended pregnancy (spontaneous miscarriage) requiring ambulatory care	were valued/costed and invoiced by the regional maternity unit in the regional maternity hospital in Nancy during 2008 (n=528)	revenue/cost data	anaesthesia, the cost per abortion is € 562. This cost includes the direct cost of service (€ 212), the indirect block charge (€ 110), the indirect block anaesthesia load (€ 80), and the cost of hotel and logistics (€ 160). After applying the tariffs, the cost to the facility is reduced to € 180. This results in a loss varying between € 66,660 and € 87,497 for the management of surgical abortions.
(Billings and Benson 2005) [Latin America and Caribbean]	To review results from 10 major PAC operations research projects conducted in public sector hospitals in seven Latin American countries, completed and published between 1991 and 2002	Review of results of 10 PAC operation research projects in seven countries	Review	Significant improvements in PAC services can be made in public sector hospitals while simultaneously reducing the cost of service delivery. In two study sites, the UE procedure was moved from the operating theatre to an obstetrics-gynaecology ambulatory care area, resulting in reduced costs and average length of patient stay (ALOS). Treating women with incomplete abortion can absorb more than 50% of facilities' obstetric and gynaecologic budgets. The high cost of services may be a barrier to obtaining clinical services. Switching to MVA while reorganizing services to an outpatient basis tends to substantially reduce ALOS and treatment costs to the facility. In some cases, administrators passed these savings on to the patients by reducing fees. As a result of these changes, one hospital was recovering almost 98% of the full cost of providing PAC services at the 2000 follow-up compared with 45% of the cost prior to the intervention.
(Brown and Jewell 1996) [United States]	To estimate directly the responsiveness of abortion demand to county-level variations in travel-cost component of the full cost of abortion services	Abortion providers in Texas, health facility data	Log-linear regressions	Counties with abortion providers have higher time costs that result from higher median household incomes.
(Cano and Foster 2016) [Canada]	To document women's experiences seeking and obtaining abortion services while residing in Yukon Territory, identify financial and personal costs, and explore avenues through which services could be improved	Women who accessed abortion services on/after January 1, 2005, while residing in the Yukon (n=16)	Qualitative study involving semi-structured in-depth interviews	All of the procedures through Whitehorse General Hospital were covered by Yukon's territorial health insurance.
(Chełstowska 2011) [Poland]	To examine the economic consequences of the stigmatisation and illegality of abortion and its almost complete removal from public health services in Poland since the late	Polish women	Mixed methods review of data from government documents, reports of non-governmental and international	In the private sector, a vast new, profitable market in health care emerged without any government control on price, quality of care, or accountability. If the cost of a surgical abortion is on average 2,000 PLN, and an estimated 150,000 procedures are carried out annually, that would make about 300 million PLN of annual income (approximately US$ 95 million or € 75 million) unregistered and tax-

Author, year [country]	Aim/objective(s)	Population	Study type	Summary of main findings
	1980s		organisations, and published articles	free. Neither the real number of procedures, nor the exact cost is known since the private sector is outside government control.
(Choobun, Khanuengkitkong et al. 2012) [Thailand]	To compare the hospital charges, duration of in-hospital procedures, clinical course and complications between MVA and SC	Women undergoing first-trimester abortions at the Songklanagarind Hospital (n=80)	Prospective observational study	The total hospital cost for MVA was significantly lower than that for SC, roughly a 64 % reduction in the total expenditure (US$ 54.67 vs. US$ 153.97). The most substantial costs were related to equipment and laboratory investigations, medication, and service costs; all were significantly less in proportion among MVA patients than the SC ones.
(Coast and Murray 2016) [Zambia]	To analyse the details of pathways to care, barriers and delays, and the role of others in influencing these pathways in order to develop knowledge and theorisation on abortion care-seeking behaviour and its influences	Adolescents and women aged 15-43 years (n=112) presenting for surgical abortion or PAC at an emergency ward in the Maternity Department at University Teaching Hospital in Lusaka	In-depth qualitative interviews	One participant said the 'doctor' (who may have been a doctor or a clinical officer) had asked her for K200 (£ 24.00) to go through with her treatment. This was a demand for an under-the-counter payment, not a hospital registration fee. Illegal, unofficial provider payments are quite frequently expected and paid in the formal health sector, and are an open secret. Abortion stigma gives providers leverage to extort such fees, knowing that women are desperate and unlikely to expose these financial demands for fear of revealing an abortion. When unofficial payments are paid, secrecy means that there can be substantial gaps between expected and actual costs. Some medical personnel in the public sector's gynaecology services also provided abortion-related care services at those facilities under private arrangements. The hospital had a private ward and differential fee scales; there was also a difficult-to-quantify undercurrent of unofficial private practice that exploited the stigma surrounding abortion. Some providers charged high fees to women (up to 400 Kwacha or £ 48), far beyond the amounts set by the hospital (around 10—100 kwacha or £ 1.20—12 for standard procedures). Herbalists will extend a line of credit for clients, often with deferred payment until treatment has been successful.
(Cockrill and Weitz 2010) [United States]	To explore abortion patients' perspectives on abortion regulations	Abortion patients (n=20) at the only three high-volume abortion facilities in two states in the South and Midwest	Qualitative study using semi-structured interviews	Of the ten women who were insured, only two women attempted to get coverage for their abortion. The remaining women did not attempt to get coverage because they already suspected their insurance would deny them coverage or because they did not want their insurance provider, employer, or parents to know about their abortion.
(Colman and Joyce 2011) [United States]	To examine the impact of the Women's Right to Know Act in Texas (disclosure, waiting period, and surgical centre regulations) on number, timing, and cost of abortions and distance travelled	Reported data for abortions performed in Texas	Regression analysis	The charges for an abortion at 20 weeks' gestation increased about 37 % (or about $454) more in Texas between 2001 and 2006 relative to the other states. There was no relative increase in charges at 10 weeks' gestation. Recent state policies toward abortion have been targeted at the provision of abortion services. According to the major federation of abortion providers, these so-called targeted regulation of abortion provider laws can greatly increase the costs of providing abortion

Author, year [country]	Aim/objective(s)	Population	Study type	Summary of main findings
				services. Arguably the most costly of these provisions is that abortions of a specified gestation be performed in an ambulatory surgical centre.
(Contreras, van Dijk et al. 2011) [Mexico]	To examine the experiences and opinions of health care professionals after the legalization of abortion in Mexico City in 2007	64 semi-structured interviews with obstetricians/gynaecologists, nurses, social workers, key decision makers at the Ministry of Health, and others	Qualitative study using semi-structured interviews	Over one-third of health care professionals (38%) were opposed to abortion services being provided free of charge, feeling that fees should be in place to recover procedure costs and to act as a deterrent of 'irresponsible' behaviour and repeat abortions.
(Costa 1998) [Brazil]	To examine women's experience with misoprostol and its impact on women's health	Women admitted to hospitals for abortions/PAC in Rio de Janeiro	Review	The mean cost of misoprostol was US$ 6. In "clandestine clinics," the average abortion cost US$ 144 USD and catheter insertion cost US$ 42. Access to misoprostol was easier in smaller facilities than in larger ones. Inflated commercial value of misoprostol has led to reports that it is now being sold by unlicensed providers in the favelas of Rio de Janeiro and Sao Paulo.
(Couteau, D'Ercole et al. 2016) [France]	To propose a protocol for induction of labour to terminate pregnancy after 22 weeks of amenorrhea allowing to decrease the duration of labour and of hospitalization but also, allowing to reduce the number of emergency pretreatment-induced fetal death, to improve the experience of the patients and to limit the cost	Patients receiving medical termination of pregnancy (TOP) at 22 gestational weeks and beyond, in a maternity hospital, the North Hospital of Marseille (n=269)	Retrospective single-centre study	For TOP over 22 gestational weeks, the hospital receives € 1,996. The tariff remains the same for a period of hospitalization of 1 to 8 days. A day of hospitalization in the service of high-risk pregnancies and laminaria procedure cost maternity € 348 euros. The protocol put in place thus allows a saving of € 348 by termination of pregnancy.
(Creinin 2000) [United States]	To examine in a randomized trial the clinical efficacy and patient acceptance of medical abortion using oral methotrexate and vaginal misoprostol compared to surgical manual vacuum aspiration in women with pregnancies up to 49 days' gestation	Women (n=50) who were 18 years of age or older, requested an elective abortion, had a singleton intrauterine pregnancy not exceeding 49 days' gestation documented by vaginal ultrasound, were willing to be randomized to either medical or surgical abortion, were willing to avoid intercourse for the first two	Randomized trial [with no control]	Based on a physician's hourly wage being three times that of a physician assistant and eight times that of a research assistant/counsellor, the study estimated that surgical abortion would be 10% more expensive than medical abortion. However, where the physician's hourly wage is two times that of a physician assistant, then surgical and medical abortion costs would be identical.

Author, year [country]	Aim/objective(s)	Population	Study type	Summary of main findings
		weeks of study participation, had adequate venous access for multiple phlebotomies, had access to a telephone, and were willing to have a suction aspiration if randomized to medical abortion and surgical intervention was indicated		
(Creinin, Shore et al. 2005) [United States and India]	To evaluate relative differences in direct and total (direct and indirect) costs for MA regimens mifepristone and misoprostol or misoprostol alone	Modelled cost data	Modelled cost data	Although mifepristone costs US$ 83.33 for every 200-mg tablet in the United States, the actual excess cost of using a mifepristone regimen, as compared with a misoprostol-alone regimen, is only US$ 22 to US$ 32. The actual cost of a mifepristone regimen is lower than that of a misoprostol-alone regimen in India. In a hypothetical developing country, a mifepristone regimen is likely to be less expensive than regimens using misoprostol alone. Because of the higher efficacy of MA regimens using mifepristone and misoprostol and the need for fewer follow-up evaluations, such regimens are less expensive or only minimally more expensive than those using misoprostol alone.
(Crighton and Ebert 2002) [Europe; France; Germany]	To explore the impact of this controversial technology on abortion rates and practices in Europe, looking first at the EU region as a whole, then examining more closely the politics of RU 486 in France and Germany	Abortion rates, policies, and practices for each nation state	Policy analysis from published literature	Availability of services varies from clinic to clinic in many countries because physicians may choose for personal or economic reasons not to offer MA. For example, Germany and Great Britain limit access to Mifegyne indirectly because their public health systems do not cover physicians' treatment or facilities costs. Women who have just been given a prostaglandin are required to stay in the clinic for half a day or more as they wait to miscarry, so psychological support and a reasonably comfortable waiting room are minimum requirements. Since these services do not earn "performance points" under National Health Service (NHS) guidelines, clinics have little reason to introduce them. Another study listed two more disincentives for potential MA providers: the special licenses required for clinics offering these services and the high purchasing price of Mifegyne and associated prostaglandins. For medical providers, the cost of drugs and services associated with the Mifegyne+PG procedure renders surgical abortions more economic. The high cost to clinics of MA drugs and the low payment rates set by health ministries for MA providers are part of the explanation for access problems in both Germany and Great Britain.
(Dawson, Bateson et	To identify quality studies of	United Kingdom (n=14),	Systematic review	A study in an English primary health care trust noted that the cost of a

Author, year [country]	Aim/objective(s)	Population	Study type	Summary of main findings
al. 2016) [United Kingdom; United States; Russia; Australia; New Zealand; Canada]	abortion services to provide insight into how access to services can be improved in Australia	United States (n=7), Russia (n=2), Australia (n=2), New Zealand (n=3), Canada (n=1)		medical TOP or a surgical TOP (day case) ranged from £462 to £578 per patient. Sharma et al.’s study of a pilot local anaesthetic outpatient surgical TOP service found that the cost could be reduced to £366 per patient. However, if this involved outpatient consultation, the cost was £217 per patient; this cost could be further reduced to £177 if the nurse telephone clinic was used. The nurse telephone clinic reduced the time needed by the doctor to assess each patient, increasing the number of patients that could be seen per clinic. Research in the United Kingdom also examining a local anaesthetic outpatient surgical TOP service found that a cost savings was made of approximately £60,000 per year and that the operating theatre use was reduced by one termination list per week.
(Dennis, Manski et al. 2014) [United States]	To answer the following questions: (1) What do women know about the cost of abortion and the availability of Medicaid coverage for abortion? (2) Where do women obtain this information? (3) What are women’s experiences paying for care?	Low-income women over the age of 18 who had an abortion within the past two years within one of the four study states (n=98)	Qualitative study: semi-structured interview	State-level differences existed in the information given to women by staff at abortion facilities. Women in Arizona and Florida said they were informed that the state Medicaid program does not cover abortion or that it would be extraordinarily difficult to obtain coverage. Women in New York, Oregon, and Massachusetts were almost universally informed that coverage was available. Women reported that staff at abortion facilities directed them to enrol in Medicaid, suggesting that these staff and facilities are an underutilized resource for helping pregnant women enrol in public or subsidized insurance.
(Díaz-Olavarrieta, Cravioto et al. 2012) [Mexico]	To identify the perceptions and opinions of people who provide abortion services in Mexico City, three years after implementation of elective abortion legal reforms	Health workers assigned to the legal abortion programs at a clinic (n=10) and a hospital (n=9) in Mexico's Federal District	Qualitative: interviews	Participants associated the exclusivity of the law in the Federal District with super-saturation of services, due to the great demand of attention and insufficient material and human resources to satisfy the demand. Participants were in favour of the rest of Mexico replicating the legal reforms, as it would prevent mobilization of women from other states that is expensive and increases the workload.
(Dobie, Gober et al. 1998) [United States]	To survey 31 family planning clinic sites in rural Washington State about their sponsorship, staffing, service provision and population coverage	Family planning clinics in rural Washington (n=31)	Cross-sectional survey	The most commonly mentioned barrier to providing additional services for patients was cost. Of the clinics surveyed, only one provided abortions on-site. Federal funding of most of these sites prohibits on-site pregnancy terminations.
(Doran and Hornibrook 2014) [Australia]	To identify factors that New South Wales (NSW) rural women experience in relation to their ability to access an abortion service and follow-up care	Rural clinics in NSW (n=7) and women who sought abortion services at those clinics (n=13)	Qualitative: Surveys and interviews	Affordability and availability of services, correct referral process, continuity of pre- and PAC, and integration of services were seen as gaps in service provision. A shortage of doctors, especially women doctors, long waiting times, lengthy travel, and cost impacted on timely access to abortion and follow-up care.
(Duggal 2004) [India]	To examine the political economy	Literature/database review	Literature/database	A study of abortion providers in Delhi in 2002 found that the cost of

Author, year [country]	Aim/objective(s)	Population	Study type	Summary of main findings
	of abortion care in India by reviewing cost and expenditure patterns for abortion care in India		review	abortions varied considerably, depending on the number of weeks of pregnancy, the abortion method used, the woman's marital status, type of anaesthesia, whether acceptance of contraception was involved, whether it was a sex-selective abortion, whether any diagnostic tests (e.g. pregnancy test, sonography, laboratory tests) were carried out, medications given, location of the clinic, whether the provider was certified and the clinic registered, whether hospitalisation was required, and the nature of the competition. In the public sector, abortion services are usually free, but in recent years some states have introduced user fees or have allowed private practice by public providers. The clinic charges for an abortion ranged from Rs. 135–534 (average Rs. 370) for public providers and Rs. 394–649 (average Rs. 497) for private providers. Of these, the doctors got an average of 42%, 21% was spent on medicines, and the rest was used for hospital charges like operating theatre and bed charges.
(Dzuba, Winikoff et al. 2013) [Latin America and Caribbean)	To present evidence of MA's contributions to reduced complications, describe strategies to enhance safe MA, and highlight existing barriers to access in Latin America and Caribbean (LAC), while examining MA's role in newly legal abortion services	Literature on women seeking abortion services in LAC	Literature review	The estimated per-patient cost to treat postabortion complications in LAC is US$ 94.00. In 2009, most abortion services were shifted from hospitals to primary care clinics to facilitate access and improve quality of care. Due to primary use of medical methods, the Secretariat of Health of Mexico City (SSDF) has the capacity to attend to more than 40 women seeking abortions each day. Since mifepristone was registered in Mexico in 2012, management with MA was extended to ten-week gestations and the SSDF has further enhanced efficiency; improved outcomes and fewer follow-up visits has lowered costs. However, numerous barriers still impede access to safe abortion. In Costa Rica, misoprostol is restricted to treatment of gastric ulcers, the labelled indication, and quarterly reporting is required of pharmacists who dispense it to prevent its use for abortion.
(Ely, Hales et al. 2018) [United States, Republic of Ireland, United Kingdom]	To examine the experiences of abortion fund patients in the United States (USA) and Republic of Ireland (RI), Northern Ireland (NI) and Isle of Man (IM) to compare abortion fund patient experiences across these developed nations for the first time	Select abortion fund patients within each country (n=6,340 cases; 3,995 from the USA and 2,345 from the RI, NI and IM)	Cross-sectional descriptive analysis	In a linear regression model where procedural costs were regressed on a binary variable representing the respective datasets and the number of weeks pregnant, weeks pregnant explained 44% of the variation in procedural costs, while the datasets (representing organisation or country) explained 5% of the variation, both of which were significant.
(Esia-Donkon, Darteh	To explore the pre- and post-	Young people (aged 12 to	Qualitative: in-depth	Comparing the cost at the clinic to facilities elsewhere, the former was

Author, year [country]	Aim/objective(s)	Population	Study type	Summary of main findings
et al. 2015) [Ghana]	experiences of young people (aged 12 to 24) who had their abortion three months prior to the study	24) who had their abortion three months prior to the study at the Planned Parenthood Association of Ghana Cape Coast clinic	interviews	economical. The facility coordinator indicated that the facility usually provides free services to young people who genuinely did not have money to pay for the services received.
(Font-Ribera, Perez et al. 2009) [Spain]	To describe the determinants of the voluntary pregnancy interruption (IVE) delay until the second trimester of pregnancy in the city of Barcelona, between 2004 and 2005	Women who reside in the city of Barcelona who obtained abortions for physical or mental health issues between 2004 and 2005 (n=9,175)	Cross-sectional study	The time of gestation and the moment in which the IVE occurs is important because it determines the abortion method that can be used, the risk of complications and mortality, and economic cost of the intervention. As gestational age increases, the abortive technique is more invasive and expensive, and poses a greater risk to the health of women; it is also more difficult to find a provider willing to perform it. Public centres have higher proportions of second trimester IVEs than private centres.
(Foy, Penney et al. 2004) [United Kingdom]	To evaluate the effectiveness and efficiency of a tailored multifaceted strategy, delivered by a national clinical effectiveness programme, to implement a guideline on induced abortion	All 26 hospital gynaecology units in Scotland providing induced abortion care	Randomized controlled trial	The mean cost of the intervention per gynaecology unit was £ 2067, with the audit and feedback component accounting for half of this cost. Intervention costs also included staff time, travel, consumables, and administration for the unit educational meetings.
(Foster and Kimport 2013) [United States]	To analyse data on women who sought and received an abortion at or after 20 weeks' gestation for reasons other than fetal anomaly or life endangerment	People who were seeking abortions after 20 weeks gestation across 16 sites (n=272) and people who presented for first trimester abortions (n=169)	Mixed methods: qualitative data from interviews and quantitative data for logistic regression	Insurance coverage complexities were a significant barrier for some abortion seekers. The increased cost of abortions at later gestational is likely to cause significant funding and insurance issues. Delays securing public or private insurance were greater among people seeking second trimester abortions (41%) than people seeking first trimester abortions (20%).
(Gallo and Nghia 2007) [Vietnam]	To understand the determinants of delaying obtaining abortion until the second trimester	Clients presenting for an abortion at 13–24 weeks of gestation in 5 health facilities in 3 provinces in Vietnam (n=60); abortion service providers (n=6)	Qualitative: semi-structured interviews	The high fees charged at the facilities for second-trimester abortion could further delay late-term abortions as a result of women needing additional time to accumulate funds. Providers differed in their views of how much should be charged for abortion. One provider suggested that fees should be reduced for women who cannot afford them. Yet, providers in this study and past studies have argued that abortion fees should be set high enough to discourage reliance on abortion.
(Gan, Zhang et al. 2011) [China]	To evaluate Chinese healthcare providers' knowledge of MA, to understand their perspectives regarding the main challenges to increasing its uptake, to understand their preferences for	Abortion service providers from Shenzhen and Henan (n=658)	Cross-sectional study	In the rural area, there was not much difference in cost between MA and surgical abortion, and both methods were very cheap. In the urban area, even if there was a difference in cost between the two methods, the income associated with abortion was low compared with that for other services (e.g. obstetric and gynaecologic surgeries); therefore it was unnecessary for the providers to consider remuneration when

Author, year [country]	Aim/objective(s)	Population	Study type	Summary of main findings
	specific abortion methods, and to investigate the role of remuneration on the decision-making process			recommending an abortion method. In 5.6% of cases, the abortion clinic charged for abortion counselling, and in 10.9% of cases it charged for abortion follow-up.
(Gibb, Donaldson et al. 1998) [United Kingdom]	To measure women's preferences and strength of preferences (expressed in terms of willingness-to-pay) for MA versus surgical vacuum aspiration	Women receiving abortion services at Aberdeen Royal Hospitals NHS Trust (n=50)	"Willingness to pay' (WTP) technique	When the costs to the NHS of providing the medical and surgical methods were compared, MA was found to use 8% less NHS resources than surgical. If a choice had to be made about which service to provide, WTP and cost data indicate that MA should be chosen. As use of MA increases, the costs of maintaining the surgical facility may rise.
(Glenton, Sorhaindo et al. 2017) [Bangladesh, Ethiopia, Nepal, South Africa and Uruguay]	To explore factors influencing the implementation of role expansion strategies for non-physician providers to include the delivery of abortion care	Non-physician providers of abortion services within multiple health facilities in each country non-physician providers	Qualitative: Case study, literature review, and key informants	While managers' and co-workers' attitudes towards the use of non-physician providers varied, female clients focused less on the type of health worker and more on factors such as trust, privacy, cost, and closeness to home. Health systems factors also played a role, including workloads and incentives, training, supervision and support, supplies, referral systems, and monitoring and evaluation.
(Graff and Amoyaw 2009) [Ghana]	To identify barriers to sustainable MVA supply	Literature review; stakeholders involved with MVA policy, manufacturing, procurement, training, and provision (n=70)	Situational assessment	MVA services are reportedly free when it is an emergency and the woman cannot afford the cost; otherwise prices ranged from US$ 11—33 in public regional/district-level facilities, US$ 54—163 USD in private hospitals and clinics, and US$ 3—27 in private maternity homes. In addition to charging less than physicians for MVA, nurse midwives reported lower profit margins and less sustainable access to MVA supply. Low-volume, low-income nurse midwives were most likely to report that the cost of MVA equipment is too expensive, especially in rural areas. Some nurse midwives reported that even when funds are available at health facilities, there is often no source for supply in rural areas.
(Gresh and Maharaj 2011) [South Africa]	To examine the acceptability of medical abortion among young people in Durban, South Africa<br><br>To investigate the potential demand for and applicability of the method among women in South Africa	Sexually active women at the University of Durban who are under 30 years of age (n=20)	Qualitative in-depth interviews	Abortion services are free in public hospitals under the current government legislation. Abortion services at private facilities cost approximately ZAR 900.
(Grossman, Ellertson et al. 2004) [global]	To review protocols and existing evidence on follow-up care for abortion	Global literature on follow-up visits for abortion care	Literature review	The costs to the abortion clinic of the routine follow-up visit are likely multiple. The high proportion of "no-shows" complicates scheduling, burdens administrative systems, and likely has the net effect of wasting clinician staff time and increasing waiting times for patients. If a clinic

Author, year [country]	Aim/objective(s)	Population	Study type	Summary of main findings
				counts on 50% of patients skipping the follow-up visit but more attend on a given day, the clinic may become overwhelmed and patients will have long waits. If fewer patients attend follow-up visits than anticipated, the clinic may find itself over-staffed. Inefficiencies related to missed follow-up appointments may reduce providers' availability to perform abortions or other services and drive up the costs of care.
(Grossman, Baum et al. 2014) [United States]	To rapidly assess the change in abortion services after the first three provisions [of a restrictive law] went into effect	All licensed abortion facilities in the state of Texas	Observational study	After the law changed, the cost of MA increased. Under HB2, providers could either use the regimen included in the Mifeprex® labelling with 600 mg of mifepristone, which is considerably more expensive than the evidence-based regimen, or they could use the drug dosages in the 2005 ACOG Practice Bulletin on MA. This regimen was interpreted as allowing the use of mifepristone 200 mg followed two days later by misoprostol 800 mcg orally, a regimen supported by limited evidence. The cost of the procedure increased at most facilities offering the regimen with 600 mg of mifepristone.
(Guttmacher, Kapadia et al. 1998) [South Africa]	To examine the policies that have regulated accessibility of abortion and assesses their impact on reproductive health	South African policies	Review of policies and related evidence	Data from the Medical Research Council indicate that procedures for managing incomplete induced abortions are not cost-effective, and they are not safe or widely accessible outside the major hospital centres.
(Harries, Lince et al. 2012) [South Africa]	To better understand what doctors, nurses and hospital managers involved in second trimester abortion care thought about these services and how they could be improved	Abortion-related service providers and managers in the Western Cape Province, South Africa (n=19)	Qualitative: In-depth interviews	At medical induction sites, most participants thought the combined mifepristone–misoprostol regimen would improve service capacity, though they were concerned about cost. In the medical induction service, doctors were generally aware of the superior efficacy of the combined mifepristone–misoprostol regimen compared with misoprostol used alone, but they were concerned about the cost of mifepristone. Several pointed out that using mifepristone might be cost-effective, if it reduced the duration of hospitalization. Introducing the mifepristone regimen at public sector facilities that lack trained and willing D&E providers might improve the capacity of these services to meet the needs of women seeking second trimester abortion, especially if a low-cost mifepristone product becomes available.
(Harvey and Gaudoin 2005) [United Kingdom]	To compare nurse-led service for termination of pregnancy with previous service on waiting times and cost	Data on nurse-led clinics at Southern General Hospital (n=48)	Review/analysis of descriptive statistics	The calculated annual cost of the medically led clinic was £ 37,495 - 0.40 whole-time equivalents (WTE) for a staff grade doctor (£ 22,598) and 0.43 WTE for an F-grade staff nurse (£ 14,897). The clinic could be run in its present form by a G-grade nurse at an annual cost of £ 25,943 (0.80 WTE) - a financial savings of almost 40%.
(Henshaw, Naji et al. 1994) [United	To estimate and compare the relative costs to the NHS of	Women receiving legal abortion services at a	Cost analysis	MA used 8% less of NHS resources (£ 343 vs. £ 374) when compared to vacuum aspiration. The conclusions are likely to be valid for most NHS

Author, year [country]	Aim/objective(s)	Population	Study type	Summary of main findings
Kingdom]	providing legal termination of pregnancy using MA or vacuum aspiration; also to estimate and compare the relative financial costs incurred by women undergoing these procedures	Scottish teaching hospital (n=363)		units. Substantial NHS resource savings are unlikely to follow the widespread introduction of MA services in their current form.
(Henshaw 1995) [United States]	To provide information on the percentage of women who travel long distances to obtain abortion services, the availability of abortion providers for second trimester services, the need to make more than one trip to the abortion facility and the amount abortion providers charge for services. In addition, it presents a measure of antiabortion harassment	Abortion providers in the United States (n= 1,525)	Cohort	In 1993, on average, nonhospital providers charged $604 for an abortion at 16 weeks and $1,067 at 20 weeks; the median charges were slightly lower. Fees ranged as high as $2,500 at 16 weeks and $3,015 at 20 weeks. In the period between the two previous surveys, 1986 to 1989, abortion charges rose more than the cost of living. For women who seek abortions with general anaesthesia, costs may be significantly higher. Relatively few nonhospital providers—33% of abortion clinics, 17% of nonspecialized clinics and 17% of physicians' offices—reported offering general anaesthesia. Sixty-three per cent of facilities that offered general anaesthesia charged extra for it, which varied according to provider type, averaging $114 at abortion clinics, $136 at non-specialized clinics and $306 in physicians' offices.
(Henshaw and Finer 2003) [United States]	To document the current status of abortion service accessibility in the United States, on the basis of data collected in a survey of all known U.S. abortion providers conducted in 2001–2002 by the Alan Guttmacher Institute (AGI)	Facilities documented in the AGI Abortion Provider Survey (n=1,819)	Quantitative; survey	Providers reported that about 13% of abortions are reimbursed by Medicaid. An estimated 13% of abortions are covered by private insurance billed directly by the facility. The proportion of providers that bill private insurance for their clients' abortions is higher than average for nonhospital providers performing fewer than 30 abortions per year and for physicians' offices (45% vs. 27%). The mean charges for a mifepristone abortion and for a methotrexate abortion were $490 and $438, respectively. Providers who used 600 mg of mifepristone charged $74 more, on average, than providers who used 200 mg. More than two in five providers (43%) charged between $400 and $499 for mifepristone, and 38% charged $500 or more. For 80% of MA providers, the basic charge for a MA included the cost of a subsequent vacuum aspiration, should an incomplete abortion or continuing pregnancy occur.
(Henshaw, Adewole et al. 2008) [Nigeria]	To examine and document the characteristics of women who are admitted to hospitals for complications from unsafe abortion (or to obtain an induced abortion), the conditions under	All women who were admitted for treatment of pregnancy loss during the study period as well as those who came to request an abortion (n=2,093) in 33	Survey using structured questionnaires in face-to-face interviews	The cost of using MVA to perform an abortion was slightly higher than the cost of using D&C (3,446 vs. 3,090 naira). Because MVA is a relatively new technology, Nigerian providers may not yet have factored this lower cost into the amount they charge clients or they may be charging for single-use MVA kits. The instruments used for D&C procedures, on the other hand, last for a long time, resulting in lower

Author, year [country]	Aim/objective(s)	Population	Study type	Summary of main findings
	which women obtain abortions, the nature and severity of complications resulting from unsafe abortions, the type of treatment used for abortion complications, and the cost of treatment	hospitals in eight states across Nigeria. Women reporting spontaneous abortions were included because it is not always easy to distinguish such events from induced abortions.<br><br>The health care provider (e.g. attending physician or nurse) for each surveyed woman was interviewed to provide matched physician data for each respondent		costs for individual women. Unsafe abortion absorbs health care resources and creates a major problem in both the more developed and more traditional areas of Nigeria.
(Hollander 1995) [United Kingdom]	To describe evidence on the effectiveness of MA regimens	Data from two previous studies on women receiving MA care	Synthesis of two other studies	The combination of mifepristone followed by vaginal misoprostol is considerably less costly than the standard MA regimen of mifepristone and gemeprost. Nurses can manage most of this procedure that costs one-fourth of the standard regimen of mifepristone and gemeprost.
(Htay, Sauvarin et al. 2003) [Myanmar]	To describe the process undertaken by the Department of Health to address the issue of abortion complications, by integrating PAC and contraceptive service delivery into existing health care services	Women treated for postabortion complications in the hospitals (one station and four township hospitals) (n=170)	Mixed methods: structured interviews, focus group discussions, inventory of equipment and supplies within health facilities, observation checklist to assess clinical care, an informal ward round, surveys	Hospital staff members keep a fund with donations for treatment of poor patients. The community supports transportation and food. The midwife accompanies the patient to hospital to get treatment free of charge if she is poor. The midwife makes this decision, as she knows who in the village is rich or poor.
(Hu, Grossman et al. 2009) [Mexico]	To assess the comparative health and economic outcomes associated with three alternative first-trimester abortion techniques in Mexico City and to examine the policy implications of increasing access to safe abortion modalities within a restrictive setting	Computer-based model simulation looking at three alternative first-trimester abortion techniques	Cost analysis and projections	Given the baseline assumptions, clinic-based MVA was least costly and most effective. Hospital-based MVA was as effective as clinic-based MVA but more costly. D&C and MA using vaginal misoprostol provided comparable benefits, but costs associated with the latter were substantially lower. General results were the same when time and personal costs were included, though average per-woman costs increased by 6 to 34%. In comparison to the magnitude of health gains associated with all modalities for safe abortion, the relative differences between strategies were more pronounced in terms of their

Author, year [country]	Aim/objective(s)	Population	Study type	Summary of main findings
				comparative economic costs. Even assuming 100% access to D&C, shifting to clinic-based MVA would save nearly $100,000 per 1,000 women. Relative to unsafe abortion, these savings double in magnitude.
(Hu, Grossman et al. 2010) [Nigeria and Ghana]	To explore the policy implications of increasing access to safe abortion in Nigeria and Ghana, the authors developed a computer-based decision analytic model that simulates induced abortion and its potential complications in a cohort of women, and comparatively assessed the cost-effectiveness of unsafe abortion and three first-trimester abortion modalities: hospital-based D&C, hospital- and clinic-based MVA, and MA using misoprostol	Available clinical and cost related data for abortion care services in Nigeria and Ghana	Decision analytic model	Costs related to complications ranged from 9—26% of total costs for surgical strategies and were substantially higher for MA with misoprostol (31—57% of total costs), largely due to method failure without complications. In Nigeria, clinic-based MVA was the least costly and most effective option; therefore, it dominated the competing strategies. While equally as effective as clinic-based MVA, hospital-based MVA was the most expensive strategy. D&C and MA using vaginal misoprostol provided comparable benefits although the latter was less costly. In Ghana, MA was the most cost-effective option owing to its low procedural cost (approximate one-third the cost of clinic-based MVA). Clinic-based MVA was more effective than MA but was associated with a cost of $16,855 per life-year gained. Hospital-based MVA and D&C were more expensive and no more effective than clinic-based MVA; therefore, they strongly dominated. When the analysis was conducted from a limited health payer perspective that considers the direct medical costs of only abortion-related complications (not the original procedure), MVA (either clinic- or hospital-based) was the least costly and most effective strategy in both countries.
(Ilboudo, Greco et al. 2016) [Burkina Faso]	To estimate the costs of six abortion complications treated in two public referral hospital facilities in Ouagadougou and the cost saving of providing safe abortion care services	PAC-registers<br><br>Key-informant interviews in maternity wards and in hospital facilities	Retrospective review	Across six types of abortion complications, the mean cost per patient was US$ 45.86. Treatment costs of abortion complications in both hospitals ranged from US$ 23.71 for an incomplete abortion to US$ 85.08 for a case of infection/sepsis. Incomplete abortion and haemorrhage were the least expensive services, at US$ 23.71 and US$ 26.30 per case, respectively. Uterus perforation and infection/sepsis were the most expensive services at the tertiary level teaching hospital at a cost of US$ 73.76 and US$ 94.39, respectively. The average per-patient cost of treating any complication of abortion was US$ 45.86. This cost was higher in the tertiary teaching hospital compared to the secondary-level hospital: US$ 51.09 versus US$ 36.50, respectively. The total cost to these two facilities for treating the complications of abortion was US$ 22,472.53 in 2010, equivalent to US$ 24,466.21 in 2015. Provision of safe abortion care services to women who suffered from complications of unsafe abortion and who

Author, year [country]	Aim/objective(s)	Population	Study type	Summary of main findings
				received care in these public hospitals would only have cost US$ 2,694, resulting in potential savings of more than US$ 19,778.53 in that year.
(Jerman and Jones 2014) [United States]	To document several measures of access to abortion services in the United States--cost, gestational age limits, and harassment--using data from the Guttmacher Institute's most recent Abortion Provider Census<br><br>To assess regional differences in abortion access	All known abortion-providing facilities in the United States were asked to respond to the survey (n=1,720)	Survey	In 2011 and 2012, the median charge for a surgical abortion at ten weeks gestation was $495. By comparison, the inflation-adjusted charge for the same procedure in 2009 was $503, suggesting little to no change. The cost of abortion varied by facility type and gestational age. Abortion clinics charged the least for a surgical abortion at ten weeks' gestation ($450); abortions were most expensive at physicians' offices ($550). Facilities with the largest caseloads charged the least ($450), and those that performed fewer than 30 procedures per year charged the most ($650). In 2011, the median charge for early medication abortion was similar to surgical abortions at ten weeks' gestation at $500, although the average amount women paid was slightly higher than that for a surgical abortion at ten weeks ($504). Adjusting for inflation, the median charge for early medication abortion in 2009 was $524.Abortions at 20 weeks' gestation typically take two or more days to complete and involve greater skill and resources. The median charge for an abortion at 20 weeks' gestation in 2011 and 2012 was $1,350. That the number of early medication abortions increased during this period and could be a both a cause and a consequence of the small drop in the cost of this procedure; greater demand may have resulted in a lower cost, or lowering the cost could have made it more affordable for more women. Median charges for abortion showed little to no change over time and may represent an effort on the part of providers to keep services affordable, in spite of increases in both restrictions and related costs of health care provision.
(Johnston, Gallo et al. 2007) [Uganda]	To establish the utility of the model and assess the order of magnitude of the difference in costs of care when different service delivery approaches are used	Data from the Ugandan Safe Motherhood Programme Costing Study	Cost estimation and analysis	The 'savings' model suggests that regardless of whether abortion law is restrictive or liberal, a recommended approach based on recommended technical interventions for abortion-related care delivered at a decentralised level could substantially reduce costs compared to a conventional approach. For example, changing from the restricted conventional setting to the restricted-recommended setting decreases the mean cost per unsafe abortion complication case by 43% (i.e. from $45 to $25). Similarly, the use of the liberal-recommended scenario instead of the liberal conventional scenario reduces costs by 81% (i.e. from $34 to $6). The greatest reduction in costs is associated with changing from the conventional system within a restrictive law setting (restrictive-conventional) to a planned, decentralised system

Author, year [country]	Aim/objective(s)	Population	Study type	Summary of main findings
				within a setting that allows legal, elective abortion (liberal-recommended). This change decreases the mean case cost from $45 to $6, representing a savings of 86%. Although the model calculates the estimated savings as dollars, the saved resources (e.g. staff time, equipment, and supplies) are unlikely to be realised as a reduction in health care costs. Instead, these savings likely would be shifted to other patient services.
(Johnston, Oliveras et al. 2010) [Bangladesh]	To present collected health system expenditure data and then used the Savings model to estimate comparative costs to the health system of providing menstrual regulation and care for abortion complications	Data on menstrual regulation and abortion complication care from select facilities in Dhaka, Barisal, and Sylhet divisions	Cost comparison and analysis	This study analysis confirmed that on a per-case basis, the incremental costs of providing menstrual regulation and related care following recommended practices (including use of MVA for UE, providing services in outpatient facilities, having mid-level providers offer UE and other care, and providing contraceptive counselling and services) are much lower than those associated with providing care for abortion complications. The application of Bangladesh data to the Savings model demonstrates that on a per-case basis, provision of menstrual regulation care cost 8-13% of the cost of treatment for severe abortion complications, depending on the level of care at which menstrual regulation and PAC were offered.
(Johnston, Akhter et al. 2012) [Bangladesh]	To assess incremental health system costs of service delivery for abortion-related complications in the Bangladesh public health system and confirmed that providing PAC with vacuum aspiration is less expensive than using D&C	Public-sector health facilities in Bangladesh (n=17)	Qualitative: purposive sampling and informational interviews	Providing PAC with vacuum aspiration is less expensive than using D&C. Treatment for severe complications, including surgical care for vaginal, cervical, and uterine lacerations, was three times as expensive as treatment for moderate complications. Care for moderate complications was less expensive to the health system when provided at the primary as opposed to the tertiary level. Implementing several evidence-based best practices, such as replacing D&C with vacuum aspiration, reducing use of high-level sedation, authorizing mid-level providers to offer PAC, and providing postabortion contraceptive counselling and services to women while still at the health facility, could increase the quality and cost efficiency of PAC.
(Jones and Weitz 2009) [United States]	To provide a legal review of a set of laws that directly target abortion providers, which ultimately has a significant capacity to reduce access to and quality of abortion care in the United States by making the provision of abortion more difficult and costly and providing	Review of laws in the United States	Review	Twelve states restrict abortion coverage in insurance plans for public employees, and five states restrict insurance coverage of abortion in private insurance plans. Combined with the public controversy over abortion, confusion over insurance coverage prompts many women to pay out of pocket rather than seek coverage clarification. Some abortion clinics do not accept third-party payers.

Author, year [country]	Aim/objective(s)	Population	Study type	Summary of main findings
	strong incentives for physicians not to offer abortion services			
(Jones, Ingerick et al. 2018) [United States]	To examine differences in abortion service delivery according to the policy climate in which clinics must operate	All known abortion-providing facilities in the United States (n=1,662)	Cross-sectional study	Abortions at later gestational lengths require greater technical skills and resources; in turn, it costs more. In 2014, the median charge for an abortion at 20 weeks gestation was $1,195. Meeting state abortion requirements, such as providing in-person counselling and converting to Ambulatory Surgical Centres, can be expensive. Presumably some of these costs are passed on to patients in hostile states. A greater reliance on advanced practice clinicians in supportive states may reduce the cost of care compared with facilities that relied almost exclusively on physicians.
(Kacanek, Dennis et al. 2010) [United States]	To investigate the following questions: What are providers' experiences with Medicaid reimbursement for abortion in cases of rape, incest and life endangerment? What is the process for applying Medicaid reimbursement? What factors facilitate or hinder reimbursement?	Abortion providers in Florida, Idaho, Kansas, Kentucky, Mississippi, Pennsylvania, South Dakota, and Wyoming, where Medicaid funding is limited (n=25)	Qualitative: purposive interviews	The median costs were $450 for a medication abortion, $425 for a first trimester surgical abortion, and $900 for a second-trimester abortion. Of the 245 reported abortions that should have qualified for Medicaid reimbursement, 143 were not reimbursed. Of the 102 that were reimbursed, 99 were in one state; within that state, 27 qualifying abortions were not reimbursed. Eighteen respondents reported that no qualifying abortions were reimbursed.
(Kamali, Hohmann et al. 1998) [Germany]	To determine the best approach to inducing abortion in order to minimize the psychological and physical stress to the patient	Abortion patients between the 15th and 24th week of gestation (n=79) between 1992-1994	Randomized, prospective study	For the cost analysis per patient, costs incurred were about the same for Group 1 [Dinoproston (Prepidi I)] (190 DM), Group 2 [Sulproston Gel (Nalador)] (183 DM), and Group 4 [Gemeprost vaginal suppository (Cergem, I mg, max. 4 rng/24h)] (181 DM). Group 3 incurred significantly higher costs per patient (317 DM). These cost analyses do not include personnel costs. The cost of gemeprost treatment was lower in comparison to repeated use of dinoprostone. Applying cervical gels had a higher expenditure of time and personnel than the application of Cergem suppositories. The Sulprostone infusion times in Group 4 continued at shorter supervision times and also led to a reduction in costs.
(Koontz, Molina de Perez et al. 2003) [El Salvador]	To examine the introduction of MVA services in a regional training hospital in El Salvador and report on its implications	Women who present with incomplete abortion at a training hospital in El Salvador (n=154)	Controlled clinical trial	Overall time in hospital was significantly reduced for MVA patients (19.7 hours) as compared to the SC patients (27.2 hours). The total cost of an MVA procedure and hospital stay was significantly lower than the SC procedure, roughly a 13% reduction in total cost ($54 vs. $62). The most substantial costs were related to the overhead costs of hospital stay, followed by personnel costs and costs of services such as lab tests

Author, year [country]	Aim/objective(s)	Population	Study type	Summary of main findings
				and meals, of which the costs were significantly less for MVA patients than SC patients. Though a small proportion of total costs, the cost of supplies and equipment for MVA was significantly higher than for SC.
(Lalley, Jelsema et al. 2001) [United States]	To complete a cost effectiveness evaluation for women who received either prostaglandin E2 (PGE2) or misoprostol for a second trimester termination of pregnancy with either a living or dead fetus	Medical records of women seeking abortion care services at Butterworth Hospital in Grand Rapids, Michigan (n=78)	Cost comparison and analysis	Average treatment cost per patient for misoprostol was $298.86 and for PGE2 was $548.21, a savings of $249.35 for misoprostol (P<0.01). The mean cost of treatment when initial medication was successful was $2.40 for misoprostol and $483.90 for PGE2 (P<0.01). Although misoprostol was associated with a higher failure rate, it was more cost-effective than PGE2 for second trimester termination, even when misoprostol alone was unsuccessful in terminating pregnancy.
(Levin, Grossman et al. 2009) [Mexico]	To assesses abortion outcomes and costs to the health care system in Mexico City in 2005 at a mix of public and private facilities prior to the legalization of abortion	Three public hospitals and one private clinic in Mexico City	Cost estimates and projections	The average cost per abortion with D&C was US$ 143. MVA was US$ 111 in three public hospitals and US$ 53 at a private clinic. The average cost of MA with misoprostol alone was US$ 79. The average cost of treating severe abortion complications at the public hospitals ranged from US$ 601 to over US$ 2,100. These estimates represent the opportunity cost of all resources used in the treatment of incomplete abortion and other complications from the health system perspective, including personnel, drugs, disposable supplies, and medical equipment for inducing abortion or treating incomplete abortions and other complications.
(Li, Song et al. 2017) [China]	To investigate the efficacy, safety, and acceptability of low-dose mifepristone combined with self-administered misoprostol for ultra-early MA	Women with ultra-early pregnancy and regular menstrual cycles who sought MA from an institute of obstetrics and gynaecology at four hospitals (n=744)	Randomized control trial	The mean time and cost expenditures per participant were greater in the hospital administration group (557 minutes, US$ 40.12) than in the self-administration group (18 minutes, US$ 1.96). Generally, one hospital visit involving serum b-hCG detection (US$ 7.67/test) and vaginal ultrasound examination (US$ 11.49/scan) costs at least US$ 19.15, and at least 3 hours were required to get the results.
(Limacher, Daniel et al. 2006) [Canada]	To compare the costs (considered from the perspectives of society, the health care system, and the patient) of four options for early medical and surgical abortion in Ontario	Available data on abortion procedures in Ontario	Cost analysis	In the model, medical options for early abortion compare favourably with surgical options in terms of total cost to society, the health care system, and the patient. For society and the health care system, the direct costs of MA are less than those of surgical abortion, but for patients the direct costs of MA are higher. Although the procedures and outcomes for early surgical abortion are essentially identical in the hospital and clinic settings, the clinic has a cost advantage from the perspectives of both society and the health care system because of its lower overhead and its greater efficiency that is due to specialization in a single procedure.
(Lince-Deroche, Constant et al. 2015)	To assess women's costs of accessing second-trimester labour	Cost data for women accessing second-trimester	Study 1: Randomized controlled trial	Some women reported visiting private doctors before accessing care in the public sector. More women reported paying a private doctor's fee

Author, year [country]	Aim/objective(s)	Population	Study type	Summary of main findings
[South Africa]	induction and D&E services at four public hospitals in Western Cape Province, South Africa	labour induction and D&E services at four public hospitals in Western Cape Province	Study 2: Repeated, cross-sectional observations over time	than visiting a private doctor's office. This discrepancy may have been the result of visiting "backstreet providers" who charged a fee but did not work in a formal office.
(Lince-Deroche, Fetters et al. 2017b) [South Africa]	To estimate the costs and cost effectiveness of providing first-trimester medication abortion and MVA services to inform planning for first-trimester service provision in South Africa and similar settings	Data from three facilities on cost of medication abortion and MVA services	Cost analysis using secondary data	Considering the base case cost estimates, medication abortion was less costly than MVA. However, considering the uncertainty analysis ranges, the plausible ranges for the costs of the two procedures overlap, suggesting that the costs of the two procedures could be the same in some circumstances. Personnel costs were the largest contributor to total costs for both procedures. Given the extremely low rate of hospitalization among women who had a MA and a study follow-up visit (0.4%, 3/714) in the operations research study, the contribution of hospitalization costs to the total average cost of medication abortion is minimal. Because no women who had an MVA were hospitalized, there are no hospitalization costs for MVA. Laboratory costs are zero for both procedures because no site conducted investigations requiring outsourced testing.
(Lince-Deroche, Constant et al. 2018) [South Africa]	To estimate the costs and cost effectiveness of providing three safe second-trimester abortion services (D&E), medical induction with mifepristone and misoprostol (MI-combined), or medical induction with misoprostol alone (MI-misoprostol) in Western Cape Province, South Africa	Cases presenting for second trimester care at public hospitals in Western Cape Province	Repeated cross-sectional observations	D&E was least costly at $88.89 per woman seen. Medical induction with a combined regimen was less costly than induction with misoprostol alone at $298.03 and $364.08, respectively. However, overlap in plausible ranges in costs for the two medical procedures suggests that the two procedures may cost the same in some circumstances.
(Magotti, Munjinja et al. 1995) [Tanzania]	To compare the cost effectiveness of managing abortions by newly introduced MVA technique with contemporary evacuation by curettage	Patients with incomplete abortions admitted to Muhimbili Medical Center (n=199)	Questionnaire and cost analysis	The direct costs revealed a cost differential of electric vacuum aspiration over evacuation by curettage of Tshs 776.9 (US$ 2.6). MVA is more cost-effective than contemporary evacuation by curettage.
(Manouana, Kadhel et al. 2013) [Guadeloupe]	To describe the typical profile and to assess the motivations of women who underwent illegal abortion with misoprostol in Guadeloupe (French West Indies)	Women who consulted the obstetric-gynaecological department at CHU de Pointe-à-Pitre/Abymes (Guadeloupe) after failure or	Descriptive, prospective	At Pointe-à-Pitre University Hospital, part of the payment (€ 50-60) remains the responsibility of the patient and can be supported by a health insurance supplement or exempted. In comparison, illegal abortion with misoprostol can cost less than € 1. The misoprostol box costs around € 19 for 60 pieces, a little less than 32 cents per pill. The

Author, year [country]	Aim/objective(s)	Population	Study type	Summary of main findings
		complication of an illegal abortion with misoprostol		purchase price misoprostol could reportedly reach € 10 in illegal circuits. These abortions give rise to a lucrative and fraudulent parallel economy that can motivate some to promote and disseminate this demedicalized method.
(Medoff 2008b) [United States]	To examine whether state restrictive abortion laws have a significant impact on the price abortion providers charge for providing abortion services	National data set of state-level data for the years 1982, 1992, and 2000	Econometric analysis of price data and price elasticity	The enforcement of a parental involvement law has a statistically and numerically positive impact on a provider's price of an abortion. The enforcement of a parental involvement law increases the price of an abortion by 9.7—13%. The enforcement of a mandatory counselling law has a statistically and numerically significant cost impact on a provider's price for an abortion. The enforcement of a mandatory counselling law causes abortion providers to raise their prices by 9.3% (or US$ 35).
(Messinger, Mahmud et al. 2017) [Bangladesh]	To investigate the knowledge, attitudes and practices regarding mHealth of both menstrual regulation (MR) clients and formal and informal sexual and reproductive healthcare providers in urban and rural low-income settlements in Bangladesh	MR clients (n=24) purposively selected based on the number of children they had and educational status<br><br>Formal (n=10) and informal (n=16) close-to-community health providers<br><br>Doctors, paramedics, and programme staff of the non-governmental organisation clinics (n=8)	Qualitative approach using in-depth interviews	Both formal and informal providers have to bear the cost of their mobile phone credits in order to communicate with their clients. All expressed that providing a mobile phone service makes their clients feel content and enables both current and new clients to contact them regarding community health services. However, all formal providers, including governmental providers, mentioned that mobile phone costs are not usually included in their salary. All providers said that they do not mind paying the phone bills as long as it is for a good cause that is serving the community.
(Murthy and Creinin 2003) [global]	To conduct a complete review that includes background information on the history and incidence of abortion, who chooses to get an abortion, who provides that service and at what cost	Women who chose to have a MA	Literature review	When patients are covered by private insurance, the physician is reimbursed by the insurers at an agreed rate. Unlike other service provided in the United States, a single combination fee is not charged or paid with MA. Because of the method of Food and Drug Administration (FDA) approval, the mifepristone company can only sell directly to the physician who can then dispense that medication directly to the patient. Each tablet costs US$ 90; at the FDA recommended dose, each abortion would cost US$ 270. To provide this service, the physician is obligated to accept this initial capital outlay. In contrast, misoprostol costs about US$ 1.03 for a 200-µg tablet. A full dose of misoprostol for one MA would be an additional US$ 4 per patient (using 800 µg as per the alternative regimen).

Author, year [country]	Aim/objective(s)	Population	Study type	Summary of main findings
(Ordinioha and Brisibe 2008) [Nigeria]	To find out the motivations and experiences of medical practitioners in private clinics who still provide abortion services despite abortion being illegal in Nigeria	34 doctors at 15 clinics	Small-scale survey	Respondents charge an average of 5,000 naira for an eight-week pregnancy. All clinics considered patients seeking abortion to be the private patients of the doctor that provided the service, so the service charge was shared to reflect the fact that the provider had to bear the consequences for any complication that arose. Most clinics (75%) collected money just for the consumables and the use of the clinic's theatre, while 25% had a fixed amount paid to clinic that varied with the gestational age of the pregnancy terminated.
(Parmar, Leone et al. 2017) [Zambia]	To provide estimated costs of abortion care and PAC services in Zambia	Provision of safe abortion and PAC services at the University Teaching Hospital (UTH) in Lusaka	Mixed methods	The costs for MA and for treating incomplete abortion are similar (US$ 33); MVA is slightly more expensive (US$ 39). For UTH, the cost of PAC for unsafe abortion is estimated at US$ 109,811 per year, 13 times greater than the cost of safe abortion. Providing MA and MVA cost significantly less than treating sepsis and shock. PAC for unsafe abortion was provided nine times more than safe abortion. Although MA costs less than MVA, when estimated over a year, MA at UTH costs more (US$ 5,898 vs. US$ 1,772) simply because more MA services are provided than MVA. Safe abortion on average costs US$ 14 per case less than PAC following unsafe abortion.
(Payne, Debbink et al. 2013) [Ghana]	To describe major barriers to widespread safe abortion in Ghana through interviews with Ghanaian physicians on the front lines of abortion provision	Ghanaian physicians known for their commitment to safe reproductive health services (n=4)	Qualitative: Open-ended interviews with key informants	Though services are more affordable at earlier stages of pregnancy, the costs often exceed what most women are able to pay. For women seeking second trimester abortion, physicians asserted that most safe second trimester surgical abortion is available only through a small handful of private providers in two urban centres, and the cost for this procedure is prohibitive for many women.
(Penney, McKessock et al. 1995) [United Kingdom]	To estimate the cost and effectiveness of a low-dose regimen of mifepristone and misoprostol	Women with pregnancies of up to 63 days requesting MA at one NHS hospital in Scotland (n=360)	Controlled clinical trial	The NHS drug costs of the reduced regimen are less than one quarter that of the standard regimen (and also costs less than a suction termination). Patients are managed almost entirely by pregnancy counselling nurses.
(Pheterson and Azize 2005) [Anguilla, Antigua, St Kitts, Sint Maarten]	To contribute to an improvement in abortion care in the region, the study sought to identify practitioners who were qualified, informed, and potentially influential	Interviews with physicians (n=26) of whom 16 provided abortion care	Qualitative in-depth interviews	Pharmacies sell Cytotec by the pill at an extreme mark up, and incomplete abortions are reported as common. When patients could not afford the estimated US$ 560 for an abortion, physicians referred them to buy medical abortions at pharmacies and return to the physician in case of complications.
(Pillai, Welsh et al. 2015) [United Kingdom]	To assess the applicability, acceptability and cost implications of introducing the MVA technique with local anaesthesia for fully	Women seeking abortion services within an outpatient setting at a Pregnancy Advisory Service within a	Review of routinely collected facility data on uptake, demographic details,	Within four months of introducing MVA, it was possible to replace one of three weekly theatre lists with MVA. The saving on the theatre recharge was £ 92,000 per annum. The costs for setting up and running the MVA sessions amounted to £ 28,000 per annum. However, the

Author, year [country]	Aim/objective(s)	Population	Study type	Summary of main findings
	conscious first-trimester termination of pregnancy within the [NHS] service and for the population	NHS facility	timing, pain score, complications, contraceptive uptake, and economic implications for the service	procedure room containing the scanner, the electric gynaecology couch and a mobile light were already in use by the service and were more fully utilized once the MVA sessions commenced. There were additional setup costs for purchase of dilator sets and theatre clothing. Overall, the difference in cost of the weekly theatre list against running two MVA sessions per week resulted in an annual saving of around £6 0,000.
(Prada, Maddow-Zimet et al. 2013) [Colombia]	(1) To estimate the costs incurred by health care facilities in treating complications of unsafe abortion<br><br>(2) To estimate the total annual cost to the health system of providing PAC<br><br>(3) To compare the cost of treating complications of unsafe abortion with the cost of providing legal abortion services, and explore some of the factors driving these differences	Estimates based on a sample of facilities	Estimation	The median direct cost of treating a woman with abortion complications ranged from US$ 44—141. A legal abortion at a secondary or tertiary facility was costly (medians, $213 and $189, respectively), in part because of the use of D&C as well as because of administrative barriers. At specialized facilities, where MVA and medication abortion are used, the median cost of provision was much lower ($45). Provision of PAC and legal abortion services at higher-level facilities results in unnecessarily high health care costs. These costs can be reduced significantly by providing services in a timely fashion at primary-level facilities and by using safe, non-invasive, and less costly abortion methods.
(Ramos and Rios 2015) [Peru]	To explore debates of whether a woman with an incomplete abortion should be treated with surgical procedures or medical procedures	Debates on the topic of MVA versus MA in Peru	Literature review	Treating incomplete abortion has substantial costs for the health system. MVA is cheaper than EVA.
(Robson, Kelly et al. 2009) [United Kingdom]	To determine the acceptability, efficacy and costs of medical TOP compared with surgical TOP at less than 14 weeks' gestation, and to understand women's decision-making processes and experiences when accessing the termination service	Women accepted for TOP with pregnancies <14 weeks' gestation on the day of abortion	A partially randomised preference trial and economic evaluation with follow-up at two weeks and three months	Surgical TOP cost more than medical TOP due to higher inpatient standard costs. Although complication rates were higher with medical TOP, it was still more cost effective. Mean cost by procedure type (excluding out-of-pocket costs) are as follows: legal, MA in clinic was $165 with complications and $132 without complications; legal MVA in clinic was $223 with complications and $197 without complications; legal MVA in hospitals was $467 with complications and $374 without complications; MVA PAC in hospitals was $563 with complications and $231 without complications; D&C in hospitals was $821 with complications and $657 without complications; D&C PAC in hospitals was $2,301 with complications and $458 without complications.
(Rodriguez, Mendoza	To compare the costs to the	Women seeking abortion	Cost analysis	For every 1,000 women receiving PAC instead of legal abortion, 16

Author, year [country]	Aim/objective(s)	Population	Study type	Summary of main findings
et al. 2015) [Colombia]	health system of three approaches to the provision of abortion care in Colombia: PAC for complications of unsafe abortions, and for legal abortions in a health facility, misoprostol-only MA and vacuum aspiration abortion	care at three high-volume facilities in Columbia (n=1,411)		women had unnecessary complications at a cost of US$ 48,000. For legal abortion, both misoprostol and MVA were less costly than D&C for both complicated and uncomplicated cases. For uncomplicated PAC, the average cost for an MVA was nearly half the cost of a D&C at the same type of facility ($ 231 versus $ 458). Assuming a 9% spontaneous abortion rate, and if the remainder of PAC cases currently observed were replaced with legal abortion (medical or MVA), the health system would save an additional $ 163,000 and prevent 16 complications per 1,000 abortions. Additionally, the health system could save $177,000 (per 1,000 women) from baseline by replacing D&C with MVA.
(Sethe and Murdoch 2013) [United Kingdom]	To determine whether abortion or in-vitro fertilization (IVF) treatment has the greater 'regulatory burden' the authors consider the impact of the law, licensing, inspection, amount of paperwork and reporting requirements, the reception by practitioners and costs on these two clinical procedures	Anyone considering abortion or IVF in the United Kingdom	Literature review	Independent IVF and abortion clinics pay a fee to the Care Quality Commission of £ 2,505—5,705. NHS hospital fees are based on bed number, ranging from £ 15,000—75,000. This fee applies to all activities within a Trust, within which both IVF and abortion services will only be a small factor.
(Sharma and Guthrie 2006) [United Kingdom]	To find ways of improving access to earlier and safer abortion, and to offer women more choice of method, by assessing the feasibility of (1) a telephone booking clinic and (2) a local anaesthetic outpatient surgical termination of pregnancy service<br><br>To assess whether mainstream implementation of these changes could be achieved at no extra cost	Females seeking an abortion at the Women and Children's Hospital, Hull Royal Infirmary	Descriptive study of two pilot projects<br><br>Cross-sectional survey	Cost of a medical TOP or a surgical TOP (day case) ranges from £ 462—578 per patient. The trial outpatient local anaesthetic outpatient surgical termination of pregnancy clinic cost £ 366 per patient. The present cost per outpatient consultation of £ 217 per patient could be reduced to £ 177 if the nurse telephone clinic was used.
(Shearer, Walker et al. 2010) [global]	To evaluate the quality of costing studies of PAC from low- and middle-income countries and to describe costs in various settings	Facilities in different countries that provide PAC	Regression analysis and systematic review	Data indicate that the cost (in 2007 international dollars) of PAC in Africa and Latin America is $ 392 and $ 430, respectively, per case. Differences in PAC costs were associated with region, procedure, facility level, and case severity.
(Sheldon and	To provide a detailed	Medically-trained abortion	Reassessment of	Allowing nurses to provide vacuum aspiration for induced abortion

Author, year [country]	Aim/objective(s)	Population	Study type	Summary of main findings
Fletcher 2017) [United Kingdom]	reassessment of the relevant law (on doctors performing MVA procedures) and the clinical evidence that supports this assumption	providers (doctors, nurses, midwives)	relevant law and clinical evidence that supports the law's assumption	potentially offers a more sustainable and economically efficient basis for the long-term development of excellent care. It would free up doctors to focus on those aspects of service provision where their specific expertise is needed.
(Singh 2010) [global]	To review the scientific evidence on the consequences of unsafe abortion, highlight gaps in the evidence base, suggest areas where future research efforts are needed, and speculate on the future situation regarding consequences and evidence over the next 5–10 years	Women who obtained unsafe abortions globally	Literature review	Prior to 2005, studies examining cost of providing PAC included supplies, drugs, labour, and overhead or health systems costs. As a group, these studies demonstrate that providing PAC is a costly undertaking for the patient, for the hospital, and for the health system as a whole. When comparing the cost of MVA to the cost of D&C, MVA is a more cost effective and safer treatment than D&C.
(Sjostrom, Kopp Kallner et al. 2016) [Sweden]	To calculate the cost-effectiveness of early MA performed by nurse-midwifes in comparison to physicians in a high resource setting where ultrasound dating is part of the protocol	Healthy women seeking abortion services at an outpatient clinic of a university hospital in Sweden (n=85)	Cost effectiveness analysis	The direct costs of the woman's first visit to the clinic of the standard treatment was € 58.3 per procedure, and the cost of the intervention treatment was € 45. The physician providers' consultations lasted 14 minutes on average. Less experienced physicians consulted more often than more senior physicians. Nurse-midwife consultations lasted six minutes on average.
(Sundari Ravindran and Fonn 2011) [global]	To review studies relating to social franchising initiatives and the impact these have had on reproductive health services	45 clinical social franchises across 27 countries	Review	Franchises that reported out-of-pocket payment as the sole source of payment included 19 that specifically targeted low-income groups. The Ghana Blue Star franchise recommends charging US$ 25-40 for abortion services, in the context of women earning on average US$ 33 a month.
(Tewari, Pirwany et al. 1995) [United Kingdom]	To compare the costs of two regimes of mid-trimester medical termination of pregnancy	Women seeking abortion care at a district general maternity hospital	A prospective observational study and cost comparison	Median cost of TOP was £ 420 in the mifepristone group vs. £ 885 in the PGE2 group ($p<0.0001$). Pre-treatment with mifepristone in second trimester termination of pregnancy results in substantial savings in the cost of treatment.
(Thapa, Neupana et al. 2012) [Nepal]	To compare abortion service users between 2005 and 2010 in Nepal	People seeking surgical abortions from the Maternity Hospital abortion clinic, Nepal, in 2005 (n=672) and 2010 (n=392)	Comparison of two rounds of cross-sectional surveys	The cost incurred by the clinic providing abortion services decreased from Rs 971 (US$ 13.20) in 2005 to Rs 796 (US$ 10.96), reflecting an increase in clients. The revenues from user fees offset the clinics expenditures on abortion services.
(Thomas, Paranjothy et al. 2003) [United Kingdom]	To describe the outcome of a National Audit of Clinical Practices in Induced Abortion	Abortion care providers in England and Wales (n=240)	Review of an audit conducted via a postal survey	While manufacturers recommend a dose of 600 mg for medical abortions up to 9 weeks, randomized controlled trials indicated that a 200mg dose is as effective. Each 200mg dose costs £ 14. The conventionally used prostaglandin E1 analog is gemeprost, which costs

Author, year [country]	Aim/objective(s)	Population	Study type	Summary of main findings
				£ 20 per 1mg pessary. The alternative, misoprostol, costs £ 1 per dose.
(Tunc 2008) [United States]	To review the vacuum aspirator's history and answer why, in less than a decade, electric vacuum suction became American physicians' abortion technology of choice	Review of vacuum aspirator's history in the United States	Historical review	When New York State's abortion laws were liberalized in 1970, Dr. Nathanson, who had vowed to make abortion safe and legal, bought a Berkeley apparatus for his clinic that was the largest legal abortion clinic in the western world. Nathanson maintained that the Berkeley suction unit was inexpensive, much cleaner, more reliable, and more suitable for the new outpatient setting than D&C. As the success spread, other aspirator manufacturers began to appear, and the technology entered mainstream medicine. Because it could be used with any aspiration apparatus and then simply discarded, the Karman cannula also reduced sterilization costs and the risk of infection and cross-contamination between patient.
(Tupper, Speed Andrews et al. 2007) [United Kingdom]	To report on setting up and running a new outpatient service for early medical termination under seven weeks gestation	Women seeking abortion care at an outpatient service for early medical termination	Cost analysis	Estimated cost per case in the first year was £ 156, representing a considerable cost saving compared to £ 498 for surgical termination and £423 for inpatient medical termination.
(Upadhyay, Cartwright et al. 2018) [United States]	To assess medication abortion access among California's public university students. Specifically, this study estimated current medication abortion use and travel time, costs, and appointment availability at the abortion facilities closest to each of the 34 campuses	California's 34 public university students and the 152 abortion-providing facilities in California	Projections and "mystery shopper" calls	Almost all facilities closest to campus accepted state Medicaid; one did not, but the next closest facility was only .05 miles further away.
(Upadhyay, Johns et al. 2018) [United States]	To assess the incidence of abortion-related emergency department visits in the United States by estimating the proportion of visits that were abortion-related and described the characteristics of patients making these visits, the diagnoses and subsequent treatments received by these patients, the sociodemographic and hospital characteristics associated with the incidents and observation care	Data from the Nationwide Emergency Department Sample on women seeking emergency care for abortion-related issues (n=27,941)	Retrospective observational study	Average emergency department costs were $ 4,719, with 8.6% of emergency department visits costing $ 10,000 or more.

Author, year [country]	Aim/objective(s)	Population	Study type	Summary of main findings
	only, and the rate of major incidents for all abortion patients			
(Visaria, Barua et al. 2008) [India]	To understand gynaecologists' perspectives on MA and chemists' understanding of marketing strategies and drug distribution	Gynaecologists and chemists in 2004 in the Ahmedabad urban area	Exploratory qualitative study using interviews	The cost of mifepristone tablets ranged from rp 325—930. Misoprostol costs ranged from rp 31—60. The total cost of MA was estimated to be rp 1,200 including cost of three ultrasounds. Service providers believed that cost of surgical abortion at a government facility would be substantially less than this amount.
(Vlassoff, Walker et al. 2009) [global]	To estimate the health system costs of PAC in Africa and Latin America	Women seeking PAC services and women hospitalized for serious medical complications of induced abortion in Africa and Latin America	Cost estimations	Cost-per-case averages, including overhead and capital costs, yielded averages of $ 114 for Africa and $ 130 for Latin America. The overall costs of PAC per patient using the WHO Mother-Baby Package model showed considerable variability across the five countries, from $ 10—112 (in 2006 US$) for actual practice, and from $31—212 for care based on WHO standards. Costs in Latin America were substantially higher than those in Sub- Saharan Africa—$ 109 vs. $ 57 for actual practice, and $ 172 vs. $ 65 for WHO-recommended care—primarily reflecting higher Latin American salaries.
(Vlassoff, Musange et al. 2015) [Rwanda]	To estimate the cost of PAC to the national health-care system disaggregated by health-care level, region, severity of complication, and cost component	Randomly selected public and private health facilities (n=39) representing three levels of health care across all five regions	Cost estimation	The average annual PAC cost per client, across five types of abortion complications, was $ 93 and was directly related to the level of care: treatment of postabortion cases at referral hospitals was the most costly ($ 239 on average); per-case PAC treatment at district hospitals cost $ 93, while $ 72 was spent per case at health centres. The total cost of PAC nationally was estimated to be $ 1.7 million per year, 49% of which was expended on direct non-medical costs. Satisfying all demands for PAC would raise the national cost to $ 2.5 million per year. Expenditure at district hospitals was only a little over half that at referral hospitals; health centres spent about one-quarter as much. Shock was the most costly complication ($ 53 per treatment), whereas cervical and vaginal lacerations were the least expensive ($12).
(Vlassoff, Singh et al. 2016) [Ethiopia, Uganda, Rwanda and Colombia]	To expand the research findings of these four comprehensive national surveys of the cost of PAC to national health systems in Ethiopia, Uganda, Rwanda, and Colombia and to make use of their extensive datasets	Costing studies from Ethiopia, Uganda, Rwanda, and Colombia	4-country comparative costing study	Labour cost varies widely: in Ethiopia and Colombia doctors spend about 30–60% more time with PAC patients than do nurses; in Uganda and Rwanda an opposite pattern is found. Labour costs range from I$ 42.80 in Uganda to I$ 301.30 in Colombia. The cost of drugs and supplies does not vary greatly, ranging from I$ 79 in Colombia to I$ 115 in Rwanda. Capital and overhead costs are substantial, amounting to 52–68% of total PAC costs. Total costs per PAC case vary from I$ 334 in Rwanda to I$ 972 in Colombia. The financial burden of PAC is considerable: the expense of treating each PAC case is equivalent to around 35% of annual per capita income in Uganda, 29% in Rwanda,

Author, year [country]	Aim/objective(s)	Population	Study type	Summary of main findings
				and 11% in Colombia.
(White, Adams et al. 2019) [United States]	To compare pregnancy options counselling and referral practices at state- and Title X-funded family planning organizations in Texas after enforcement of a policy restricting abortion referrals for providers participating in state-funded programs, which differed from Title X guidelines to provide referrals for services upon request	Publicly funded family planning organizations in Texas	Qualitative: semi-structured interviews	Some respondents advised women that not all facilities offered medication and surgical abortion; others suggested that women call to inquire about the cost of the procedure and funding available to help them cover their expenses. Respondents noted that the limited number of local facilities made it challenging to provide information. While respondents rarely mentioned informing women about how they might cover the cost of abortion care or locations where different abortions methods were offered, respondents were willing to facilitate women's access to prenatal care. This difference may reflect that providers know less about abortion than prenatal services or do not feel comfortable discussing abortion.
(Winikoff, Hassoun et al. 2011) [United States and France]	To examine the commercial, political, regulatory, and legislative history of the introduction of mifepristone misoprostol in France and the United States	Different subgroups across the United States and France, such as researchers, non-profits, government, and women seeking services	Historical narrative	The insurance situation in the United States has important implications for the use and accessibility of mifepristone. On the one hand, because few women use insurance to cover the procedure, insurance companies exert little influence over practice patterns or standards of care compared to other surgical or reproductive health procedures. As a result, clinics and providers have been free to develop innovative service models for provision of the service that ultimately may have reduced the overall cost of the procedure. On the other hand, the price of the procedure has a wide range. Studies have found that the adjusted cost of providing MA care varies significantly depending upon the practice model used (from $252—460 per abortion, median $351). Consequently, the method may be more or less accessible or a more or less attractive alternative to surgical abortion depending upon the practice and pricing model in place.
(Wu, Maru et al. 2017) [Nepal]	To review abortion care in Nepal 15 years after it was legalized	This paper reviews abortion care in relation to practitioners, women seeking abortion services, national policies, etc., from the national to the individual level	Review of current status of abortion in Nepal	While the 2009 Supreme Court decision established the legal framework for the government to mandate free and accessible abortion services in the public sector, there was no policy to implement safe abortion services until the passage of the Safe Abortion Service Guidelines of 2016. Under these guidelines, all government facilities should provide free abortion services. However, the provider reimbursement scheme outlined in the guidelines is less profitable for providers than it was when women paid out of pocket. It remains to be seen whether these new guidelines thus create monetary incentives that encourage providers to shift abortion provision from the public to the private sector, thereby adversely affecting access at public facilities.

Author, year [country]	Aim/objective(s)	Population	Study type	Summary of main findings
(Xia, She et al. 2011) [China]	To compare the clinical efficacy, complications, side effects and direct costs of the two abortion methods for gestation up to 49 days in Guangzhou	Women seeking abortion up to 49 days gestation at the out-patient department of the Department of Obstetrics and Gynecology, First Municipal Hospital of Guangzhou (n=213 women receiving MA; n=21 women receiving surgical abortion)	Economic evaluation (cost-minimization) from a third-party payer's perspective	Accounting only for initial costs, surgical abortion appeared to be 32% more expensive than MA (CNY 367.56 + 21.31 vs. CNY 279.25 + 9.48). However, when the subsequent costs of examination and treatment due to incomplete abortion and bleeding within the period of 2-week follow-up were added, on average the medical group incurred almost equivalent costs as the surgical group (CNY 379.03 + 27.75 vs. CNY 375.16 + 12.81).

References

Aantjes, C. J., A. Gilmoor, E. V. Syurina and T. L. Crankshaw (2018). "The status of provision of post abortion care services for women and girls in Eastern and Southern Africa: a systematic review." Contraception **98**(2): 77-88.

Acosta de Hart, H., C. Umaña and C. Villarreal (2002). "Oriéntame: Preventing and Solving Problems Related to Unwanted Pregnancy for 25 Years in Colombia." Reproductive Health Matters **10**(19): 138-142.

Afable-Munsuz, A., H. Gould, F. Stewart, K. A. Phillips, S. L. Van Bebber and C. Moore (2007). "Provider practice models for and costs of delivering medication abortion — evidence from 11 US abortion care settings." Contraception **75**(1): 45-51.

Anjum, Z. K. (2000). "Termination of early pregnancy with a reduced oral dose of mifepristone and vaginal misoprostol." S Afr Med J **90**(9): 889-891.

Ayanore, M. A., M. Pavlova, R. Biesma and W. Groot (2017). "Stakeholder's experiences, expectations and decision making on reproductive care: An ethnographic study of three districts in northern Ghana." PLoS One **12**(11): e0186908.

Babigumira, J. B., A. Stergachis, D. L. Veenstra, J. S. Gardner, J. Ngonzi, P. Mukasa-Kivunike and L. P. Garrison (2011). "Estimating the costs of induced abortion in Uganda: a model-based analysis." BMC Public Health **11**(1): 904-904.

Baird, B. (2015). "Medical abortion in Australia: a short history." Reproductive Health Matters **23**(46): 169-176.

Baird, B. (2017). "Decriminalization and Women's Access to Abortion in Australia." Health & Human Rights: An International Journal **19**(1): 197-208.

Battistelli, M. F., S. Magnusson, M. A. Biggs and L. Freedman (2018). "Expanding the Abortion Provider Workforce: A Qualitative Study of Organizations Implementing a New California Policy." Perspectives on Sexual & Reproductive Health **50**(1): 33-39.

Baxerres, C., I. Boko, A. Konkobo, F. Ouattara and A. Guillaume (2018). "Abortion in two francophone African countries: a study of whether women have begun to use misoprostol in Benin and Burkina Faso." Contraception **97**(2): 130-136.

Belton, S. and A. Whittaker (2007). "Kathy Pan, sticks and pummelling: Techniques used to induce abortion by Burmese women on the Thai border." Social Science & Medicine **65**(7): 1512-1523.

Benson, J., H. Gebreselassie, M. A. Manibo, K. Raisanen, H. B. Johnston, C. Mhango and B. A. Levandowski (2015). "Costs of postabortion care in public sector health facilities in Malawi: a cross-sectional survey." BMC Health Serv Res **15**: 562.

Benson, J., L. A. Nicholson, L. Gaffikin and S. N. Kinoti (1996). "Complications of unsafe abortion in sub-Saharan Africa: a review." Health Policy Plan **11**(2): 117-131.

Benson, J., M. Okoh, K. KrennHrubec, M. A. Lazzarino and H. B. Johnston (2012). "Public hospital costs of treatment of abortion complications in Nigeria." Int J Gynaecol Obstet **118 Suppl 2**: S134-140.

Berer, M. (2000). "Making abortions safe: a matter of good public health policy and practice." Bull World Health Organ **78**(5): 580-592.

Berer, M. (2005). "Medical abortion: issues of choice and acceptability." Reproductive Health Matters **13**(26): 25-34.

Betala Belinga, J. F., A. Valence, A. Zaccabri and J. Fresson (2010). "[Voluntary abortion cost in France and prospective payment system: to raise the issue of so many misstatements]." J Gynecol Obstet Biol Reprod (Paris) **39**(7): 549-553.

Billings, D. L. and J. Benson (2005). "Postabortion care in Latin America: policy and service recommendations from a decade of operations research." Health Policy Plan **20**(3): 158-166.

Brown, R. W. and R. T. Jewell (1996). "The Impact of Provider Availability on Abortion Demand." Contemporary Economic Policy **14**(2): 95-106.

Cano, J. K. and A. M. Foster (2016). ""They made me go through like weeks of appointments and everything": Documenting women's experiences seeking abortion care in Yukon territory, Canada." Contraception **94**(5): 489-495.

Chełstowska, A. (2011). "Stigmatisation and commercialisation of abortion services in Poland: turning sin into gold." Reproductive Health Matters **19**(37): 98-106.

Choobun, T., S. Khanuengkitkong and S. Pinjaroen (2012). "A comparative study of cost of care and duration of management for first-trimester abortion with manual vacuum aspiration (MVA) and sharp curettage." Archives of Gynecology & Obstetrics **286**(5): 1161-1164.

Coast, E. and S. F. Murray (2016). "“These things are dangerous”: Understanding induced abortion trajectories in urban Zambia." Social Science & Medicine **153**: 201-209.

Cockrill, K. and T. A. Weitz (2010). "Abortion patients' perceptions of abortion regulation." Womens Health Issues **20**(1): 12-19.

Colman, S. and T. Joyce (2011). "Regulating Abortion: Impact on Patients and Providers in Texas." Journal of Policy Analysis and Management **30**(4): 775-797.

Contreras, X., M. G. van Dijk, T. Sanchez and P. S. Smith (2011). "Experiences and Opinions of Health-Care Professionals Regarding Legal Abortion in Mexico City: A Qualitative Study." Studies in Family Planning **42**(3): 183-190.

Costa, S. H. (1998). "Commercial availability of misoprostol and induced abortion in Brazil." International Journal of Gynecology & Obstetrics **63**: S131-S139.

Couteau, C., C. D'Ercole, F. Bretelle, L. Boubli, B. Guidicelli and C. Chau (2016). "Methods of induction of labor in termination of pregnancy after 22 weeks: About 3 procedures." Journal De Gynecologie Obstetrique Et Biologie De La Reproduction **45**(6): 652-658.

Creinin, M. D. (2000). "Randomized comparison of efficacy, acceptability and cost of medical versus surgical abortion." Contraception **62**(3): 117-124.

Creinin, M. D., E. Shore, S. Balasubramanian and B. Harwood (2005). "The true cost differential between mifepristone and misoprostol and misoprostol-alone regimens for medical abortion." Contraception **71**(1): 26-30.

Crighton, E. and M. Ebert (2002). "RU 486 and abortion practices in Europe: From legalization to access." Women & Politics **24**(3): 13-33.

Dawson, A., D. Bateson, J. Estoesta and E. Sullivan (2016). "Towards comprehensive early abortion service delivery in high income countries: insights for improving universal access to abortion in Australia." BMC Health Services Research **16**: 612-612.

Dennis, A., R. Manski and K. Blanchard (2014). "Does Medicaid Coverage Matter? A Qualitative Multi-State Study of Abortion Affordability for Low-income Women." Journal of Health Care for the Poor & Underserved **25**(4): 1571-1585.

Díaz-Olavarrieta, C., V. M. Cravioto, A. Villalobos, N. Deeb-Sossa, L. García and S. G. García (2012). "Mexico City's Legal Abortion Program: health workers' experiences." Revista Panamericana de Salud Publica **32**(6): 399-404.

Dobie, S. A., L. Gober and R. A. Rosenblatt (1998). "Family planning service provision in rural areas: a survey in Washington State." Fam Plann Perspect **30**(3): 139-142, 147.

Doran, F. and J. Hornibrook (2014). "Rural New South Wales women's access to abortion services: highlights from an exploratory qualitative study." Aust J Rural Health **22**(3): 121-126.

Duggal, R. (2004). "The political economy of abortion in India: cost and expenditure patterns." Reproductive Health Matters **12**: 130-137.

Dzuba, I. G., B. Winikoff and M. Pena (2013). "Medical abortion: a path to safe, high-quality abortion care in Latin America and the Caribbean." Eur J Contracept Reprod Health Care **18**(6): 441-450.

Ely, G. E., T. W. Hales and D. L. Jackson (2018). "A cross-cultural exploration of abortion fund patients in the USA and the Republic of Ireland, Northern Ireland and the Isle of Man." Culture, Health & Sexuality **20**(5): 560-573.

Esia-Donkon, K., E. K. M. Darteh, H. Blemano and H. Asare (2015). "Who Cares? Pre and Post Abortion Experiences among Young Females in Cape Coast Metropolis, Ghana." African Journal of Reproductive Health / La Revue Africaine de la Santé Reproductive **19**(2): 43-51.

Font-Ribera, L., G. Perez, A. Espelt, J. Salvador and C. Borrell (2009). "Determinants of induced abortion delay." Gaceta Sanitaria **23**(5): 415-419.

Foster, D. G. and K. Kimport (2013). "Who Seeks Abortions at or After 20 Weeks?" Perspectives on Sexual & Reproductive Health **45**(4): 210-218.

Foy, R., G. C. Penney, J. M. Grimshaw, C. R. Ramsay, A. E. Walker, G. MacLennan, S. C. Stearns, L. McKenzie and A. Glasier (2004). "A randomised controlled trial of a tailored multifaceted strategy to promote implementation of a clinical guideline on induced abortion care." Bjog **111**(7): 726-733.

Gallo, M. F. and N. C. Nghia (2007). "Real life is different: a qualitative study of why women delay abortion until the second trimester in Vietnam." Social Science & Medicine **64**(9): 1812-1822.

Gan, K., Y. Zhang, X. Jiang, Y. Meng, L. Hou and Y. Cheng (2011). "Perspectives of Chinese healthcare providers on medical abortion." International Journal of Gynecology & Obstetrics **114**(1): 15-17.

Gibb, S., C. Donaldson and R. Henshaw (1998). "Assessing strength of preference for abortion method using 'willingness to pay': a useful research technique for measuring values." Journal of Advanced Nursing **27**(1): 30-36.

Glenton, C., A. M. Sorhaindo, B. Ganatra and S. Lewin (2017). "Implementation considerations when expanding health worker roles to include safe abortion care: a five-country case study synthesis." BMC Public Health **17**: 1-13.

Graff, M. and D. A. Amoyaw (2009). "Barriers to Sustainable MVA Supply in Ghana: Challenges for the Low-Volume, Low-Income Providers." African Journal of Reproductive Health / La Revue Africaine de la Santé Reproductive **13**(4): 73-80.

Gresh, A. and P. Maharaj (2011). "A qualitative assessment of the acceptability and potential demand for medical abortion among university students in Durban, South Africa." Eur J Contracept Reprod Health Care **16**(2): 67-75.

Grossman, D., S. Baum, L. Fuentes, K. White, K. Hopkins, A. Stevenson and J. E. Potter (2014). "Change in abortion services after implementation of a restrictive law in Texas." Contraception **90**(5): 496-501.

Grossman, D., C. Ellertson, D. A. Grimes and D. Walker (2004). "Routine follow-up visits after first-trimester induced abortion." Obstet Gynecol **103**(4): 738-745.

Guttmacher, S., F. Kapadia, N. Jim Te Water and H. de Pinho (1998). "Abortion Reform in South Africa: A Case Study of the 1996 Choice on Termination of Pregnancy Act." International Family Planning Perspectives **24**(4): 191-194.

Harries, J., N. Lince, D. Constant, A. Hargey and D. Grossman (2012). "The challenges of offering public second trimester abortion services in South Africa: health care providers' perspectives." Journal of Biosocial Science **44**(2): 197-208.

Harvey, N. and M. Gaudoin (2005). "Effectiveness of a nurse-led pregnancy termination clinic." Nursing Times **101**(17): 34-36.

Henshaw, R. C., S. A. Naji, I. T. Russell and A. A. Templeton (1994). "A prospective economic evaluation comparing medical abortion (using mifepristone and gemeprost) and surgical vacuum aspiration." British Journal of Family Planning **20**(3): 64-68.

Henshaw, S. K. (1995). "Factors hindering access to abortion services." Family Planning Perspectives **27**(2): 54-87.

Henshaw, S. K., I. Adewole, S. Singh, B. Oye-Adeniran, R. Hussain and A. Bankole (2008). "Severity and cost of unsafe abortion complications treated in Nigerian hospitals." International Family Planning Perspectives **34**(1): 40-50.

Henshaw, S. K. and L. B. Finer (2003). "The accessibility of abortion services in the United States, 2001." Perspectives on Sexual & Reproductive Health **35**(1): 16-24.

Hollander, D. (1995). "Mifepristone and Vaginal Misoprostol are Effective, Acceptable and Inexpensive Medical Abortion Regimen." Family Planning Perspectives **27**(5): 223-224.

Htay, T. T., J. Sauvarin and S. Khan (2003). "Integration of Post-Abortion Care: The Role of Township Medical Officers and Midwives in Myanmar." Reproductive Health Matters **11**(21): 27-36.

Hu, D., D. Grossman, C. Levin, K. Blanchard, R. Adanu and S. J. Goldie (2010). "Cost-effectiveness analysis of unsafe abortion and alternative first-trimester pregnancy termination strategies in nigeria and ghana." African Journal of Reproductive Health **14**(2): 85-103.

Hu, D., D. Grossman, C. Levin, K. Blanchard and S. Goldie (2009). "Cost-effectiveness analysis of alternative first-trimester pregnancy termination strategies in Mexico City." BJOG: An International Journal of Obstetrics & Gynaecology **116**(6): 768-779.

Ilboudo, P. G. C., G. Greco, J. Sundby and G. Torsvik (2016). "Estimating the costs for the treatment of abortion complications in two public referral hospitals: a cross-sectional study in Ouagadougou, Burkina Faso." BMC Health Services Research **16**: 1-10.

Jerman, J. and R. K. Jones (2014). "Secondary Measures of Access to Abortion Services in the United States, 2011 and 2012: Gestational Age Limits, Cost, and Harassment." Women's Health Issues **24**(4): e419-e424.

Johnston, H. B., S. Akhter and E. Oliveras (2012). "Quality and efficiency of care for complications of unsafe abortion: a case study from Bangladesh." International Journal of Gynecology & Obstetrics **118**: S141-147.

Johnston, H. B., M. F. Gallo, J. Benson, H. B. Johnston, M. F. Gallo and J. Benson (2007). "Reducing the costs to health systems of unsafe abortion: a comparison of four strategies." Journal of Family Planning & Reproductive Health Care **33**(4): 250-257.

Johnston, H. B., E. Oliveras, S. Akhter and D. G. Walker (2010). "Health System Costs of Menstrual Regulation and Care For Abortion Complications in Bangladesh." International Perspectives on Sexual & Reproductive Health **36**(4): 197-201.

Jones, B. S. and T. A. Weitz (2009). "Legal barriers to second-trimester abortion provision and public health consequences." American Journal of Public Health **99**(4): 623-630.

Jones, R. K., M. Ingerick and J. Jerman (2018). "Differences in Abortion Service Delivery in Hostile, Middle-ground, and Supportive States in 2014." Women's Health Issues **28**(3): 212-218.

Kacanek, D., A. Dennis, K. Miller and K. Blanchard (2010). "Medicaid funding for abortion: providers' experiences with cases involving rape, incest and life endangerment." Perspectives on Sexual & Reproductive Health **42**(2): 79-86.

Kamali, P., M. Hohmann, J. Herrero and W. Kunzel (1998). "[Gameprost, sulproston and dinoproston for induced abortion in the 15th-24th week of pregnancy]." Zentralbl Gynakol **120**(6): 293-300.

Koontz, S. L., O. Molina de Perez, K. Leon and A. Foster-Rosales (2003). "Treating incomplete abortion in El Salvador: cost savings with manual vacuum aspiration." Contraception **68**(5): 345-351.

Lalley, J. J., R. D. Jelsema, P. S. Huzel, S. B. Ransom and R. J. Sokol (2001). "The cost-effectiveness of misoprostol versus prostaglandin E2 for second trimester termination of pregnancy." Clinical Journal of Women's Health **1**(3): 149-156.

Levin, C., D. Grossman, K. Berdichevsky, C. Diaz, B. Aracena, S. G. Garcia and L. Goodyear (2009). "Exploring the costs and economic consequences of unsafe abortion in Mexico City before legalisation." Reproductive Health Matters **17**(33): 120-132.

Li, C. L., L. P. Song, S. Y. Tang, L. Zhou, H. He, X. T. Mo and Y. M. Liao (2017). "Efficacy, Safety, and Acceptability of Low-Dose Mifepristone and Self-Administered Misoprostol for Ultra-Early Medical Abortion: A Randomized Controlled Trial." Reprod Sci **24**(5): 731-737.

Limacher, J. J., I. Daniel, S. Isaacksz, G. J. Payne, S. Dunn, P. C. Coyte and A. Laporte (2006). "Early Abortion in Ontario: Options and Costs." Journal of Obstetrics and Gynaecology Canada **28**(2): 142-148.

Lince-Deroche, N., D. Constant, J. Harries, K. Blanchard, E. Sinanovic and D. Grossman (2015). "The costs of accessing abortion in South Africa: women's costs associated with second-trimester abortion services in Western Cape Province." Contraception **92**(4): 339-344.

Lince-Deroche, N., D. Constant, J. Harries, J. Kluge, K. Blanchard, E. Sinanovic and D. Grossman (2018). "The costs and cost effectiveness of providing second-trimester medical and surgical safe abortion services in Western Cape Province, South Africa." PLoS One **13**(6): e0197485.

Lince-Deroche, N., T. Fetters, E. Sinanovic, J. Devjee, J. Moodley and K. Blanchard (2017b). "The costs and cost effectiveness of providing first-trimester, medical and surgical safe abortion services in KwaZulu-Natal Province, South Africa." PLoS One **12**(4): e0174615.

Magotti, R. F., P. G. Munjinja, R. S. Lema and E. K. Ngwalle (1995). "Cost-effectiveness of managing abortions: manual vacuum aspiration (MVA) compared to evacuation by curettage in Tanzania." East Afr Med J **72**(4): 248-251.

Manouana, M., P. Kadhel, A. Koffi and E. Janky (2013). "Avortements illégaux par le misoprostol en Guadeloupe." Journal de Gynécologie Obstétrique et Biologie de la Reproduction **42**(2): 137-142.

Medoff, M. H. (2008b). "The Spillover Effects of Restrictive Abortion Laws." Gender Issues **25**(1): 1-10.

Messinger, C. J., I. Mahmud, S. Kanan, Y. T. Jahangir, M. Sarker and S. F. Rashid (2017). "Utilization of mobile phones for accessing menstrual regulation services among low-income women in Bangladesh: a qualitative analysis." Reproductive Health **14**: 1-11.

Murthy, A. and M. D. Creinin (2003). "Pharmacoeconomics of medical abortion: a review of cost in the United States, Europe and Asia." Expert Opin Pharmacother **4**(4): 503-513.

Ordinioha, B. and S. Brisibe (2008). "Clandestine abortion in Port Harcourt: providers' motivations and experiences." Niger J Med **17**(3): 291-295.

Parmar, D., T. Leone, E. Coast, S. F. Murray, E. Hukin and B. Vwalika (2017). "Cost of abortions in Zambia: A comparison of safe abortion and post abortion care." Global Public Health **12**(2): 236-249.

Payne, C. M., M. P. Debbink, E. A. Steele, C. T. Buck, L. A. Martin, J. A. Hassinger and L. H. Harris (2013). "Why Women are dying from unsafe Abortion: Narratives of Ghanaian abortion providers." African Journal of Reproductive Health **17**(2): 118-128.

Penney, G. C., L. McKessock, R. Rispin, H. Elrefaey and A. Templeton (1995). "AN EFFECTIVE, LOW-COST REGIMEN FOR EARLY MEDICAL ABORTION." British Journal of Family Planning **21**(1): 5-6.

Pheterson, G. and Y. Azize (2005). "Abortion Practice in the Northeast Caribbean: “Just write down stomach pain”." Reproductive Health Matters **13**(26): 44-53.

Pillai, M., V. Welsh, K. Sedgeman, A. C. Gazet, J. Staddon and H. Carter (2015). "Introduction of a manual vacuum aspiration service: a model of service within a NHS Sexual Health Service." Journal of Family Planning & Reproductive Health Care **41**(1): 27-32.

Prada, E., I. Maddow-Zimet and F. Juarez (2013). "The Cost of Postabortion Care and Legal Abortion In Colombia." International Perspectives on Sexual & Reproductive Health **39**(3): 114-123.

Ramos, M. G. and E. G. Rios (2015). "Controversies in the treatment of incomplete abortion, MVA versus medical treatment with misoprostol." Revista Peruana De Ginecologia Y Obstetricia **61**(1): 57-64.

Robson, S., T. Kelly, D. Howel, M. Deverill, J. Hewison, M. Lie, E. Stamp, N. Armstrong and C. May (2009). "Randomised preference trial of medical versus surgical termination of pregnancy less than 14 weeks' gestation (TOPS)." Health Technology Assessment **13**(37): 1-148.

Rodriguez, M. I., W. S. Mendoza, C. Guerra-Palacio, N. A. Guzman and J. E. Tolosa (2015). "Medical abortion and manual vacuum aspiration for legal abortion protect women's health and reduce costs to the health system: findings from Colombia." Reproductive Health Matters **22**: 125-133.

Sethe, S. and A. Murdoch (2013). "Comparing the Burden: What Can We Learn by Comparing Regulatory Frameworks in Abortion and Fertility Services?" Health Care Analysis **21**(4): 338-354.

Sharma, S. and K. Guthrie (2006). "Nurse-led telephone consultation and outpatient local anaesthetic abortion: a pilot proiect." Journal of Family Planning and Reproductive Health Care **32**(1): 19-22.

Shearer, J. C., D. G. Walker and M. Vlassoff (2010). "Costs of post-abortion care in low- and middle-income countries." International Journal of Gynecology & Obstetrics **108**(2): 165-169.

Sheldon, S. and J. Fletcher (2017). "Vacuum aspiration for induced abortion could be safely and legally performed by nurses and midwives." Journal of Family Planning & Reproductive Health Care **43**(4): 260-264.

Singh, S. (2010). "Global consequences of unsafe abortion." Womens Health (Lond) **6**(6): 849-860.

Sjostrom, S., H. Kopp Kallner, E. Simeonova, A. Madestam and K. Gemzell-Danielsson (2016). "Medical Abortion Provided by Nurse-Midwives or Physicians in a High Resource Setting: A Cost-Effectiveness Analysis." PLoS One **11**(6): e0158645.

Sundari Ravindran, T. and S. Fonn (2011). "Are social franchises contributing to universal access to reproductive health services in low-income countries?" Reproductive Health Matters **19**(38): 85-101.

Tewari, S. K., I. R. Pirwany, T. A. Mahmood and R. J. Kelleher (1995). "MIFEPRISTONE IN MID-TRIMESTER TERMINATION OF PREGNANCY - VALUE FOR MONEY." British Journal of Family Planning **21**(1): 7-9.

Thapa, S., S. Neupana, I. Basnett, K. Ramnarayan and E. Read (2012). "Women having abortion in urban Nepal: 2005 and 2010 compared." Kathmandu Univ Med J (KUMJ) **10**(39): 8-13.

Thomas, J., S. Paranjothy and A. Templeton (2003). "An audit of the management of induced abortion in England and Wales." International Journal of Gynecology & Obstetrics **83**(3): 327-334.

Tunc, T. E. (2008). "Designs of devices: the vacuum aspirator and American abortion technology." Dynamis **28**: 353-376.

Tupper, C., S. Speed Andrews, C. Tupper and S. Speed Andrews (2007). "Setting up an outpatient service for early medical termination." Journal of Family Planning & Reproductive Health Care **33**(3): 199-202.

Upadhyay, U. D., A. F. Cartwright and N. E. Johns (2018). "Access to Medication Abortion Among California's Public University Students." Journal of Adolescent Health **63**(2): 249-252.

Upadhyay, U. D., N. E. Johns, R. Barron, A. F. Cartwright, C. Tapé, A. Mierjeski and A. J. McGregor (2018). "Abortion-related emergency department visits in the United States: An analysis of a national emergency department sample." BMC Medicine **16**(1): N.PAG-N.PAG.

Visaria, L., A. Barua and R. Mistry (2008). "Medical Abortion in India: Role of Chemists and Providers." Economic and Political Weekly **43**(36): 35-40.

Vlassoff, M., S. F. Musange, I. R. Kalisa, F. Ngabo, F. Sayinzoga, S. Singh and A. Bankole (2015). "The health system cost of post-abortion care in Rwanda." Health Policy & Planning **30**(2): 223-233.

Vlassoff, M., S. Singh, O. Tsuyoshi and T. Onda (2016). "The cost of post-abortion care in developing countries: a comparative analysis of four studies." Health Policy & Planning **31**(8): 1020-1030.

Vlassoff, M., D. Walker, J. Shearer, D. Newlands and S. Singh (2009). "Estimates of health care system costs of unsafe abortion in Africa and Latin America." International Perspectives on Sexual & Reproductive Health **35**(3): 114-121.

White, K., K. Adams and K. Hopkins (2019). "Counseling and referrals for women with unplanned pregnancies at publicly funded family planning organizations in Texas." Contraception **99**(1): 48-51.

Winikoff, B., D. Hassoun and H. Bracken (2011). "Introduction and provision of medical abortion: a tale of two countries in which technology is necessary but not sufficient." Contraception **83**(4): 322-329.

Wu, W.-J., S. Maru, K. Regmi and I. Basnett (2017). "Abortion Care in Nepal, 15 Years after Legalization
Gaps in Access, Equity, and Quality." Health and Human Rights **19**(1): 221-230.

Xia, W., S. She and T. H. Lam (2011). "Medical versus surgical abortion methods for pregnancy in China: a cost-minimization analysis." Gynecol Obstet Invest **72**(4): 257-263.
