# Supplementary material for: The mesoeconomics of abortion: A scoping review and analysis of the economic effects of abortion on health systems
Source: PLoS One. 2020 Nov 4;15(11):e0237227. doi: 10.1371/journal.pone.0237227 (PMC7641432; doi:10.1371/journal.pone.0237227)
Supplement: S2 Table — This table summarizes all studies reporting mesoeconomic impacts (n = 40). (PDF) [file pone.0237227.s002.pdf]

S2 Table. Summary table of included studies reporting mesoeconomic impacts (n=40)

Author, year [country]	Aim/objective(s)	Population	Study type	Summary of main findings
(Alouini, Uzan et al. 2002) [France]	To determine whether women undergoing repeat abortions are exposed to risk factors that might be amenable to preventative measures and the methods employed by carers in these cases	30 women who had undergone two abortions prior to or in 1997 at the Family Planning Centre of Hospital Jean Verdier in Bondy<br><br>The care team: two gynaecologists, two marital counsellors, two nurses and a senior nursing officer	Evaluation using a questionnaire for women and interviews with the care team	The medical manpower available to provide patients with psychological help is well below what is required to meet their needs.
(Baxerres, Boko et al. 2018) [Benin and Burkina Faso]	To document the means women use to obtain abortions in the capital cities of Benin and Burkina Faso<br><br>To learn whether or not use of misoprostol has become an alternative to other methods of abortion and the implications for future practice	Women in Cotonou (n=21) and in Ouagadougou (n=13), including 5 secondary school students	Qualitative in-depth interviews	In Benin, misoprostol can be obtained without prescription but is also available at informal drug markets, increasing exposure and use. International networks and information sharing between healthcare professional bodies (e.g., gynaecologists) and non-governmental organizations (NGOs) has increased awareness of misoprostol. Misoprostol was included on the essential medicines list in both Benin in November 2013 and Burkina Faso in December 2014.
(Berer 2000) [global]	To examine the changes in policy and health service provision required to make abortions safe	Varied due to a wide-ranging review of published and unpublished sources used, but includes women obtaining treatment for abortion complications, abortion providers, and those impacted by national policies	Literature review	Most authors agree that treating abortion complications in sub-Saharan Africa consumes a disproportionate amount of hospital resources.<br><br>In Bangladesh, up to 50% of hospital gynaecology beds are reportedly used for abortion complications. The cost and complexity of treatment is increased among women who wait to seek help until complications become severe. The use of untrained providers results in more visits by women and greater spending on care than the use of trained providers.<br><br>In Guyana, about 25% of the blood available at the main public hospital was used to treat abortion complications before the abortion law was liberalized.
(Bessett, Gorski et al. 2011) [United States]	To learn about women's experiences applying for subsidized insurance and to identify barriers to obtaining	English-speaking clients older than 18 who met the eligibility requirements for subsidized insurance	Systematic, qualitative interviews	MassHealth, a subsidized state health insurance program, does not enrol patients right away or cover abortion care provided by out-of-state providers. Although at least one woman was able to obtain and use Commonwealth Care for a medication abortion in a timely way, the approaching deadline prompted

Author, year [country]	Aim/objective(s)	Population	Study type	Summary of main findings
	insurance or its use for abortion services	programs in Massachusetts (n=39)		abortion funds to provide grants to at least two desperate women who were eligible. Both women were later deemed eligible for MassHealth, suggesting that the costs for abortion should have been covered by insurance. The delays caused by attempts to enrol in subsidized insurance had a disproportionate impact on women who sought medication abortion, forcing them to pay out of pocket or placing their preferred method for termination out of reach.
(Billings and Benson 2005) [Latin America and the Caribbean]	To review results from 10 major postabortion care (PAC) operations research projects conducted in public sector hospitals in seven Latin American countries, completed and published between 1991 and 2002	Varied by study but included patients, policymakers, administrators, and health care providers	Literature review of operations research studies	<u>Cost and resource use (time-motion) studies</u><br>Moving the uterine evacuation procedure from the operating theatre to an obstetrics-gynaecology ambulatory care area resulted in reducing costs and average length of patient stay.<br><br><u>Cost studies, including opportunity costs</u><br>Treating women with incomplete abortion can absorb more than 50% of facilities' obstetric and gynaecologic budgets. Switching to manual vacuum aspiration (MVA) while concurrently reorganizing services to an outpatient basis tends to substantially reduce average length of stay and treatment costs to the facility. While most studies reported marked decreases in average length of stay and costs when MVA was used to treat incomplete abortion in ambulatory settings, some showed differing results. A 1993 study in Ecuador showed that facilities that switched to MVA while continuing to treat postabortion patients with general anaesthesia in an operating room actually had similar average length of stay and costs than facilities that continued using sharp curettage (SC) under the same conditions. These data reinforce the conclusion that reorganizing services is critical for efficient services.<br><br>When SC was performed on an outpatient basis in Mexico, switching to outpatient MVA generated no additional savings over outpatient SC. Similarly in Peru, average length of stay between SC and MVA and cost per patient treated were comparable.<br><br>Reorganizing services can substantially reduce opportunity costs to the facilities. In some cases, administrators passed these savings on to the patients. Based on data in Peru, the director of the hospital cut patient fees in half for ambulatory patients (from approximately US$32 to $16). The hospital was recovering almost 98% of the full cost of providing PAC services at the 2000 follow-up compared with less than half (45%) prior to the intervention.
(Brown and Jewell 1996) [United	To estimate directly the responsiveness of abortion	Abortion providers in Texas, health facility data	Log-linear regressions	The location of facilities significantly impacts the cost of the abortion.

Author, year [country]	Aim/objective(s)	Population	Study type	Summary of main findings
States]	demand to county-level variations in travel-cost component of the full cost of abortion services			
(Coast, Norris et al. 2018) [global]	To present a new conceptual framework for studying trajectories to obtaining abortion-related care	Varied, as this is a global review paper, but includes health systems and those affected by national and international policies	Review paper	International institutions can shape the availability of abortion in other national and sub-national contexts, both ideologically and financially. Health system financing (e.g. free, subsidised, insurance, co-payments) affects how abortion-related care is sought and paid for. Legal settings increasingly recognize the scale and consequences of unsafe abortion, including the costs for health systems.
(Contreras, van Dijk et al. 2011) [Mexico]	To examine the experiences and opinions of health care professionals after the legalization of abortion in Mexico City in 2007	64 semi-structured interviews with obstetricians/gynaecologists, nurses, social workers, key decision makers at the Ministry of Health, and others	Qualitative study using semi-structured interviews	The number of conscientious objections was problematic for hospitals and health centres providing legal abortions; reports noted that these objections were not for moral or ethical reasons but rather to avoid extra workloads. Objectors also reported providing abortions in their private practices, suggesting financial incentives. Objecting professionals created hostile environments for care seekers, including making them wait longer for their services.
(Cook, de Kok et al. 2017) [Malawi]	To investigate factors contributing to the limited and declining use of MVA in Malawi	17 health workers of different cadres (doctors, nurses and clinical officers), genders and levels of experience and seniority who provided PAC in a central hospital and a district hospital	Small-scale qualitative study involving interviews supplemented by unstructured observations of care practices	Shortage of physical resources<br>MVA instruments are an increasing problem; there has not been a reliable supply to replace old and broken donated equipment. Unreliable equipment supplies, make MVA a more difficult option than curettage. In the absence of MVA equipment, providers would perform D&C rather than delay the procedure. As a result, health workers sometimes automatically resorted to D&C since they became accustomed to no MVA equipment.<br><br>Staff shortages (especially amongst those trained in PAC) and roles<br>Staff shortages led to staff feeling overworked and demotivated, potentially affecting quality of care. One or two nurses covered up to 70 patients. Unsafe abortions, thought to be increasing, contribute to a large proportion of admissions and may be compounding feelings of being overworked. When motivation and prioritization are low, staff will likely opt for the easier option, which may not be what is best for the patient. Staff shortages may also affect MVA use, since nurse shortages required nurses trained in MVA to engage with other tasks and junior interns received little on-the-job training in MVA. Furthermore, senior interns usually trained juniors only in D&C, in which they tended to be more confident. Participants said this is leading to MVA becoming a ‘forgotten skill.’

Author, year [country]	Aim/objective(s)	Population	Study type	Summary of main findings
				Demarcations between nurses and doctors in terms of role and status may have contributed to doctors discontinuing MVA, which became seen as a simple innovation of lower standing, designed for nurses.
(Crane and Dusenberry 2004) [global]	To examine the effect of the Global Gag Rule on family planning organizations in countries receiving American assistance	Family planning organizations within multiple countries	Literature review	Some organisations were forced to close clinics, terminate staff, and cut not only family planning programmes but also programmes for maternal health and well-baby care, sexual health education, youth outreach, and the prevention and treatment of sexually transmitted infections and HIV/AIDS. The Gag Rule also led to the termination of all American contraceptive supply shipments to leading family planning organisations in 29 countries.
(Cunningham, Lindo et al. 2017) [United States]	To document the effects of abortion-clinic closures on clinic access, abortions, and births using variation generated by a law that shuttered nearly half of Texas' clinics	Women of reproductive age and children	Regression analysis	In the immediate aftermath of House Bill 2 (HB2), the average clinic service population rose from 150,000 to 290,000 in Texas. This occurred for two reasons: (1) as clinics closed in small cities, women had to travel to clinics that remained in larger cities, shrinking the number and expanding the sizes of service regions; and (2) as clinics closed in large cities, there were fewer providers of abortion services. There is substantial variation in how access changed across Texas. The average service population did not change in eastern Texas, where only one clinic closed in the fourth quarter of 2013 (though several closed the following year). In the Dallas-Fort Worth region, where distances had not changed, the average service population increased by 250,000. Clinic closures continued through 2014, and the average service population continued to rise. In Dallas-Fort Worth, an additional clinic closure in June 2015 increased the average service population from 380,000 to 480,000.
(Díaz-Olavarrieta, Cravioto et al. 2012) [Mexico]	To identify the perceptions and opinions of people who provide abortion services in Mexico City, three years after implementation of elective abortion legal reforms	19 health workers assigned to the legal abortion programs at a clinic and a hospital in Mexico's Federal District	In-depth interviews	Payment exemptions for women that have any form social security plan or are vested in other institutions are considered by clinic personnel to negatively impact the quality of the services because these exemptions generate work overload and material and human resources shortages.
(Doran and Nancarrow 2015) [United States, Canada, Australia, New Zealand, France, Norway, Sweden, Norway, and the United Kingdom]	To identify the factors that facilitate and hinder access to abortion services for women in developed countries in relation to first-trimester abortions, from the perspective of both the woman and the service provider	As a systematic review, included studies looked at multiple populations including women seeking abortions and abortion providers	Systematic review	A Canadian study reported that waiting times for an abortion are significantly shorter in private clinics than for government-funded services.
(Duggal 2004)	To examine the political economy	This national-level analysis	Descriptive	While there is no dearth of medical providers who can be certified to perform

Author, year [country]	Aim/objective(s)	Population	Study type	Summary of main findings
[India]	of abortion care in India by reviewing cost and expenditure patterns for abortion care	includes health providers	analysis of the political economy	abortions, unregistered and illegal abortions continue to take place in overwhelmingly large numbers. Legalisation of abortion in 1971 potentially provided the medical profession with a monopoly over abortion and a means to medicalise it. Legal abortion services began to expand but did not significantly threaten traditional abortion providers. On the contrary, abortion was seen as a growing business and many medical practitioners, unqualified and untrained in abortion, entered the fray. Since regulation of medical practice remained weak, this put a damper on the expansion of legal services.<br><br>Abortion services have remained predominantly in the private sector. The state has played a subtler role by providing subsidies to select private abortion providers if they make abortion provision dependent on acceptance of sterilisation or an intrauterine device. An unmet demand for public abortion services and the lack of any effective regulatory mechanisms further opened the floodgates for all sorts of private providers, unqualified persons, non-allopathic doctors, and paramedics.<br><br>Despite early legalisation of abortion, the problem of illegal providers and unsafe abortion looms large. This translates into a political economy of abortion which is controlled by providers, with those who are unqualified and unregistered exploiting the vulnerability of women seeking abortion and contributing to widespread post-abortion problems and mortality. Given that a large number of providers are unqualified to do abortions, the cost of unsafe abortions must also be factored in. Post-abortion costs due to botched abortions and complications could be high. The insistence on curettage even for very early abortion, and so- called check curettage after vacuum aspiration, is widespread amongst both certified and non-certified providers, adding to the cost as well the risk of post-abortion infections and other problems.
(Erim, Resch et al. 2012) [Nigeria]	To synthesize the best available data, adapt a model of pregnancy and pregnancy-related morbidity and mortality to the Nigerian context, and conduct national and regional analyses that quantify the payoffs from investing in safe pregnancy and childbirth to provide qualitative insight into the most efficient	National level estimates	Synthesize the best available data, adapt a model of pregnancy and pregnancy-related morbidity and mortality to the Nigerian context, and	Strategies that only improved family planning and safe abortion had very low cost-effectiveness ratios (i.e., very attractive), but reduced mortality by 20.5%. A strategic approach that involves simultaneous improvement in intrapartum care, family planning, and safe abortion was the most efficient and associated with incremental cost-effectiveness ratios between the two aforementioned strategies.<br><br>Strategies that scale up maternal health services by systematically making stepwise improvements in family planning, safe abortion, and intrapartum care will be more effective and efficient in the long-run than solely focusing on any

Author, year [country]	Aim/objective(s)	Population	Study type	Summary of main findings
	strategies to meet Millennium Development Goal 5		conduct national and regional analyses that quantify the payoffs from investing in safe pregnancy and childbirth	one of these alone. A strategy that involved phasic and concurrent improvements in the availability and standard of emergency obstetric care facilities, referral systems, access to skilled birth attendants, facility deliveries, availability and use modern contraceptives and access to safe abortion services could prevent three to four out of five maternal deaths. This strategy had cost-effectiveness ratios that were a fraction of Nigeria's per capita gross domestic product.
(Foster, LaRoche et al. 2017) [Canada]	To document women's experiences obtaining abortion care in New Brunswick (NB) before and after the Regulation 84-20 amendment; identify the economic and personal costs associated with obtaining abortion care; and examine the ways in which geography, age and language-minority status condition access to care.	New Brunswick residents who received abortion services (n=36)	Qualitative: semi-structured interviews	Although the 2015 changes to Regulation 84-20 represent an important step in aligning New Brunswick with the rest of Canada, the amendment does little to mitigate the challenges imposed by the province's refusal to fund clinic-based abortion care within or outside of the province. Even if the elimination of the two-physician requirement were to be fully implemented, this would have only marginal impact on women's ability to access affordable and timely abortion care.
("Fourteenth Amendment" 2016) [United States]	1. To review the Whole Woman's Health v. Hellerstedt case, in which the Supreme Court held two health-related abortion restrictions unconstitutional under the undue burden standard<br><br>2. To discuss how the Court resolved much of the doctrinal uncertainty that plagued the undue burden standard, but it allowed for continued judicial discretion where abortion restrictions fit less comfortably into a cost-benefit framework	Those impacted by state-level and national policy/law, including abortion providers and women seeking abortions	Law review	<u>Inability to meet demand for abortion services</u><br>The court noted that few remaining clinics would be unable to meet demand for abortion services, due to the admitting-privileges requirement that closed almost half of the state's abortion facilities and the surgical-centre requirement that would lead to more closures. Over 75% of Texan clinics closed as a result of these two requirements. The Court concluded that the closures created a substantial obstacle by severely limiting abortion access, that the quality of care would likely decline due to fewer clinics managing the same demand, and that the state had the burden to prove that remaining clinics could meet this demand.<br><br><u>Clarity added to undue burden standard</u><br>The Court added clarity to the undue burden standard by translating the undue burden inquiry into a cost-benefit analysis framework that will likely be applied with greater consistency. However, the court's decision may provide less guidance where courts are asked to weigh burdens and benefits that fit less neatly into a cost-benefit framework. Since the undue burden standard remains a standard, its application depends upon the personal beliefs of judges and Justices. Thus, it remains to be seen precisely how much order Whole

Author, year [country]	Aim/objective(s)	Population	Study type	Summary of main findings
				Woman's Health will bring to the Court's abortion jurisprudence.
(Haddad, Yanow et al. 2009) [United States]	To explore provider responses to the Partial-Birth Abortion Ban Act of 2003 in one state in order to identify important indicators for a national study	Facilities that provided second-trimester abortions in Massachusetts	Key informant surveys	In reference to the 2007 Supreme Court decision to uphold the Partial-Birth Abortion Ban Act, three hospital facilities increased their charges from second-trimester abortion services, attributing these increases to the cost of providing care since the ban. State regulations specify that abortions beyond 18 weeks and 6 days will only be reimbursed if performed at a hospital; therefore, people seeking abortions at 19 weeks or later must rely on hospitals to secure Medicaid. Medicaid reimbursement rates did not increase with the increased procedure costs after the ban, thus making abortions more expensive to provide.
(Hendrickson, Fetters et al. 2016) [Zambia]	To examine sales practices, knowledge, and behaviour of pharmacy workers regarding medical abortion in 2009 and 2011 in Zambia, where hostile and stigmatizing attitudes still result in high rates of unsafe abortion	Pharmacy workers at government-certified pharmacies in the intervention areas (76 pharmacies in November 2009 and 80 in November 2011)	Descriptive cross-sectional design	Most pharmacy workers mentioned valid medical abortion products to the mystery clients; however, a far smaller number offered to sell mystery clients these medications. Among the pharmacy workers who offered to sell mystery clients a drug or offered information, 51% offered to sell the client a known and approved medical abortion drug in 2009, compared with 72% in 2011 ($P = 0.0380$). Pharmacy workers offered either Cytotec or an imported Chinese pill. None offered mifepristone although it was available in the study areas. Among those offering known medical abortion drugs, an increase was observed in 2011 in the number who provided important information on medical abortion; however, the provision of specific information was still low overall. None of the pharmacy workers who offered to sell misoprostol in 2009 mentioned the correct number of tablets for initial dosage the patient would need to induce a medical abortion; in 2011, 21% of the pharmacy workers included in the study did provide the correct dosage information ($P = 0.0185$). In 2011, all of the pharmacy workers who offered to sell medication to the mystery clients also provided one or more pieces of correct information about medical abortions; this is in comparison with only 78% providers in 2009 that provided similar information ($P < 0.001$).
(Hu and Schlosser 2010) [India]	To study the impact of prenatal sex selection on the wellbeing of girls by analysing changes in children's nutritional status and mortality during the years since the diffusion of sex-selective abortion in India	Households and children (the last two children born within 3 years prior to each survey round)	Econometric analysis of national survey data	Estimates of the main male-female ratio (MFR) effect suggest that regions with increasing MFR experienced improvement in some family characteristics, in particular, an increase in the level of parental education and mother's age at first birth and a decline in the likelihood of living in a rural area. On the other hand, there is no association between MFR and maternal age or the household wealth index.
(Johnston, Oliveras et al. 2010) [Bangladesh]	To estimate comparative costs to the Bangladesh health system of providing menstrual regulation	Government health facilities that provide menstrual regulation or care for	Cost estimates	On a per-case basis, the incremental costs of providing menstrual regulation and related care following recommended practices (i.e., use of MVA for uterine evacuation, providing services in outpatient facilities, having midlevel providers

Author, year [country]	Aim/objective(s)	Population	Study type	Summary of main findings
	and care for abortion complications	abortion complications<br><br>Facilities were purposively and systematically selected by location (urban or rural), facility level (primary, secondary or tertiary) and division or district performance (high, median, or low rate of procedures performed)		offer uterine evacuation and other care, and providing contraceptive counselling and services) are much lower than those associated with providing care for abortion complications using conventional practices. On a per-case basis, provision of menstrual regulation care cost 8-13% of the cost of treatment for severe abortion complications, depending on the level of care at which menstrual regulation and PAC were offered. By providing menstrual regulation care as a basic service, the Bangladesh government is providing women with much-needed care and preventing unnecessary and expensive complications.<br><br>The menstrual regulation program has played an important role in reducing abortion-related morbidity and mortality, and thereby has also played an important role in reducing health system costs associated with treating abortion-related complications. If all women experiencing abortion-related complications were able to access the care they needed, the total annual health system costs of providing care for abortion-related complications would be much higher. The Bangladesh health system could better meet the reproductive health needs of women by making menstrual regulation more accessible, further reducing recourse to unsafe abortion.
(Johnston, Akhter et al. 2012) [Bangladesh]	To assess incremental health system costs of service delivery for abortion-related complications in the Bangladesh public health system and confirmed that providing PAC with vacuum aspiration is less expensive than using dilation and curettage	Public-sector health facilities in Bangladesh (n=17)	Qualitative: purposive sampling and informational interviews	Despite the presence of a decentralized menstrual regulation program, designed in part to reduce complications of unsafe abortion, abortion complications remain a frequent event. On average, each tertiary facility received more than four patients with abortion-related complications every day; this is consistent with previously generated estimates that suggest the public health system treats roughly 70,000 such patients annually.
(Jones and Weitz 2009) [United States]	To addresses a set of laws—laws that directly target abortion providers, make the provision of abortion more difficult and costly, and provide strong incentives for physicians not to offer abortion services—which have a significant capacity to reduce access to and quality of abortion care in the United States	Those impacted by the policies, including health facilities providing abortions, facility staff, and women seeking abortions	Legal review	The type of regulation unnecessarily requires abortions to be performed in ambulatory surgery centers (ASC) set up for more sophisticated and intrusive surgical procedures. These costly requirements may force many providers to stop offering services or to raise their prices to levels prohibitive for some women seeking care. The regulations require the practice of abortion care to change without regard to evidence or clinical judgment and reduce access to quality second-trimester abortion care.<br><br>ASC regulations are generally quite extensive and are often extremely costly for abortion providers to comply with. These costs are particularly onerous in

Author, year [country]	Aim/objective(s)	Population	Study type	Summary of main findings
				states that apply the physical plant requirements of their ASC regulations to existing abortion facilities, rather than grandfathering those facilities for purposes of construction standards until such time as the facilities move or undertake substantial renovations. For example, the physical renovations alone at one facility would cost $750,000, according to the administrator of a Texas abortion clinic. The costs and burdens stemming from the imposition of ASC requirements have hindered or prevented physicians in some states from providing abortions.
(Jones 2015) [Ghana]	To examine Ghanaian women's response to a reduction in the availability of modern contraceptives in terms of contraceptive access and use, resulting pregnancies, use of induced abortion, and resulting births. The exogenous change in availability results from a United States policy, driven entirely by domestic politics, that cut funding to NGOs providing reproductive health services in poor countries	Individual women aged 17-25 years in a nationally representative, cross-sectional sample from the Ghana Demographic and Health Surveys (n=24,500)	Regression analysis	Though NGOs providing reproductive health services closed clinics in both rural and urban areas, they reported that the primary change in service provision resulting from the funding cuts was a reduction in contraceptive supplies and outreach in rural areas. On the basis of clinic availability data, stocks of modern birth control methods dropped by about 10% nationwide, driven by reductions in the private sector.
(Kacanek, Dennis et al. 2010) [United States]	To investigate three questions:<br>1) What are providers' experiences with Medicaid reimbursement for abortion in cases of rape, incest and life endangerment?<br>2) What is the process for applying Medicaid reimbursement?<br>3) What factors facilitate or hinder reimbursement?	25 abortion providers in six states with restrictive abortion laws	Qualitative interviews	<u>Financial issues</u><br>Respondents reported varying reimbursement rates. Some stated that rates were so low, they would not be able to stay in business if they continued to bill Medicaid. Some providers received a flat reimbursement regardless of the gestational age at abortion, even though the cost of abortion increases as the pregnancy progresses. One respondent reported that Medicaid reimbursed $212 for an abortion that cost $420. All but two respondents reported relying on abortion funds. If Medicaid reimbursed all qualifying cases, abortion funds would be able to cover more women who are ineligible for Medicaid.<br><br><u>Giving up on Medicaid</u><br>Some respondents no longer contracted with Medicaid, or worked with Medicaid as little as possible, because of the numerous administrative and systematic barriers they experienced. Because of low reimbursement rates and the expense of staff time to pursue Medicaid dollars, many providers avoid working with the system by offering discounts, providing loans, or absorbing

Author, year [country]	Aim/objective(s)	Population	Study type	Summary of main findings
				the costs of abortions themselves. Some respondents said that they absorb between $1,000 and $60,000 annually in free or reduced-cost services.
(Mercier, Buchbinder et al. 2016) [United States]	To describe recent research with abortion providers in North Carolina to illustrate how providers adapt to new regulations, and how compliance with regulation leads to increased workload and increased financial and emotional burdens on providers	31 healthcare professionals in North Carolina who were involved in multiple aspects of abortion provision at 11 distinct clinical practices	Interviews	Most providers made changes to meet the law's requirements and also to minimize the burden of the law on patients. For example, they chose to implement telephone counselling rather than require two in-person clinic visits. Implementation of telephone counselling, however, required significant adaptations. Several high-volume providers hired additional nurses and developed a call-centre infrastructure with dedicated staff for telephone counselling. Lower-volume providers responded to the new requirements by changes in scheduling and work tasks. Providers who worked in small or solo-practice clinics frequently extended the hours of existing staff to meet demands. In general, we observed a trade-off between cost and time burdens, depending on the practice type and structure. Costs were greatly increased by the requirement that a licensed medical professional perform the state-mandated counselling. The clinic administrator noted at one large abortion clinic in an urban setting described the law as having "a huge financial impact ...you know, nurses are expensive." In contrast, for lower-volume clinics, solo practitioners, and hospital-based clinics, costs did not increase, as no additional staff was hired. However, the providers, typically physicians, described how they and their colleagues worked more uncompensated hours to meet the law's requirements. In general, providers absorbed both the financial and time burden of these changes. No providers reported increasing their prices to compensate, and several specifically stated that they made an explicit decision not to pass the cost onto patients. Larger practices with greater resources performed dedicated fundraising activities to support the increased costs.
(Mutua, Manderson et al. 2018) [Kenya]	To illustrate how the quality of PAC in healthcare facilities is impacted by law and government policy	21 patients and 16 providers (eight were nurses and eight were clinical officers)	Cross-sectional design with in-depth interviews	Increased access to abortifacients<br>In 2016, misoprostol and mifepristone were registered and included as essential drugs for the management of obstetric and gynaecological indications, resulting in increased access. As a result, the use of crude means of abortion termination decreased. However, some service providers found challenges with the unregulated access of these drugs, often without proper controls on drug prescription and usage.<br><br>Evidence of capacity gaps<br>Emergency care was significantly impeded by the unavailability of the right providers for certain procedures, especially doctors, and during the night, weekends, or public holidays. These capacity gaps were also evident in the

Author, year [country]	Aim/objective(s)	Population	Study type	Summary of main findings
				number of patients who required multiple evacuations, often leading to longer hospital stays and increased the cost of care to both the patients and the healthcare system.
(Osur, Baird et al. 2013) [Kenya, Uganda]	To evaluate implementation of misoprostol for PAC (MPAC) in two African countries	Public and private health facilities in Rift Valley Province, Kenya, and Kampala Province, Uganda (n=25)<br><br>MPAC providers, health facility managers, Ministry of Health officials, and NGO staff involved in program implementation (n=45)	Pre/post intervention study using a comprehensive site assessment tool (pre-implementation) and an independent evaluation including in-depth qualitative interviews (post-implementation)	MPAC providers believed that use of misoprostol offered women greater privacy, reduced cost, and the option of a non-invasive treatment for their incomplete abortion.
(Ramos and Rios 2015) [Peru]	To explore debates of whether a woman with an incomplete abortion should be treated with surgical procedures or medical procedures	Debates on the topic of MVA versus MA in Peru	Literature review	Treating incomplete abortion has substantial costs for the health system.
(Roberts, Gould et al. 2014) [United States]	To describe payment for abortion care before new restrictions among a sample of women receiving first and second trimester abortions	English- and Spanish-speaking women aged 15 and older, with no known fetal anomalies or demise, presenting for abortion care at one of 30 facilities throughout the United States between January 2008 and December 2010 and meeting specific gestational age criteria	Interviews and regression analysis	There are significant gaps in public and private insurance coverage for abortion.
(Sagala 2005) [sub-Saharan Africa]	To examine the effect of the Global Gag Rule on family planning organizations in countries receiving American assistance	Family planning organizations within multiple countries	Literature review	In Zambia, the Planned Parenthood Association of Zambia (PPAZ) lost 24% of its core grant for refusing to sign a pledge to enforce the Global Gag Rule. PPAZ further lost $137,092 in contraceptive supplies. The loss of funds disrupted family planning services and hindered the design and production of training materials. In September 2003, the Planned Parenthood Association of Ghana

Author, year [country]	Aim/objective(s)	Population	Study type	Summary of main findings
				(PPAG) refused to sign a pledge to enforce the Global Gag Rule and lost more than $200,000. This led to cutbacks in PPAG's family planning programs.<br><br>The loss of funding from USAID has shattered rural outreach programs by reducing nursing staff by more than 40%. In Ethiopia, the Global Gag Rule requirements forced the Family Guidance Association of Ethiopia (FGAE) to sever its ties with Pathfinder International, a sub-grantee of USAID. USAID responded by defunding FGAE. With the defunding, FGAE no longer services some 300,000 clients, who desperately need reproductive health care services that the Ethiopian government cannot provide. In addition, the Family Planning Association of Ethiopia lost $56,000; money that would have been used to procure and distribute much needed contraceptives.<br><br>With the implementation of the Global Gag Rule directives, USAID/Kenya-NGO partnerships have broken down and several NGO run reproductive health clinics have been gutted. The Global Gag Rule curtailed family planning and maternal and child health care services and weakened the collective Kenyan NGO response to HIV/AIDS. The Global Gag Rule has also forced the Family Planning Association of Kenya (FPAK) and Marie Stopes Clinic to close down their major urban clinics and service centres. FPAK lost more than $580,000 and consequently closed three clinics that served over 56,000 poor and underserved clients. Similar results have been recorded in Senegal and Zimbabwe.
(Schaff and Schaff 2010) [United States]	To review evidence relating to mifepristone ten years after its legalization in the United States	Evidence on mifepristone across the United States	Review	Medical liability insurance for medical abortion has reportedly been denied or is cost-prohibitive for non-specialists; as a result, primary care physicians are unable to afford the costs of insurance to practice.
(Schiavon, Collado et al. 2010) [Mexico]	To know which factors (e.g., fear of staff attitudes, ignorance about the law, lack of information on where to access services, fear of breaches in confidentiality, burdensome requirements) were driving women in Mexico City to continue seeking private abortion services despite the availability of low-cost, safe, legal abortion services in the public sector	135 physicians in the private sector who confirmed they were direct abortion providers	Descriptive	Nearly half of surveyed abortion providers reported that the number of women seeking abortion services at their facilities had increased since legalization, while 10% reported that the demand had decreased. Nine in ten respondents said they had started offering abortion services only after the legalization of abortion, suggesting that legalization may have led to an increase in abortion providers. Alternatively, some of these providers may simply have been reluctant to admit they had been providing abortions before legalization.
(Sheldon and	To provide a detailed	Medically-trained abortion	Reassessment of	Allowing nurses to provide vacuum aspiration for induced abortion potentially

Author, year [country]	Aim/objective(s)	Population	Study type	Summary of main findings
Fletcher 2017) [United Kingdom]	reassessment of the relevant law (on doctors performing MVA procedures) and the clinical evidence that supports this assumption	providers (doctors, nurses, midwives)	relevant law and clinical evidence that supports the law's assumption	offers a more sustainable and economically efficient basis for the long-term development of excellent care. It would free up doctors to focus on those aspects of service provision where their specific expertise is needed. It might also improve the job satisfaction of nursing and midwifery staff, potentially impacting positively on sickness absence and staff retention rates.
(Singh 2010) [global]	To review the scientific evidence on the consequences of unsafe abortion, highlight gaps in the evidence base, suggest areas where future research efforts are needed, and speculate on the future situation regarding consequences and evidence over the next 5–10 years	Women who obtained unsafe abortions	Literature review	A recent study in Nigeria (1) estimated the cost of providing the necessary contraceptive services and supplies to prevent the unintended pregnancies that resulted in unsafe abortions and (2) compared this cost with the cost of providing PAC. It found that the cost-to-benefit ratio is $4:$1. Other types of economic costs have not been studied because of the difficulty in collecting data to document these costs.
(Sjostrom, Kopp Kallner et al. 2016) [Sweden]	To calculate the cost-effectiveness of early medical abortion performed by nurse-midwifes in comparison to physicians in a high resource setting where ultrasound dating is part of the protocol	Healthy women seeking treatment for abortion at an outpatient clinic of a university hospital	Cost effectiveness analysis	In a high-resource setting where ultrasound dating is part of the protocol, the randomized-controlled equivalence trial showed that provision of medical abortion by nurse-midwifes was superior to provision by physicians, with a risk difference for effectiveness, complete abortion without surgical intervention, of 1.6% (95% CI; 0.2–3.6%, p = 0.027). This means that for every 100 patients (procedures), the intervention treatment resulted in 1.6 fewer follow-up surgical abortions than the standard treatment. The authors also examined the incremental cost effectiveness (ICER) of different measures of costs. For the direct cost per woman treated, the difference in costs per case ranged from € 45–58.3, and the difference in efficacy per case was 0.016. The ICER was -831.2. For the direct costs including waiting time for the consecutive patient, the difference in costs per case ranged from € 41–58.3, and the difference in efficacy per case was 0.016. The ICER was -1081.2. Lastly, for the total direct and indirect costs, the difference in costs per case ranged from € 78–106.3, and the difference in efficacy per case was 0.016. The ICER was -1768.8.
(Vlassoff, Walker et al. 2009) [global]	To estimate the health system costs of PAC in Africa and Latin America	Top-down approach: PAC patients<br><br>Bottom-up approach: women hospitalized for serious medical complications of induced abortion	Cost estimations	The health care system cost of treating serious abortion complications is only one component of the total economic impact on society of unsafe abortion. The costs of treating long-term health consequences, such as chronic pelvic infections and infertility, have hardly been studied. The indirect economic costs of unsafe abortion are also essentially unmeasured. The health system costs of PAC in Africa and Latin America ranged from $159 million to $476 million per year, depending on the estimation method used. The average estimates from the two approaches largely coincide: $280 million using the top-down approach, and $274 million using the bottom-up approach (averaging actual

Author, year [country]	Aim/objective(s)	Population	Study type	Summary of main findings
				and standard practice estimates). These considerable sums impose an added burden on the already overstretched health resources of developing countries.
(Wu, Maru et al. 2017) [Nepal]	To review abortion care in Nepal 15 years after it was legalized	Abortion providers, women seeking abortions, those impacted by national policies	Review of current status	Implementing first-trimester medical abortion services has further expanded abortion access, since medical abortion can be more easily provided in rural areas. Medical abortions now constitute over 50% of all abortions in Nepal. Facilities providing first-trimester medical abortions do not need to have surgical abortion capacity. Preventing medication stock-outs at remote health care facilities is critical. In some areas, the supply chain for medical abortions has been poorly managed and there are reports of women being denied legal abortions due to a lack of abortion medications. This has been further complicated by the black market for medical abortion medications of questionable quality, especially along the Indian border. Private pharmacies have emerged as a prevalent dispenser of medical abortion medications, although most pharmacists are not government-approved to do so and have not had adequate training on medical abortion counselling.<br><br>United States foreign policy continues to influence the implementation of safe abortion services in Nepal. As a consequence of the Helms Amendment, many government and non-profit clinics receiving USAID funding cannot provide abortions. USAID selectively supports post-abortion care and artificially separates it from comprehensive abortion care. While the same manual vacuum aspirator can be used to perform both abortions and post-abortion care, many USAID-supported clinics will perform only the latter while turning away women seeking services for the former. These funding restrictions marginalize abortion services from the existing health care system and create clinics that provide less efficient care. In the early 2000s, when the Global Gag Rule was active, several Nepali organizations rejected the terms of the rule and, in turn, suffered significant funding losses that resulted in program cutbacks and layoffs.
(Zamberlin, Romero et al. 2012) [Latin America]	To summarize the findings of a literature review on women's experiences with medical abortion in Latin American countries where voluntary abortion is illegal	Studies on women's experiences with medical abortion in Latin American countries where voluntary abortion is illegal	Literature review	Difficulty in obtaining misoprostol was related to different regulatory environments (e.g., local, governmental control of pharmacy sales). Stricter controls restrict access and push pregnant people to access misoprostol through unofficial markets at higher prices. Where misoprostol is sold under prescription, women used strategies to obtain a prescription or buy misoprostol without one, including paying for a prescription, claiming the drug is not for gynaecological purposes (e.g., obtaining it from a non-gynaecological specialist), or asking an older man or woman to buy the drug on their behalf.

References

"Fourteenth Amendment" (2016). "Fourteenth Amendment - Due Process Clause - Undue Burden - Whole Woman's Health v. Hellerstedt." Harvard Law Review **130**(1): 397.

Alouini, S., M. Uzan, J. P. Méningaud and C. Hervé (2002). "Knowledge about contraception in women undergoing repeat voluntary abortions, and means of prevention." European Journal of Obstetrics & Gynecology and Reproductive Biology **104**(1): 43-48.

Baxerres, C., I. Boko, A. Konkobo, F. Ouattara and A. Guillaume (2018). "Abortion in two francophone African countries: a study of whether women have begun to use misoprostol in Benin and Burkina Faso." Contraception **97**(2): 130-136.

Berer, M. (2000). "Making abortions safe: a matter of good public health policy and practice." Bull World Health Organ **78**(5): 580-592.

Bessett, D., K. Gorski, D. Jinadasa, M. Ostrow and M. J. Peterson (2011). "Out of Time and Out of Pocket: Experiences of Women Seeking State-Subsidized Insurance for Abortion Care in Massachusetts." Women's Health Issues **21**(3, Supplement): S21-S25.

Billings, D. L. and J. Benson (2005). "Postabortion care in Latin America: policy and service recommendations from a decade of operations research." Health Policy Plan **20**(3): 158-166.

Brown, R. W. and R. T. Jewell (1996). "The Impact of Provider Availability on Abortion Demand." Contemporary Economic Policy **14**(2): 95-106.

Coast, E., A. H. Norris, A. M. Moore and E. Freeman (2018). "Trajectories of women's abortion-related care: A conceptual framework." Social Science & Medicine **200**: 199-210.

Contreras, X., M. G. van Dijk, T. Sanchez and P. S. Smith (2011). "Experiences and Opinions of Health-Care Professionals Regarding Legal Abortion in Mexico City: A Qualitative Study." Studies in Family Planning **42**(3): 183-190.

Cook, S., B. de Kok and M. L. Odland (2017). "'It's a very complicated issue here': understanding the limited and declining use of manual vacuum aspiration for postabortion care in Malawi: a qualitative study." Health Policy & Planning **32**(3): 305-313.

Crane, B. B. and J. Dusenberry (2004). "Power and Politics in International Funding for Reproductive Health: the US Global Gag Rule." Reproductive Health Matters **12**(24): 128-137.

Cunningham, S., J. M. Lindo, C. Myers and A. Schlosser (2017). How Far Is Too Far? New Evidence on Abortion Clinic Closures, Access, and Abortions, National Bureau of Economic Research, Inc, NBER Working Papers: 23366.

Díaz-Olavarrieta, C., V. M. Cravioto, A. Villalobos, N. Deeb-Sossa, L. García and S. G. García (2012). "Mexico City's Legal Abortion Program: health workers' experiences." Revista Panamericana de Salud Publica **32**(6): 399-404.

Doran, F. and S. Nancarrow (2015). "Barriers and facilitators of access to first-trimester abortion services for women in the developed world: a systematic review." Journal of Family Planning & Reproductive Health Care **41**(3): 170-180.

Duggal, R. (2004). "The political economy of abortion in India: cost and expenditure patterns." Reproductive Health Matters **12**: 130-137.

Erim, D. O., S. C. Resch and S. J. Goldie (2012). "Assessing health and economic outcomes of interventions to reduce pregnancy-related mortality in Nigeria." BMC Public Health **12**: 786.

Foster, A. M., K. J. LaRoche, J. El-Haddad, L. DeGroot and I. M. El-Mowafi (2017). ""If I ever did have a daughter, I wouldn't raise her in New Brunswick:" exploring women's experiences obtaining abortion care before and after policy reform." Contraception **95**(5): 477-484.

Haddad, L., S. Yanow, L. Delli-Bovi, K. Cosby and T. A. Weitz (2009). "Changes in abortion provider practices in response to the Partial-Birth Abortion Ban Act of 2003." Contraception **79**(5): 379-384.

Hendrickson, C., T. Fetters, S. Mupeta, B. Vwallika, P. Djemo and K. Raisanen (2016). "Client–pharmacy worker interactions regarding medical abortion in Zambia in 2009 and 2011." International Journal of Gynecology & Obstetrics **132**(2): 214-218.

Hu, L. and A. Schlosser (2010). Does sex-selective abortion improve girls' well-being? evidence from India, Federal Reserve Bank of Chicago, Working Paper Series: WP-2010-11.

Johnston, H. B., S. Akhter and E. Oliveras (2012). "Quality and efficiency of care for complications of unsafe abortion: a case study from Bangladesh." International Journal of Gynecology & Obstetrics **118**: S141-147.

Johnston, H. B., E. Oliveras, S. Akhter and D. G. Walker (2010). "Health System Costs of Menstrual Regulation and Care For Abortion Complications in Bangladesh." International Perspectives on Sexual & Reproductive Health **36**(4): 197-201.

Jones, B. S. and T. A. Weitz (2009). "Legal barriers to second-trimester abortion provision and public health consequences." American Journal of Public Health **99**(4): 623-630.

Jones, K. M. (2015). "Contraceptive Supply and Fertility Outcomes: Evidence from Ghana." Economic Development and Cultural Change **64**(1): 31-69.

Kacanek, D., A. Dennis, K. Miller and K. Blanchard (2010). "Medicaid funding for abortion: providers' experiences with cases involving rape, incest and life endangerment." Perspectives on Sexual & Reproductive Health **42**(2): 79-86.

Mercier, R. J., M. Buchbinder and A. Bryant (2016). "TRAP laws and the invisible labor of US abortion providers." Critical Public Health **26**(1): 77-87.

Mutua, M. M., L. Manderson, E. Musenge and T. N. O. Achia (2018). "Policy, law and post-abortion care services in Kenya." Plos One **13**(9): 18.

Osur, J., T. L. Baird, B. A. Levandowski, E. Jackson and D. Murokora (2013). "Implementation of misoprostol for postabortion care in Kenya and Uganda: a qualitative evaluation." Glob Health Action **6**: 1-11.

Ramos, M. G. and E. G. Rios (2015). "Controversies in the treatment of incomplete abortion, MVA versus medical treatment with misoprostol." Revista Peruana De Ginecologia Y Obstetricia **61**(1): 57-64.

Roberts, S. C. M., H. Gould, K. Kimport, T. A. Weitz and D. G. Foster (2014). "Out-of-Pocket Costs and Insurance Coverage for Abortion in the United States." Women's Health Issues **24**(2): e211-218.

Sagala, J. K. (2005). "Bush's global gag rule and Africa: impact on reproductive health." International Journal of Global Health & Health Disparities **4**(1): 13-29.

Schaff, E. A. and E. A. Schaff (2010). "Mifepristone: ten years later." Contraception **81**(1): 1-7.

Schiavon, R., M. E. Collado, E. Troncoso, J. E. Soto Sánchez, G. O. Zorrilla and T. Palermo (2010). "Characteristics of private abortion services in Mexico City after legalization." Reproductive Health Matters **18**(36): 127-135.

Sheldon, S. and J. Fletcher (2017). "Vacuum aspiration for induced abortion could be safely and legally performed by nurses and midwives." Journal of Family Planning & Reproductive Health Care **43**(4): 260-264.

Singh, S. (2010). "Global consequences of unsafe abortion." Womens Health (Lond) **6**(6): 849-860.

Sjostrom, S., H. Kopp Kallner, E. Simeonova, A. Madestam and K. Gemzell-Danielsson (2016). "Medical Abortion Provided by Nurse-Midwives or Physicians in a High Resource Setting: A Cost-Effectiveness Analysis." PLoS One **11**(6): e0158645.

Vlassoff, M., D. Walker, J. Shearer, D. Newlands and S. Singh (2009). "Estimates of health care system costs of unsafe abortion in Africa and Latin America." International Perspectives on Sexual & Reproductive Health **35**(3): 114-121.

Wu, W.-J., S. Maru, K. Regmi and I. Basnett (2017). "Abortion Care in Nepal, 15 Years after Legalization
Gaps in Access, Equity, and Quality." Health and Human Rights **19**(1): 221-230.

Zamberlin, N., M. Romero and S. Ramos (2012). "Latin American women's experiences with medical abortion in settings where abortion is legally restricted." Reprod Health **9**(1): 34.
