# Supplementary material for: The mesoeconomics of abortion: A scoping review and analysis of the economic effects of abortion on health systems
Source: PLoS One. 2020 Nov 4;15(11):e0237227. doi: 10.1371/journal.pone.0237227 (PMC7641432; doi:10.1371/journal.pone.0237227)
Supplement: S3 Table — This table summarizes all studies reporting mesoeconomic benefits and/or value (n = 27). (PDF) [file pone.0237227.s003.pdf]

S3 Table. Summary table of included studies reporting mesoeconomic benefits and/or value (n=27)

Author, year [country]	Aim/objective(s)	Population	Study type	Summary of main findings
(Aiken, Gomperts et al. 2017) [Ireland]	To examine the characteristics and experiences of women in Ireland and Northern Ireland seeking at-home medical termination of pregnancy (TOP) using online telemedicine	Women in Ireland and Northern Ireland who filled out an online consultation form requesting TOP between January 2010 and December 2015; follow up information for women to whom medications for TOP were shipped between 1 January 2010 and 31 December 2012	Analyses of online telemedicine for medical abortion (MA) services	The option of online telemedicine partly resolves the disparity in reproductive health and rights, addressing delays in accessing abortion care and situations where abortion care is rendered impossible.
(Benson, Gebreselassie et al. 2015) [Malawi]	To estimate current health system costs of treating unsafe abortion complications and compare these findings with newly-projected costs for providing safe abortion in Malawi	Malawi health system costs	Estimation study based on survey and costing data	In the 93 post-abortion care (PAC)-providing public facilities, two scenarios shifting treatment of abortion complications to legal, safe abortion both resulted in substantial annual cost savings: (1) If all women seeking first-trimester induced abortion choose manual vacuum aspiration (MVA), it results in an estimated cost reduction of 20%; (2) Making both MVA and misoprostol available results in an estimated 30% reduction. A liberalized abortion law and access to safe abortion in public health facilities yielded a 20-30 % decrease in current PAC costs.
(Billings and Benson 2005) [Latin America and the Caribbean]	To review results from 10 major postabortion care (PAC) operations research projects conducted in public sector hospitals in seven Latin American countries, completed and published between 1991 and 2002	Review of results of 10 PAC operation research projects in seven countries	Review	In studies from Mexico-Oaxaca and Peru-Callao, sharp curettage was used to treat all women with abortion complications prior to the PAC intervention. Following provider training, staff supervision, and technical assistance, use of MVA increased to 78% and almost 90% of patients, respectively. Approximately 3 years after the original intervention in Peru-Callao, 99% of all eligible women continued to be treated with MVA, demonstrating the sustainability of the service.
(Brack, Rochat et al. 2017) [Colombia]	To identify the key barriers to legal abortion, and to explore the ways they may work separately and together to delay the receipt of high quality, legal abortion care	Women aged 18 or older who had obtained a legal abortion in Bogotá, Colombia in the past 12 months and exhibited verbal proficiency in Spanish (n=17)	In-depth interviews	Insurance representatives hung up abruptly when participants mentioned abortion, did not return participants phone calls, or told participants that abortion was not covered. Inconsistent instructions regarding necessary authorizations further delayed women from getting approval and obtaining an abortion in a timely manner. Insurance companies acted as a barrier to timely access to legal, safe abortion care,

Author, year [country]	Aim/objective(s)	Population	Study type	Summary of main findings
				even though they are legally obligated to authorize the procedure. Religious sentiments appeared to underlie the behaviour of company representatives.
(Cheng, Zhou et al. 2012) [China]	To investigate providers' knowledge and attitudes about MA and their views regarding the main challenges to expanding the use of MA in urban and rural areas in China	Abortion service providers at the selected study sites, including ob-gyns, nurses, midwives, and family planning service providers who had been offering MA, surgical abortion, or both for more than 2 months	Multistage stratified cluster sampling design	Providers in the city of Shenzhen (38.2%) and rural Henan (33.1%) stated that a specific advantage of MA is that it is less expensive than surgical abortion. MA provides a low-cost, low-risk alternative to surgical abortion.
(Choobun, Khanuengkitkong et al. 2012) [Thailand]	To compare the hospital charges, duration of in-hospital procedures, clinical course and complications between MVA and sharp curettage	Women undergoing first-trimester abortions at the Songklanagarind Hospital (n=80)	Prospective observational study	This study confirms the benefit of MVA over sharp curettage in terms of less hospital visits, shorter duration of the procedure and hospitalization, less admissions, and less hospital-related expenditure.
(Comendant 2005) [Moldova]	To present information on the current abortion law, policy and services in Moldova and describe a project whose aim is to improve the quality of abortion services, including the introduction of MA through training of service providers and community education	Moldova	Mixed methods	Providers are pleased with the MVA services that are safe and effective, and save time and costs.
(Cook, de Kok et al. 2017) [Malawi]	To investigate factors contributing to the limited and declining use of MVA in Malawi	17 health workers of different cadres (doctors, nurses and clinical officers), genders and levels of experience and seniority who provided PAC in a central hospital and a district hospital	Small-scale qualitative study involving interviews supplemented by unstructured observations of care practices	Compared to D&C, MVA is cheaper, simpler and quicker to perform, requires less staff and allows for rapid discharge of patients resulting in fewer in-patients.
(Dennis, Manski et al. 2014) [United States]	To answer the following questions:<br>1) What do women know about the cost of abortion and the availability of Medicaid coverage for abortion?<br>2) Where do women obtain this information?<br>3) What are women's experiences paying for care?	Low-income women, defined as meeting the Medicaid income qualifications of the state where they had the abortion, aged 18 years or older who had an abortion within the past two years and who resided in one of the four study states at the time of the abortion	Two similarly-focused studies using in-depth interviews	In states where Medicaid coverage of abortion is available, a range of health care providers provide pregnancy options counselling, offer information about costs and coverage, and direct women to abortion clinics. The referrals in this study in Medicaid coverage states—where staff at abortion facilities refer women to Medicaid and where primary care providers refer women to abortion providers—indicate a health system responding to women's comprehensive health needs.

Author, year [country]	Aim/objective(s)	Population	Study type	Summary of main findings
(Díaz-Olavarrieta, Cravioto et al. 2012) [Mexico]	To identify the perceptions and opinions of people who provide abortion services in Mexico City, three years after implementation of elective abortion legal reforms	19 health workers assigned to the legal abortion programs at a clinic and a hospital in Mexico's Federal District	In-depth interviews	According to the ILE (legal pregnancy interruption) conscience objection is an exclusive right for medical doctors, but at times paramedic and administrative staff may assume themselves as objectors. Most participants mentioned that conscience objection is determined by cultural and religious views as well as by disinformation about reproductive health. A few medical doctors hold a double discourse because they refuse abortion practices in the public sphere but perform abortions in the private sphere, where it is more profitable.
(Gan, Zhang et al. 2011) [China]	To evaluate Chinese healthcare providers' knowledge of MA, to understand their perspectives regarding the main challenges to increasing its uptake, to understand their preferences for specific abortion methods, and to investigate the role of remuneration on the decision-making process	Profession/abortion providers (n=607)	Cross-sectional survey	Most providers said remuneration was not associated with recommending an abortion service. Only 17.8% of providers thought economic factors were important when recommending an abortion method. Overall, 10.5% considered provider income and 12.3% considered clinic income to be linked to the abortion method recommended. In 5.6% of cases, the clinic charged for abortion counselling, and in 10.9% of cases it charged for abortion follow-up. One hundred and fifteen healthcare providers (17.5%) stated that their income was related to the number of abortions they performed, and 72.2% reported that they did not receive any commission for providing abortion.
(Ganatra, Manning et al. 2005) [India]	To report on a study examining how mifepristone and misoprostol are being used in the Indian context; who is using them; how women access them; or how providers, chemists, women and their partners perceive MA	Chemists in the Indian states of Bihar and Jharkhand in 2004 (n=209)	Mixed methods: survey, in-depth interviews, observations	Some chemists thought that mifepristone and misoprostol were more effective than the other drugs; however, the belief that mifepristone was better was often due to its higher price with cost being a proxy for quality.
(Garcete and Winocur 2006) [Mexico]	To analyse different aspects that affect access to legal abortion in Mexico City and the characteristics that currently define such abortion services	Women who obtained or solicited legal abortion services between 2002-2006	Analysis of survey data	After the approval of the abortion law, the increase of induced abortion due to rape conducted by the Secretariat of Health of the Federal District during 2004 (n=13) was greater than the two previous years (n=5 in 2002, n=5 in 2003). Although the numbers are small, an increase of more than 100% is statistically significant.
(Gibb, Donaldson et al. 1998) [United	To measure women's preferences and strength of preferences	Women prior to having, and following, termination of early pregnancy (n=50)	Willingness to pay technique	If a choice had to be made about which service to provide, the willingness-to-pay and cost data indicate

Author, year [country]	Aim/objective(s)	Population	Study type	Summary of main findings
Kingdom]	(expressed in terms of willingness-to-pay) for MA versus surgical vacuum aspiration	at a gynaecology out-patient clinic in Aberdeen Royal Hospitals National Health Service Trust between August 1993 and February 1994		that the medical method should be chosen over surgical vacuum aspiration. As use of the medical method increases, the costs of maintaining the surgical facility may rise. However, a decision maker may still consider retaining this option on the grounds of equity.
(Hu, Grossman et al. 2009) [Mexico]	To assess the comparative health and economic outcomes associated with three alternative first-trimester abortion techniques in Mexico City and to examine the policy implications of increasing access to safe abortion modalities within a restrictive setting	Three alternative first-trimester abortion techniques in Mexico City	Computer-based model simulation of induced abortion and its potential complications Assessment of cost-effectiveness of alternative safe modalities for first-trimester pregnancy termination	In Mexico City in 2005, MA using vaginal misoprostol required four clinic visits: an initial screening and counselling visit, a visit to provide the medications and two follow-up visits. This protocol can likely be streamlined to two or three clinic visits, reducing the personal and direct medical cost of MA without impacting patient safety.
(Hu, Grossman et al. 2010) [Nigeria and Ghana]	To explore the policy implications of increasing access to safe abortion in Nigeria and Ghana<br><br>To comparatively assessed the cost-effectiveness of unsafe abortion and three first-trimester abortion modalities: hospital-based dilatation and curettage, hospital- and clinic-based MVA, and MA using misoprostol	A cohort of women in Ghana and Nigeria	Computer-based decision analytic model	Under a modified societal perspective, all safe abortion modalities were preferable to unsafe abortion and were associated with cost-savings relative to unsafe abortion. In Nigeria, clinic-based MVA was the least costly and most effective option. While equally as effective as clinic-based MVA, hospital-based MVA was the most expensive. Dilation and curettage (D&C) and MA using vaginal misoprostol provided comparable benefits although the latter was less costly. In contrast, in Ghana, MA was the most cost-effective option owing to its low procedural cost (approximately one-third the cost of clinic-based MVA). Clinic-based MVA was more effective than MA but was associated with a cost of $16,855 per life-year gained. Hospital-based MVA and D&C were more expensive and no more effective than clinic-based MVA, and were therefore strongly dominated.<br><br>Only 60% of all elective abortions in Nigeria are estimated to occur in public or private health facilities, mostly with D&C. A transition in practice pattern from one where 50% of women that would otherwise receive D&C to clinic-based MVA, without changing the

Author, year [country]	Aim/objective(s)	Population	Study type	Summary of main findings
				percentage that pursue unsafe abortion, saves substantial costs.<br><br>The provision of safe abortion in place of unsafe abortion is the single most important factor in improving health and economic outcomes associated with elective pregnancy termination. Clinic-based MVA is the most cost-effective surgical option for safe, first-trimester abortion. Wherever possible, transitioning from D&C to clinic-based MVA will result in lower costs, and reduce complications and deaths. MA will reduce health care costs and should be promoted as a nonsurgical option for elective abortion.
(Ilboudo, Greco et al. 2016) [Burkina Faso]	To estimate the costs of six abortion complications including incomplete abortion, haemorrhage, shock, infection/sepsis, cervix or vagina laceration, and uterus perforation treated in two public referral hospital facilities in Ouagadougou and the cost saving of providing safe abortion care services	Records of patients seeking PAC at two public referral hospitals in Ouagadougou (n=449)<br><br>Key informants (e.g., gynaecologist, midwife, administrative personnel) in maternity wards and in hospital facilities	Review of PAC-registers using two structured questionnaires and key informant interviews	If safe abortion care services were available in 2010, the provision of these services would only have cost US$ 2,694 for both referral hospital facilities (representing only 12% of the facilities' total costs spent treating complications of unsafe abortions). An estimated US$ 19,778.53 would thus have been saved in 2010, if safe abortion care services were available.
(Johnston, Ved et al. 2003) [India]	To obtain information about where rural women seek care for abortion complications and about the quality of care they receive	Surveys of formal and informal organizations, institutions and key leaders in four selected villages in rural Uttar Pradesh<br><br>Community mapping exercises in four selected villages in rural Uttar Pradesh<br><br>24 focus group discussions with informants from specific population subgroups, including married women and men, and single female and male young adults<br><br>88 in-depth interviews with 53 key informants who were married women that the interviewers identified as being	Descriptive surveys, community mapping exercises, focus group discussions, and in-depth interviews	Village-level providers have an opportunity to engage their patients in a formal network of care. The providers identified in the study, all of whom already provide PAC in some form, could contribute to decreasing maternal morbidity and mortality from unsafe abortion by appropriately referring women to higher-level care for abortion and PAC services; providing contraceptive counselling and methods, or referral; and linking PAC patients to reproductive health and other services as necessary.

Author, year [country]	Aim/objective(s)	Population	Study type	Summary of main findings
		particularly knowledgeable about abortion and PAC issues in their village<br><br>In-depth interviews with 38 PAC providers		
(Jowett 2000) [low-income countries]	To review evidence on safe motherhood from low-income countries	Evidence from low-income countries	Review	In Kenya, switching from sharp curettage to MVA saved 38% in equipment costs and reduced hospital stays by 41-76%. In Brazil, incomplete abortion patients treated with MVA consumed 41% fewer resources than similar patients treated with sharp curettage.
(Levin, Grossman et al. 2009) [Mexico]	To assess abortion outcomes and costs to the health care system in Mexico City in 2005 at a mix of public and private facilities prior to the legalisation of abortion	Hospital staff, administrative records and patients at three public hospitals and one private clinic in Mexico City	Cost estimates and projections	Reducing complications by improving access to safe services in outpatient settings would reduce the costs of abortion care, with significant benefits to Mexico's health care system. Even a modest increase in access to MVA would provide additional cost savings of over US$ 50,000. Key to these scenarios is that the cost of providing abortion services declines as access increases at smaller public and private health facilities that can provide abortion care efficiently and at less cost than large hospitals.
(Pillai, Welsh et al. 2015) [United Kingdom]	To assess the applicability, acceptability and cost implications of introducing the MVA technique with local anaesthesia for fully conscious first-trimester termination of pregnancy within the National Health Service and for our population	Women attending the Pregnancy Advisory Service	Analysis of routinely collected data	The main cost savings were realised by replacing surgical procedures in theatre with MVA. Within four months of introducing MVA, it was possible to replace one of three weekly theatre lists with MVAs. The saving on the theatre recharge was £92,000 per annum. The costs for setting up and running the MVA sessions amounted to £28,000 per annum. The main consumable cost was contraception. Owing to the high uptake of long-acting reversible contraceptives, particularly the 51% uptake of the intrauterine system, this averaged £235 per session of four cases. There were additional setup costs for purchase of dilator sets and theatre clothing. Overall, the difference in cost of the weekly theatre list against running two MVA sessions per week resulted in an annual saving of around £60,000.
(Rodriguez, Mendoza et al. 2015) [Colombia]	To compare the costs to the health system of three approaches to the provision of abortion care in Colombia: PAC for complications of	Women who sought abortion care at all three sites in 2012 (n=1,411)	Cost analysis	Legal abortion would need to cost more than $734 per case for PAC to be a cost-saving strategy. If we assume a 9% spontaneous abortion rate, and if the remainder of PAC cases currently observed were replaced with legal

Author, year [country]	Aim/objective(s)	Population	Study type	Summary of main findings
	unsafe abortions, and for legal abortions in a health facility, misoprostol-only MA and vacuum aspiration abortion			abortion (medical or MVA), the health system would save an additional $163,000 dollars and prevent 16 complications per 1,000 abortions. The health system could save an additional $177,000 (per 1,000 women) from baseline, by replacing D&C with MVA.
(Schaff and Schaff 2010) [United States]	To review evidence relating to mifepristone ten years after its legalization in the United States	Evidence on mifepristone across the United States	Review	The Food and Drug Administration recommended a mifepristone dose of 600mg. This dosage is expensive, and some facilities require four visits before administering it, thus reducing the cost benefits.
(Sjostrom, Kopp Kallner et al. 2016) [Sweden]	To calculate the cost-effectiveness of early MA performed by nurse-midwifes in comparison to physicians in a high resource setting where ultrasound dating is part of the protocol	Healthy women seeking treatment for abortion at an outpatient clinic of a university hospital	Cost effectiveness analysis	Task shifting of MA generated direct economic benefits associated with the shorter time spent in the clinic by providers and patients and nurse-midwives' lower salaries. Costs were also reduced due to shorter waiting time for subsequent patients and a possible lower cost for the treatment of complications. The intervention was superior in efficacy and cheaper than the standard treatment. In addition, the costs of the intervention are expected to decrease over time.
(Thapa, Poudel et al. 2004) [Nepal]	To assess and evaluate the safety, acceptability, and effectiveness of MVA services	Two groups of patients were compared: 529 patients treated in the MVA unit and 236 patients who were clinically eligible for treatment in the MVA unit but were treated instead in the main operation theatre (OT) owing to the unavailability of services in the MVA unit during the hours of their admission	Cohort analytic	OT patients spent, on average, 33.42 hours in the facility, whereas the MVA patients spent only 19.75 hours. Thus, OT patients would have stayed 58.6% fewer hours in the hospital had they been treated in the MVA unit. Since the hospital usually has an overflow of patients, the freed-up beds would have been put to use by other patients. In addition, the hospital would have been able to use the physicians and anaesthetists for other major surgical cases. Switching eligible patients to the MVA unit and, thereby, shortening their stay in hospital, however, would not necessarily have reduced the cost to those patients. The cost of treatment in the MVA unit was about the same as treatment in the OT. We also estimated the potential use of the MVA unit if its service hours could be extended from 7 to 24 hours per day. Of the total number of patients with post-abortion complications, nearly three quarters could be treated in the MVA unit.
(Upadhyay, Johns et al. 2017) [United	To examine the association between distance travelled for an abortion	Women seeking abortion services who were beneficiaries of the California	Retrospective observational cohort	This study supports fully reimbursing abortion providers for all forms of follow-up care (e.g., low-sensitivity or

Author, year [country]	Aim/objective(s)	Population	Study type	Summary of main findings
States]	and site of PAC among low-income women	Medicaid program	study	semiquantitative urine pregnancy tests, phone calls, or internet communication) after medication abortion. Encouraging these alternative forms of follow-up care will likely result in cost-savings because women may be less likely to seek such care at emergency departments.
(Winocur 2006) [Mexico]	To analyse abortion laws and access to justice in the light of individual cases	Women of different ages who sought abortion services because of rape incidents and who faced several barriers to access the service and who obtained different results	Legal analysis and interviews	Authorities put up multiple barriers for women who come to request a legal interruption of their pregnancy, leading these women to look for clandestine procedures. An investigation by Lara, Strickler, Ellertson and Tsuyuki (2003) showed that 18% of "clandestine" abortions are performed for reasons considered legal and another 20% of these abortions are done for economic reasons, a circumstance that is only decriminalized in Yucatán.

References

Aiken, A., R. Gomperts and J. Trussell (2017). "Experiences and characteristics of women seeking and completing at-home medical termination of pregnancy through online telemedicine in Ireland and Northern Ireland: a population-based analysis." Bjog **124**(8): 1208-1215.

Benson, J., H. Gebreselassie, M. A. Manibo, K. Raisanen, H. B. Johnston, C. Mhango and B. A. Levandowski (2015). "Costs of postabortion care in public sector health facilities in Malawi: a cross-sectional survey." BMC Health Serv Res **15**: 562.

Billings, D. L. and J. Benson (2005). "Postabortion care in Latin America: policy and service recommendations from a decade of operations research." Health Policy Plan **20**(3): 158-166.

Brack, C. E., R. W. Rochat and O. A. Bernal (2017). "It's a Race Against the Clock: A Qualitative Analysis of Barriers to Legal Abortion in Bogot, Colombia." International Perspectives on Sexual and Reproductive Health **43**(4): 173-182.

Cheng, Y., Y. Zhou, Y. Zhang, X. Jiang, M. Xi, K. Gan and S. Ren (2012). "Study of knowledge and attitudes on medical abortion among Chinese health providers." International Journal of Gynecology & Obstetrics **118**: S28-S32.

Choobun, T., S. Khanuengkitkong and S. Pinjaroen (2012). "A comparative study of cost of care and duration of management for first-trimester abortion with manual vacuum aspiration (MVA) and sharp curettage." Archives of Gynecology & Obstetrics **286**(5): 1161-1164.

Comendant, R. (2005). "A project to improve the quality of abortion services in Moldova." Reproductive Health Matters **13**(26): 93-100.

Cook, S., B. de Kok and M. L. Odland (2017). "'It's a very complicated issue here': understanding the limited and declining use of manual vacuum aspiration for postabortion care in Malawi: a qualitative study." Health Policy & Planning **32**(3): 305-313.

Dennis, A., R. Manski and K. Blanchard (2014). "Does Medicaid Coverage Matter? A Qualitative Multi-State Study of Abortion Affordability for Low-income Women." Journal of Health Care for the Poor & Underserved **25**(4): 1571-1585.

Díaz-Olavarrieta, C., V. M. Cravioto, A. Villalobos, N. Deeb-Sossa, L. García and S. G. García (2012). "Mexico City's Legal Abortion Program: health workers' experiences." Revista Panamericana de Salud Publica **32**(6): 399-404.

Gan, K., Y. Zhang, X. Jiang, Y. Meng, L. Hou and Y. Cheng (2011). "Perspectives of Chinese healthcare providers on medical abortion." International Journal of Gynecology & Obstetrics **114**(1): 15-17.

Ganatra, B., V. Manning and S. P. Pallipamulla (2005). "Availability of medical abortion pills and the role of chemists: a study from Bihar and Jharkhand, India." Reproductive Health Matters **13**(26): 65-74.

Garcete, N. U. and M. Winocur (2006). "El acceso al aborto por violación en la Ciudad de México: limitaciones, oportunidades y desafíos." Debate Feminista **34**: 162-184.

Gibb, S., C. Donaldson and R. Henshaw (1998). "Assessing strength of preference for abortion method using 'willingness to pay': a useful research technique for measuring values." Journal of Advanced Nursing **27**(1): 30-36.

Hu, D., D. Grossman, C. Levin, K. Blanchard, R. Adanu and S. J. Goldie (2010). "Cost-effectiveness analysis of unsafe abortion and alternative first-trimester pregnancy termination strategies in nigeria and ghana." African Journal of Reproductive Health **14**(2): 85-103.

Hu, D., D. Grossman, C. Levin, K. Blanchard and S. Goldie (2009). "Cost-effectiveness analysis of alternative first-trimester pregnancy termination strategies in Mexico City." BJOG: An International Journal of Obstetrics & Gynaecology **116**(6): 768-779.

Ilboudo, P. G. C., G. Greco, J. Sundby and G. Torsvik (2016). "Estimating the costs for the treatment of abortion complications in two public referral hospitals: a cross-sectional study in Ouagadougou, Burkina Faso." BMC Health Services Research **16**: 1-10.

Johnston, H. B., R. Ved, N. Lyall and K. Agarwal (2003). "Where do rural women obtain postabortion care? The case of Uttar Pradesh, India." International Family Planning Perspectives **29**(4): 182-187.

Jowett, M. (2000). "Safe motherhood interventions in low-income countries: an economic justification and evidence of cost effectiveness." Health Policy **53**(3): 201-228.

Levin, C., D. Grossman, K. Berdichevsky, C. Diaz, B. Aracena, S. G. Garcia and L. Goodyear (2009). "Exploring the costs and economic consequences of unsafe abortion in Mexico City before legalisation." Reproductive Health Matters **17**(33): 120-132.

Pillai, M., V. Welsh, K. Sedgeman, A. C. Gazet, J. Staddon and H. Carter (2015). "Introduction of a manual vacuum aspiration service: a model of service within a NHS Sexual Health Service." Journal of Family Planning & Reproductive Health Care **41**(1): 27-32.

Rodriguez, M. I., W. S. Mendoza, C. Guerra-Palacio, N. A. Guzman and J. E. Tolosa (2015). "Medical abortion and manual vacuum aspiration for legal abortion protect women's health and reduce costs to the health system: findings from Colombia." Reproductive Health Matters **22**: 125-133.

Schaff, E. A. and E. A. Schaff (2010). "Mifepristone: ten years later." Contraception **81**(1): 1-7.

Sjostrom, S., H. Kopp Kallner, E. Simeonova, A. Madestam and K. Gemzell-Danielsson (2016). "Medical Abortion Provided by Nurse-Midwives or Physicians in a High Resource Setting: A Cost-Effectiveness Analysis." PLoS One **11**(6): e0158645.

Thapa, S., J. Poudel and S. Padhye (2004). "Triaging patients with post-abortion complications: a prospective study in Nepal." J Health Popul Nutr **22**(4): 383-398.

Upadhyay, U. D., N. E. Johns, K. R. Meckstroth and J. L. Kerns (2017). "Distance Traveled for an Abortion and Source of Care After Abortion." Obstetrics & Gynecology **130**(3): 616-624.

Winocur, M. (2006). "Paulina, un caso paradigmático." Debate Feminista **34**: 185-205.
